# Azetidin-2-ones: structures of anti­mitotic compounds based on the 1-(3,4,5-tri­meth­oxy­phen­yl)azetidin-2-one core

**DOI:** 10.1107/S2056989020008555

**Published:** 2020-07-03

**Authors:** Brendan Twamley, Niamh M. O’Boyle, Mary J. Meegan

**Affiliations:** a School of Chemistry, Trinity College Dublin, Dublin 2, Ireland; b School of Pharmacy and Pharmaceutical Sciences, Trinity Biomedical Sciences Institute, Trinity College Dublin, 152 - 160 Pearse St, Dublin 2, Ireland

**Keywords:** β-Lactam, anti­mitotic, crystal structure

## Abstract

A series of related substituted 1-(3,4,5-tri­meth­oxy­phen­yl)azetidin-2-ones have been characterized: 3-(4-fluoro­phen­yl)-4-(4-meth­oxy­phen­yl)-1-(3,4,5-tri­meth­oxy­phen­yl)azetidin-2-one, C_25_H_24_FNO_5_ (**1**), 3-(furan-2-yl)-4-(4-meth­oxy­phen­yl)-1-(3,4,5-tri­meth­oxy­phen­yl)azetidin-2-one, C_23_H_23_NO_6_ (**2**), 4-(4-meth­oxyphen­yl)-3-(naphthalen-1-yl)-1-(3,4,5-tri­meth­oxy­phen­yl)azetidin-2-one, C_29_H_27_NO_5_ (**3**), 3-(3,4-di­meth­oxy­phen­yl)-4-(4-meth­oxy­phen­yl)-1-(3,4,5-tri­meth­oxy­phen­yl)azetidin-2-one, C_27_H_29_NO_7_ (**4**) and 4,4-bis­(4-meth­oxy­phen­yl)-3-phenyl-1-(3,4,5-tri­meth­oxy­phen­yl)azetidin-2-one, C_32_H_31_NO_6_ (**5**). All of the compounds are racemic. The lactam and 3,4,5-tri­meth­oxy­phenyl rings are approximately co-planar and the orientation of the lactam and the 4-meth­oxy­phenyl substituent is approximately orthogonal. The chiral centres, although eclipsed by geometry, have torsion angles ranging from −7.27 to 13.08° for the 3 position, and −8.69 to 13.76° for the 4 position of the β-lactam. The structures display intra­molecular C—H⋯O bonding between the 3,4,5-tri­meth­oxy­phenyl ring and the lactam ketone. Further C—H⋯O inter­actions are observed and form either an opposing meth­oxy ‘buckle’ to join two mol­ecules together or a cyclic dimer.

## Abstract 2

Five azetidin-2-ones are described based on the 1-(3,4,5-tri­meth­oxy­phen­yl)azetidin-2-one core with different substituents on the lactam 3 and 4 positions: (**1**) 3-(4-fluoro­phen­yl)-4-(4-meth­oxy­phen­yl); (**2**) 3-(furan-2-yl)-4-(4-meth­oxy­phen­yl); (**3**) 3-(naphthalen-1-yl)-4-(4-meth­oxy­phen­yl); (**4**) 3-(3,4-di­meth­oxy­phen­yl)-4-(4-meth­oxy­phen­yl); (**5**) 4,4-bis­(4-meth­oxy­phen­yl)-3-phenyl-1-(3,4,5-tri­meth­oxy­phen­yl)azetidin-2-one.

## Chemical context   

β-Lactam anti­biotics *e.g*. penicillins, cephalosporins, carbapenems and monobactams, are based on a core β-lactam ring structure and play a significant role in the clinical treatment of bacterial infections (Kong *et al.*, 2010 ▸). Their mechanism of action is by targeting the transpeptidase enzymes (penicillin-binding proteins), which are required for bacterial cell-wall synthesis. However, because of extensive use, many bacteria have developed resistance to β-lactam anti­biotics. Additionally, the anti­proliferative activity of compounds containing the β-lactam (azetidin-2-one) ring structure has been investigated (Zhou *et al.* 2018 ▸; Galletti *et al.* 2014 ▸; Geesala *et al.*, 2016 ▸; Arya *et al.*, 2014 ▸; Fu *et al.*, 2017 ▸). We have previously demonstrated the effectiveness of 1,4-di­aryl­azetidin-2-ones in breast-cancer cell lines as tubulin-targeting anti­mitotic agents and selective estrogen-receptor modulators (SERMs; O’Boyle *et al.*, 2014 ▸). β-Lactams are also useful as synthetic inter­mediates in organic synthesis (Kamath & Ojima, 2012 ▸).

To further increase our library of β-lactam anti­mitotic compounds, we have investigated the systematic synthesis and activity of a range of different β-lactams based on the 1-(3,4,5-tri­meth­oxy­phen­yl) β-lactam core (O’Boyle *et al.* 2010 ▸, 2011*a* ▸,*b*). The five structurally characterized azetidin-2-ones reported herein have all been included in studies as tubulin-targeting agents with mitotic catastrophe. They have all displayed good anti­proliferative effects in MCF-7 human breast-cancer cells, but tuning the substitution pattern in the aromatic ring C atoms has produced more efficacious azetidinones for further testing. The structural study of these compounds has been challenging, as the yields from synthesis were low, hence obtaining suitable crystalline samples was difficult. These structures will enable further modelling to improve the design of more effective β-lactam anti­biotics.
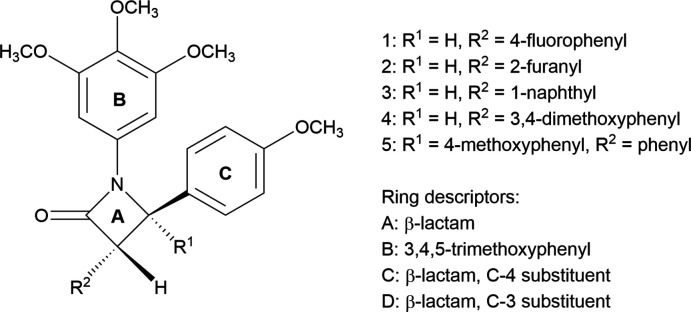


## Structural commentary   

Compound **1** crystallizes in the ortho­rhom­bic system, **2** and **4** in the monoclinic and **3** and **5** in the triclinic system. It is clear from the space group that these chiral mol­ecules have crystallized as conformational racemates.

**Figure 1:**
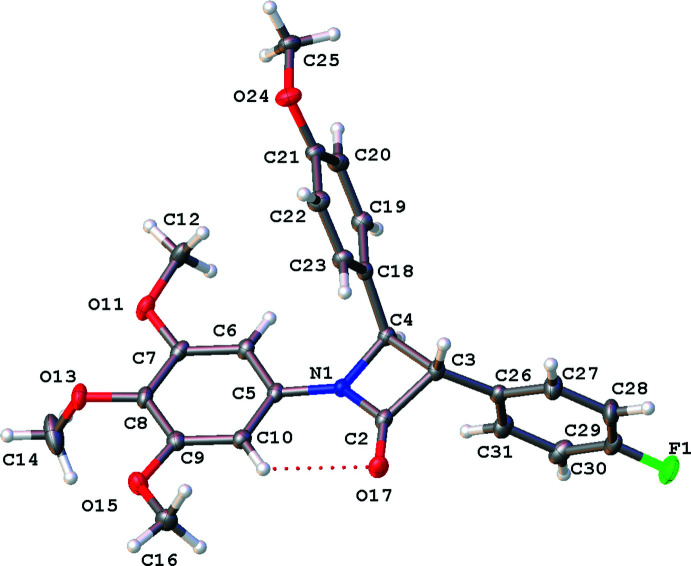
Mol­ecular structure of **1**, relative stereochemistry 3*S*, 4*R*, with displacement ellipsoids drawn at the 50% probability level. Hydrogen atoms shown as spheres of arbitrary radius.

The mol­ecules are shown in Figs. 1 ▸–5 ▸ ▸ ▸ ▸. Bond lengths and angles fall within reported limits. From Table 1 ▸ it can be seen that there are some commonalities in the structures, despite the differences in chirality and substituents. The common 3,4,5-tri­meth­oxy­phenyl rings and the carbonyl of the lactam display an intra­molecular hydrogen bonds (C10⋯O17, see Table 2 ▸), which orient the *A* and *B* rings to be approximately co-planar with angles of 2.62 (13)–17.08 (9)° between ring plane normals (see Table 1 ▸). The *A* and *B* rings can also twist and flex along the N1–C5 vector, which can also be seen in the C2—N1—C5—C10 torsion angle (see Table 1 ▸). See Fig. 6 ▸ for an overlay of similar conformations of **1**–**5** normal to the plane of the lactam.

It can also be seen that for both mono and di-substituted C4 lactams the angle between the lactam *A* and the *C* ring (C18–C23) is approximately orthogonal with angles ranging from 83.59 (9) to 89.56 (8)°.

The conformations of the chiral centres at C3 in **1**–**5** are approximately eclipsed by geometry of the *sp* ^3^ carbons in the lactam ring with H3—C3—C4—C18 angles ranging from 0.98° in the *R* isomer of **5** (di-substituted in the 4 position – more steric requirements), to a wider 13.08° in **4**. The conformation at C4 is also partially eclipsed with H4—C4—C3—C26 angles of 4.96° in **3** and the largest angle of 13.76° in **4** (see Table 1 ▸).

### 3-(4-Fluoro­phen­yl)-4-(4-meth­oxy­phen­yl)-1-(3,4,5-tri­meth­oxy­phen­yl)azetidin-2-one, 1   

There is a single mol­ecule of **1** in the asymmetric unit in the ortho­rhom­bic centrosymmetric space group *Pbca*, see Fig. 1 ▸. The compound is a racemate and the relative stereochemistry shown is 3*S*, 4*R*. In this compound, the *A*|*D* ring plane normal angle is close to 90° (Table 1 ▸). A similar, recently published structural isomer (Malebari *et al.*, 2020 ▸; CSD refcode PUKNUH) is also a racemate and has two independent enanti­omers in the asymmetric unit. The major structural difference between **1** and PUKNUH is the orientation of the tri­meth­oxy­phenyl ring plane to the lactam ring [8.87 (4)° plane normals for lactam N1 in PUKNUH, with the same chirality]. It can also be seen in the C2—N1—C5—C10 torsion angle of −4.3 (3) for **1** (see Table 1 ▸) and 11.9 (2)° for the N1 lactam in PUKNUH where, in spite of the hydrogen bond between the ring and the lactam carbonyl, the substituted *B* rings are orientated differently and the 4-meth­oxy groups on this ring are oriented in opposite directions (see Fig. S1 in the supporting information).

### 3-(Furan-2-yl)-4-(4-meth­oxy­phen­yl)-1-(3,4,5-tri­meth­oxy­phen­yl)azetidin-2-one, 2   

**Figure 2:**
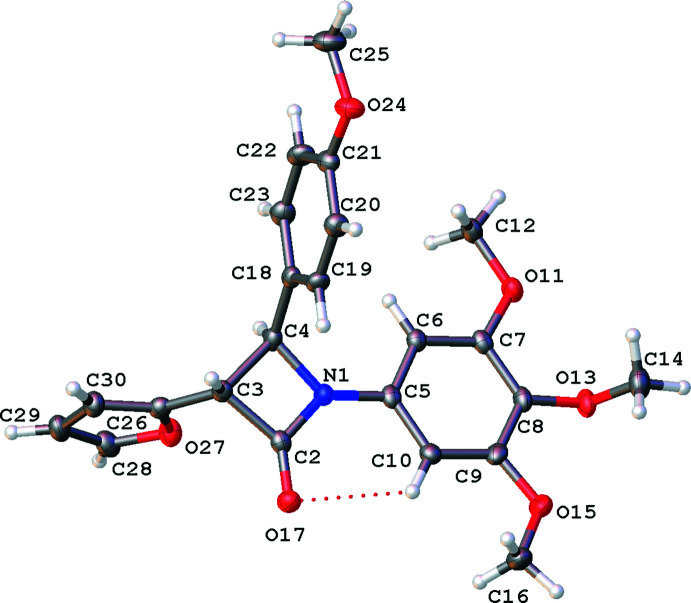
Mol­ecular structure of one of the unique mol­ecules in the asymmetric unit of **2**, relative stereochemistry 3*S*, 4*S*, with displacement ellipsoids drawn at the 50% probability level. Hydrogen atoms shown as spheres of arbitrary radius.

Compound **2** has two independent mol­ecules in the asymmetric unit in the monoclinic centrosymmetric system *P*2_1_/*c*, and only one mol­ecule is displayed in Fig. 2 ▸. In this racemic compound, both *trans* diastereomers are seen and the relative stereochemistry is 3*S*, 4*S* for the lactam with N1 and 3*R*, 4*R* for the lactam with N1*A*. See Table 1 ▸ for the geometric parameters. A comparison of the two independent mol­ecules in **2** show similar differences as seen above – differences in the orientation of ring *B* to the lactam *A* ring (See Table 1 ▸ for *A*|*B* ring plane normals and the torsion angle C2—N1—C5 —C10) as well as the difference in orientation the 4-meth­oxy group position on the *B* ring (see Fig. S2 in the supporting information). The other notable difference is the orientation of the *D* rings to the lactam. In the N1 mol­ecule (relative stereochemistry 3*S*, 4*S*) the *A*|*D* plane normals angle is approximately orthogonal (see Table 1 ▸). However in the other conformation (N1*A*, relative stereochemistry 3*R*, 4*R*) this *A*|*D* angle is much more acute. The twist of the group is also reflected in the torsion angles C2—C3—C26—O27 = −43.8 (3)° and C2*A*—C3*A*—C26*A*—O27*A* = 180.0 (2)°. There are no significant inter­actions to the furan directing this change.

### 3-(Naphthalen-1-yl)-4-(4-meth­oxy­phen­yl)-1-(3,4,5-tri­meth­oxy­phen­yl)azetidin-2-one, 3   

**Figure 3:**
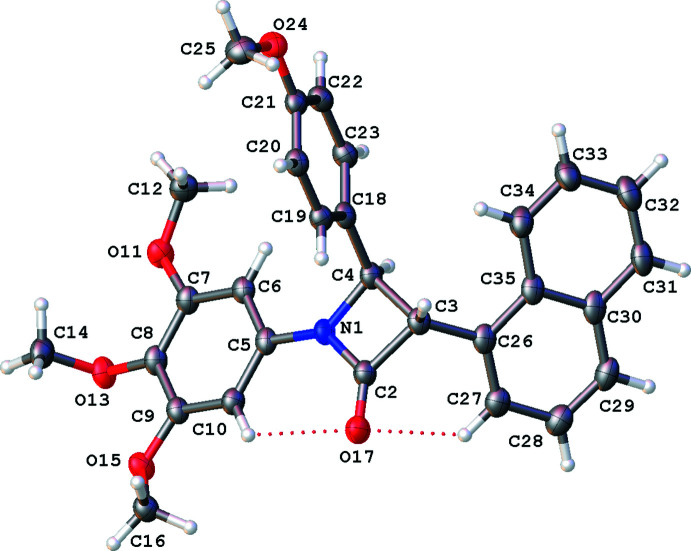
Mol­ecular structure of **3**, relative stereochemistry 3*S*, 4*R*, with displacement ellipsoids drawn at the 50% probability level. Hydrogen atoms shown as spheres of arbitrary radius.

The structure of **3**, triclinic *P* 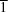, with one mol­ecule in the asymmetric unit, is similar to that of **1**, see Fig. 3 ▸, and displays the common features mentioned above. However, in this case, the *D* ring substituent, naphthalene, also forms a hydrogen bond with the lactam ketone (C27⋯O17, see Table 2 ▸) and the *D* ring is not orthogonal to the lactam as in **1** (see Table 1 ▸) and has a C2—C3—C26—C27 torsion angle of −13.3 (3)°.

### 3-(3,4-Di­meth­oxy­phen­yl)-4-(4-meth­oxy­phen­yl)-1-(3,4,5-tri­meth­oxy­phen­yl)azetidin-2-one, 4   

**Figure 4:**
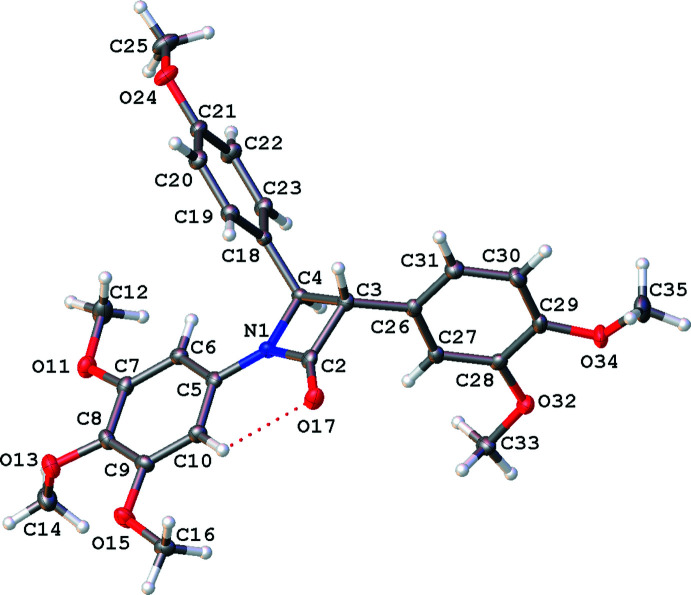
Mol­ecular structure of **4**, relative stereochemistry 3*S*, 4*R*, with displacement ellipsoids drawn at the 50% probability level. Hydrogen atoms shown as spheres of arbitrary radius.

Compound **4**, is similar to both **1** and **3** with one mol­ecule in the asymmetric unit in the monoclinc space group *P*2_1_/*c*, see Fig. 4 ▸. The *A*|*B* ring plane normals angle and C2—N1—C5—C10 torsion angles are small, and the *C* and *D* rings are essentially orthogonal to the lactam (see Table 1 ▸). Showing all the commonalities described above, the main difference in **4** is seen in the dihedral angle along the C3–C4 vector, as this mol­ecule displays the largest angle for H3—C3—C4—C26/H4—C4—C3—C26 (see Table 1 ▸).

### 3-Phenyl-4,4-bis­(4-meth­oxy­phen­yl)-1-(3,4,5-tri­meth­oxy­phen­yl)azetidin-2-one, 5   

**Figure 5:**
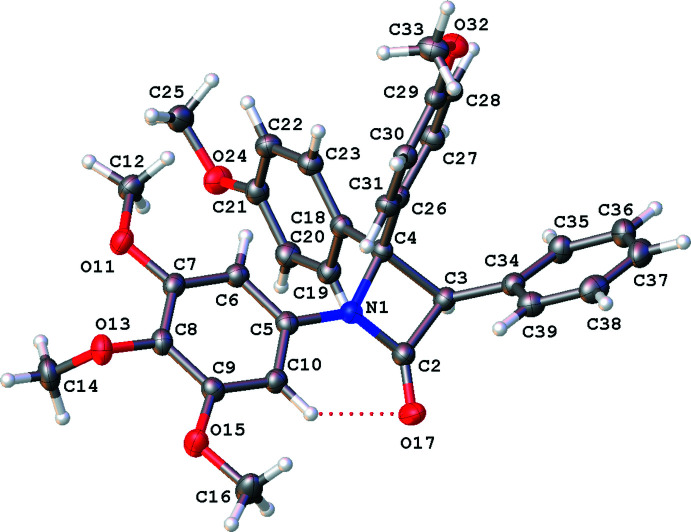
Mol­ecular structure of one of the unique mol­ecules in the asymmetric unit of **5**, relative stereochemistry 3*R*, with displacement ellipsoids drawn at the 50% probability level. Hydrogen atoms shown as spheres of arbitrary radius.

**Figure 6:**
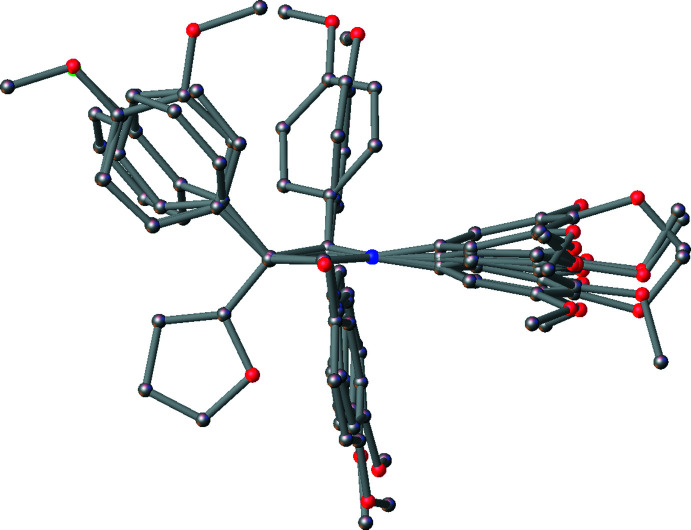
Overlay of similar diastereomers of **1-**-**5** normal to the plane of the lactam. The N1, C2 and O17 atoms were used as overlay centres. The flexibility in orientation of the *B* ring relative to the *A* ring (lactam) is clearly seen, as well as the substituents of the *C* and *D* rings.

Compound **5**, with two independent mol­ecules, one of each enanti­omer in the asymmetric unit in the triclinic space group *P* 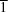, is a more unusual β-lactam in that there are two identical substituents on the 4 position, see Fig. 5 ▸ where only one of the racemic mol­ecules is shown. The torsion angles H3—C3—C4—C18 and C26—C4—C3—C34 [−5.62 (2) and 2.56 (2)°] in both enanti­omers show that the arrangement is the most eclipsed among **1**–**5**. Compound **5** also shows the largest *A*|*B* plane normal angles, indicating a bending along the N1–C5 vector and the trimeth­oxy ring and lactam are twisted as seen in the large C2—N1—C5—C10 torsion angles (Table 1 ▸). While showing all the common features outlined above, this mol­ecule displays a conformational difference in the 4-meth­oxy group on the *B* ring between each enanti­omer, also seen in **2** and shown in Fig. S3. This is the only example of a 4,4′-disubstituted 1-(3,4,5-tri­meth­oxy­phen­yl) β lactam. As a result of steric requirements, the 4 and 4′ substituents in both mol­ecules show a substantial difference in *A*|*C* plane normals. Other non-bicyclic 4,4′-disubstituted β lactams are known (see *Database survey*, Table 3 ▸). Only AHERUA, which has phenyl substitutents, shows similar steric demands with equivalent C2—N1—C5—C10 torsion angles of *ca* 10.7° and *A*|*C* plane normal angles of 81.066 (1)° and 61.454 (1)°. RIFYIO has different steric requirements with meth­oxy­carbonyl­phenyl­ethyl and acetyl groups on N1 and C3 respectively. C4 is diphenyl substituted with *A*|*C* angles of 76.79 (5)° and 66.21 (5)°.

## Supra­molecular features   

**Figure 7:**
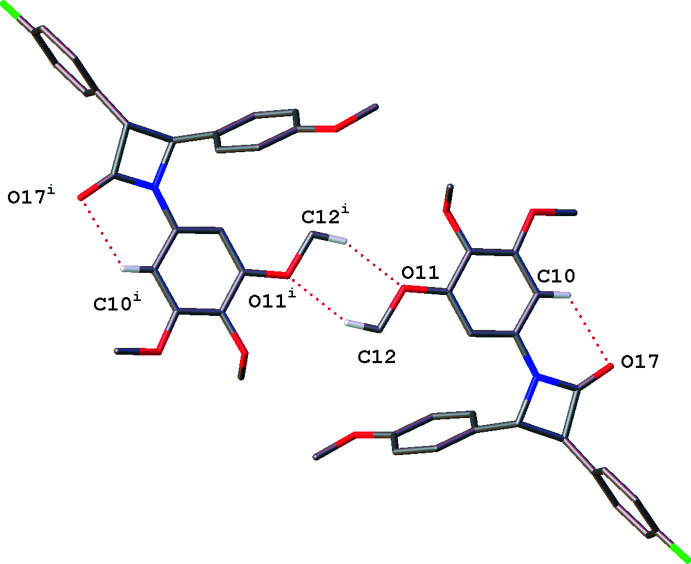
Hydrogen-bonding ‘buckle’ motif seen in **1**. Only hydrogen atoms involved in intra- and inter­molecular hydrogen bonding are shown. Dotted lines indicate hydrogen-bonding inter­actions. [Symmetry code: (i) *x* + 1, −*y* + 1, −*z* + 2].

As well as the intra­molecular hydrogen-bonding pattern described above, with the number of meth­oxy groups present, there are many weak C—H⋯O inter­molecular inter­actions in **1**–**5**. The most significant are shown in Table 2 ▸. A motif seen in **1** is an association with two opposing meth­oxy groups, see Fig. 7 ▸, which form a ‘buckle’ to join two mol­ecules together.

**Figure 8:**
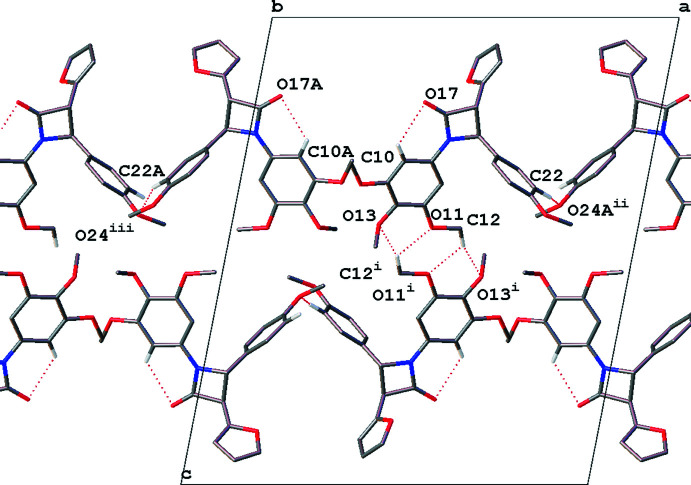
‘Double buckle’ hydrogen-bonding motif seen in **2**, with linking phenyl-meth­oxy hydrogen bonding viewed normal to the *b* axis. Only hydrogen atoms involved in intra- and inter­molecular hydrogen bonding are shown. Dotted lines indicate hydrogen-bonding inter­actions. [Symmetry codes: (i) −*x* + 1, −*y*, −*z* + 1; (ii) *x* + 1, *y* − 1, *z*; (iii) *x* − 1, *y*, *z*].

In **2** a ‘double buckle’ is present due to a bifurcated hydrogen bond between C12 and O11/O13 of an adjacent tri­meth­oxy­phenyl ring. These are then linked into a network *via* C22⋯O24*A* and C22*A*⋯O24 (see Table 2 ▸). This motif is shown in Fig. 8 ▸.

**Figure 9:**
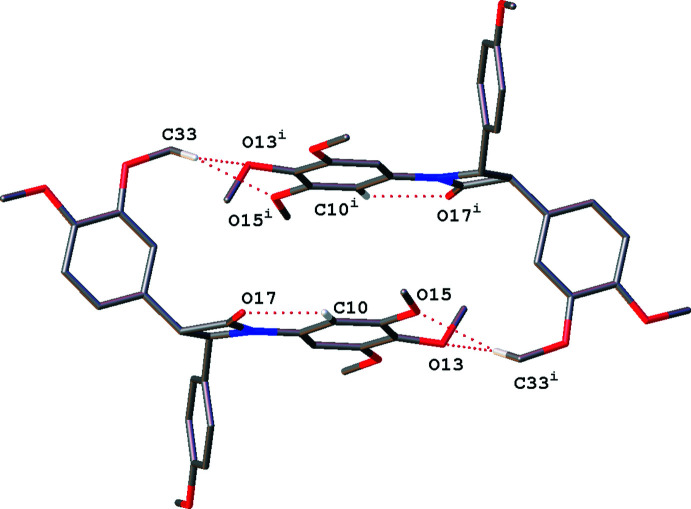
Dimer hydrogen-bonding motif seen in **4**, linked *via* a bifurcated meth­oxy–meth­oxy C—H⋯O inter­action. Only hydrogen atoms involved in intra- and inter­molecular hydrogen bonding are shown. Dotted lines indicate hydrogen-bonding inter­actions. [Symmetry code: (i) −*x* + 1, −*y* + 1, −*z* + 1].

In **4** the mol­ecules do not associate *via* the ‘buckle’ and instead form an end-to-end hydrogen-bonded cyclic dimer with a bifurcated hydrogen bond, see Fig. 9 ▸.

**Figure 10:**
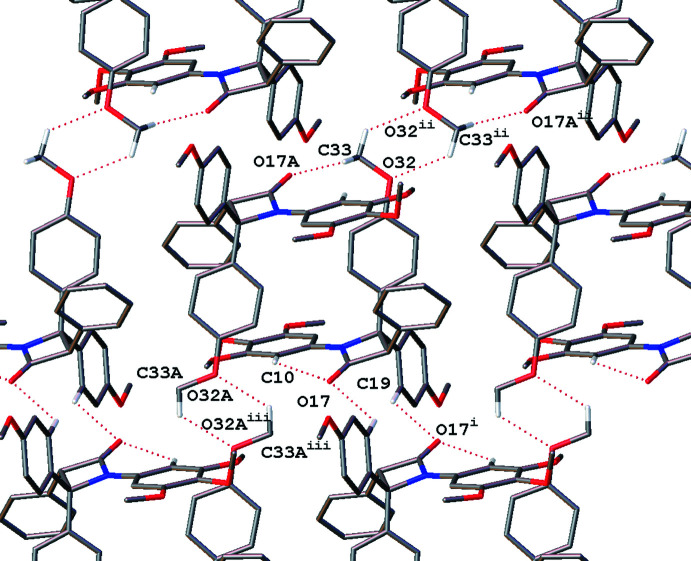
‘Buckle’ hydrogen-bonding motif seen in **5** with extra cross-linking inter­actions viewed normal to the *a* axis. Only hydrogen atoms involved in intra- and inter­molecular hydrogen bonding are shown. Dotted lines indicate hydrogen-bonding inter­actions. [Symmetry codes: (i) −*x* + 1, −*y*, 1 − *z*; (ii) −*x* + 1, −*y*, −*z*; (iii) −*x* + 1, −*y* + 1, −*z* + 1].

The ‘buckle’ association of **1** is also seen in both enanti­omers in **5**, with further C—-H⋯O inter­actions by the carbon of the meth­oxy group of one enanti­omer inter­acting with opposite enanti­omer ketone (Table 2 ▸). Adjacent like enant­iomers are also linked *via* C—H⋯O inter­actions with the *C* phenyl ring and the lactam ketone, forming an inter­connected sheet parallel to the *bc* plane, see Fig. 10 ▸.

**Figure 11:**
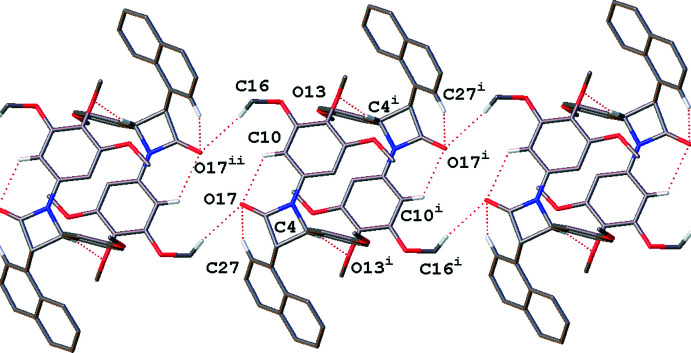
Dimer hydrogen-bonding motif seen in **3**, linked *via* the chiral C4 centre. Dimers are linked *via* meth­oxy–ketone hydrogen bonding, forming a ribbon that extends parallel to the *c* axis. Only hydrogen atoms involved in intra- and inter­molecular hydrogen bonding are shown. Dotted lines indicate hydrogen-bonding inter­actions. [Symmetry codes: (i) −*x* + 1, −*y* + 2, −*z* + 1; (ii) −*x* + 1, −*y* + 2, −*z*].

Compound **3**, with the naphthyl substituent, does not display the same supra­molecular features. A weaker C—H⋯O inter­action from the chiral centre C4 to the oxygen on the central meth­oxy group, *B* ring, links the mol­ecules into a cyclic dimer. These dimers are associated *via* further C—H⋯O hydrogen bonding (Table 2 ▸) into a ribbon extended approximately parallel to the c axis, see Fig. 11 ▸.

## Database survey   

A search of the CSD, (version 5.41, update of March 2020; Groom *et al.*, 2016 ▸) for a 1-(3,4,5-tri­meth­oxy­phen­yl)azetidin-2-one core yielded only 24 compounds and these are shown in Table 3 ▸. Substituents range from 3-phenyl-4-(3-fluoro-4-meth­oxy­phen­yl) in PUKNUH, 3-[4-(1,3-benzo­thia­zol-2-yl)phen­oxy]-4-(4-nitro­phen­yl) in KIFZIL, 3,3-diphenyl-4-(4-meth­oxy­phen­yl) in OSOWEZ, 3-(2-thien­yl)-4-(3-azido­phen­yl) in REFDOY to 3-phen­oxy-4-(3-hy­droxy-4-meth­oxy­phen­yl) in ZUWXAS. All of these compounds display an intra­molecular hydrogen bond between the trimeth­oxy ring and the lactam ketone, the C—H⋯O distance ranging from 3.0236 (11) Å in REFDOY to 3.19298 (7) Å in XAMMEG. Although this association holds the *A* and *B* rings approximately coplanar, there can be a twist in the *B* ring relative to the *A* ring, as seen above with the torsion angle C2—N1—C5—C10 (compound **1**–**5** numbering) showing wide differences: −26.2501 (13)° in PUKNUH, −0.81417 (3)° in PUKPOD and 26.5142 (12)° in PUKPIT. From Table 1 ▸ it can be seen that the twist in **1**–**5** is most pronounced in **5**, possibly to accommodate the steric requirements of the 4,4′ disubstitution.

A wider search of the database for similar structural motifs to **5** using the basic core, 1,3,4,4-tetra­methyl­azetidin-2-one, disubstituted on the 4 position, yields 75 structures of this type of which 28 are non-bicyclic species. Many of these have mixed substitution in position C4 comprising a methyl and an *R* group: *R* = (phenyl­imino)­ethyl, BAGREI; *R* = (meth­oxy­phen­yl)methyl, DAXKIZ; *R* = acetyl, GADHUO; *R* = phenyl, PADYAU; p = phenyl, PIHVEK, PIVHEK01; *Ru* = phenyl, YUDKEP. Mixed aliphatic and aromatic C4 substitution are also seen (GADJAW). There are also carb­oxy or cyano C4-substituted species (FEKRUK; FOMBOB and FOMBUH; IFOSII; MIMLIE, MIMLOK, MIMROQ; POFWEP; REBKIS; TIVBEH). In all of these compounds, the group bonded to the nitro­gen N1 of the lactam varies, for example from phenyl in BAGREI, 4-methyoxybenzyl in DAXKIZ and FEKRUK, 4-chloro­phenyl in GADHUO, 4-nitro­phenyl in IFOSIL, ethyl in POWMOD, and bis­(tri­methyl­sil­yl)methyl in REBKIS.

However there are eight other structures with identical C4 substituents based on the core 1,3,4,4-tetra­methyl­azetidin-2-one core. The common C4 substituent is a methyl group (JAGLEI, KAHWIA01, NAHZOM, POWMOD and ZOHPAN). However, two feature phenyl C4 substituents AHERUA and RIFYIO, and one features a di­carboxyl­ate, QULNUH. Most feature the less bulky ^*i*^Pr substituent on the lactam N1 (JAGLEI, KAHWIA01, NAZHOM and ZOHPAN) or ethyl (POWMOD) or incorporate a spacer such as phenyl­ethyl, QULNUH, or (meth­oxy­carbon­yl)-2-phenyl­eth­yl) in RIFYIO, reducing the steric requirement on the lactam.

## Synthesis and crystallization   

All of these compounds have been prepared previously and the experimental synthesis described for **1**–**3** uses acid activation with triphosgene with an imine (O’Boyle *et al.*, 2010 ▸, 2011*a* ▸), **4** (O’Boyle, 2010 ▸) uses the Staudinger reaction (reaction of the imine/NEt_3_ with the acid chloride), and **5** (O’Boyle, 2011*b* ▸) uses the reaction of TiCl_4_ and the appropriately substituted benzo­phenone with the substituted acyl chloride. The acid or acyl chloride and imines are as follows:

**1**: 2-(4-Fluoro­phen­yl)acetic acid and *N*-(4-meth­oxy­benzyl­idene)-3,4,5-tri­meth­oxy­benzenamine. White crystalline solid. Yield 7.5%,

**2**: 2-(Furan-2-yl)acetic acid and *N*-(4-meth­oxy­benzyl­idene)-3,4,5-tri­meth­oxy­benzenamine. Brown crystals. Yield 4.9%.

**3**: 2-(Naphthalen-1-yl)acetic acid and *N*-(4-meth­oxy­benzyl­idene)-3,4,5-tri­meth­oxy­benzenamine. Pale-yellow crystalline powder. Yield 6.9%.

**4**: 2-(3,4-upi­meth­oxy­phen­yl)acetyl chloride and *N*-(4-meth­oxy­benzyl­idene)-3,4,5-tri­meth­oxy­benzenamine. White powder. Yield 1.1%.

**5**: Phenyl acetyl chloride and bis-(4-meth­oxy­phen­yl)methanone. White powder. Yield 17%.

The crude product was purified by flash column chromatography over silica gel (eluent: hexa­ne/ethyl acetate gradient). The eluent was evaporated and compounds were recrystallized from ethanol.

## Refinement details   

Crystal data, data collection and structure refinement details are summarized in Table 4 ▸. H atoms bonded to carbon were refined in geometrically calculated positions, with C—H= 1.0 (methine), 0.98 (meth­yl) and 0.95 Å (aromatic), and with *U*_iso_(H) = 1.2*U*_eq_(C) (methine, aromatic) or 1.5*U*_eq_(C) (meth­yl). Compounds **2** and **5** were refined with extinction, 0.0011 (2) and 0.00057 (14) respectively.

## Supplementary Material

Crystal structure: contains datablock(s) 1, 2, 3, 4, 5, global. DOI: 10.1107/S2056989020008555/zl2789sup1.cif

Structure factors: contains datablock(s) 1. DOI: 10.1107/S2056989020008555/zl27891sup2.hkl

Structure factors: contains datablock(s) 2. DOI: 10.1107/S2056989020008555/zl27892sup3.hkl

Structure factors: contains datablock(s) 3. DOI: 10.1107/S2056989020008555/zl27893sup4.hkl

Structure factors: contains datablock(s) 4. DOI: 10.1107/S2056989020008555/zl27894sup5.hkl

Structure factors: contains datablock(s) 5. DOI: 10.1107/S2056989020008555/zl27895sup6.hkl

Supporting information file. DOI: 10.1107/S2056989020008555/zl27891sup7.cml

Supporting information file. DOI: 10.1107/S2056989020008555/zl27892sup8.cml

Supporting information file. DOI: 10.1107/S2056989020008555/zl27893sup9.cml

Supporting information file. DOI: 10.1107/S2056989020008555/zl27894sup10.cml

Supporting information file. DOI: 10.1107/S2056989020008555/zl27895sup11.cml

Overlay of 1 and PUKNUH with overlay centres N1 C3 and O17/O3 and all oxygen atoms labelled, showing the difference in the B ring orientation. The para-methoxy substituent is also flipped. The C and D ring orientations are similar. DOI: 10.1107/S2056989020008555/zl2789sup12.tif

Overlay with inversion of each unique molecule in the asymmetric unit of 2, showing the difference in the D ring orientations and para-methoxy groups as well as the rotation of the D ring (furan). DOI: 10.1107/S2056989020008555/zl2789sup13.tif

Overlay with inversion of each unique molecule in the asymmetric unit of 5, showing the difference in the B ring orientations, and C' ring para-methoxy group differences. DOI: 10.1107/S2056989020008555/zl2789sup14.tif

CCDC references: 2012288, 2012287, 2012286, 2012285, 2012284

Additional supporting information:  crystallographic information; 3D view; checkCIF report

## Figures and Tables

**Table 1:** Extra geometric parameters (°) for **1**–**5**

	**1**	**2**		**3**	**4**	**5**	
*A*|*B* plane normals	13.17 (9)	7.64 (13)	2.62 (13)	12.20 (19)	6.56 (13)	17.08 (9)	15.89 (8)
*A*|*C* plane normals	84.68 (6)	83.59 (9)	83.84 (9)	89.56 (8)	89.15 (6)	89.23 (6) 67.36 (6)	85.80 (6) 63.60 (6)
*A*|*D* plane normals	88.41 (7)	85.72 (9)	63.03 (8)	53.23 (10)	86.00 (7)	69.26 (5)	73.37 (5)
Chirality C3	*S*	*S*	*R*	*S*	*S*	*R*	*S*
Chirality C4	*R*	*S*	*R*	*R*	*R*	–	–
H3—C3—C4—C18^†^	6.26	−7.27	10.42	4.38	13.08	−0.98	−1.82
H4—C4—C3—C26^†^	8.22	−8.69	12.21	4.96	13.76		
C2—N1—C5—C10	−4.3 (3)	−9.4 (3)	3.8 (4)	10.3 (4)	−5.5 (2)	20.2 (2)	−21.8 (2)

† Riding hydrogen atom used in torsion angle, no associated s.u.

**Table 2:** Hydrogen-bond geometry (Å, °) for **1**–**5**

*D*—H⋯*A*	*D*—H	H⋯*A*	*D*⋯*A*	*D*—H⋯*A*
**1**				
C10—H10⋯O17	0.95	2.46	3.091 (2)	123
C12—H12*C*⋯O11^i^	0.98	2.48	3.243 (2)	135
Symmetry codes: (i) −*x* + 1, −*y* + 1, −*z* + 2.				
				
**2**				
C10—H10⋯O17	0.95	2.50	3.103 (3)	122
C12—H12*C*⋯O11^i^	0.98	2.48	3.171 (3)	127
C12—H12*C*⋯·O13^i^	0.98	2.55	3.482 (3)	159
C22—H22⋯O24*A* ^ii^	0.95	2.39	3.143 (3)	136
C10*A*—H10*A*⋯O17*A*	0.95	2.53	3.144 (3)	123
C22*A*—H22*A*⋯O24^iii^	0.95	2.40	3.274 (3)	153
Symmetry codes: (i) −*x* + 1, −*y*, −*z* + 1; (ii) *x* + 1, *y* − 1, *z*; (iii) *x* − 1, *y*, *z*.				
				
**3**				
C4—H4⋯O13^i^	1.00	2.41	3.396 (3)	171
C10—H10⋯O17	0.95	2.50	3.105 (3)	122
C16—H16*B*⋯O17^ii^	0.98	2.53	3.387 (3)	146
C27—H27⋯O17	0.95	2.46	3.156 (3)	131
Symmetry codes: (i) −*x* + 1, −*y* + 2, −*z* + 1; (ii) −*x* + 1, −*y* + 2, −*z*.				
				
**4**				
C10—H10⋯O17	0.95	2.46	3.088 (2)	124
C33—H33*A*⋯O13^i^	0.98	2.44	3.411 (2)	170
C33—H33*A*⋯O15^i^	0.98	2.56	3.228 (2)	125
Symmetry code: (i) −*x* + 1, −*y* + 1, −*z* + 1.				
				
**5**				
C10—H10⋯O17	0.95	2.53	3.119 (2)	120
C19—H19⋯O17^i^	0.95	2.63	3.236 (2)	122
C33—H33*A*⋯O32^ii^	0.98	2.56	3.211 (2)	124
C33—H33*B*⋯O17*A*	0.98	2.37	3.259 (2)	151
C10*A*—H10*A*⋯O17*A*	0.95	2.53	3.116 (2)	120
C33*A*—H33*E*⋯O32*A* ^iii^	0.98	2.55	3.192 (2)	123
Symmetry codes: (i) −*x* + 1, −*y*, 1 − *z*; (ii) −*x* + 1, −*y*, −*z*; (iii) −*x* + 1, −*y* + 1, −*z* + 1.				

**Table 3:** Database Survey

CSD Refcode	Reference	CSD Refcode	Reference
PUKNUH	Malebari *et al.* (2020 ▸)	AHERUA	Usman *et al.* (2002 ▸)
PUKPAP	Malebari *et al.* (2020 ▸)	BAGREI	Wang *et al.* (2011 ▸)
PUKPET	Malebari *et al.* (2020 ▸)	DAXKIZ	Piens *et al.* (2017 ▸)
PUKPIX	Malebari *et al.* (2020 ▸)	FEKRUK	Yoshimura *et al.* (2012 ▸)
PUKPOD	Malebari *et al.* (2020 ▸)	FOMBOB, FOMBUH	Chen *et al.* (2019*b* ▸)
KAKTIB	O’Boyle *et al.* (2010 ▸)	GADHUO	Alcaide *et al.* (1987 ▸)
KIFZIL	Alborz *et al.* (2018 ▸)	GADJAW	Alcaide *et al.* (1987 ▸)
NARWIO	O’Boyle *et al.* (2011*a* ▸)	IFOSII	Gao *et al.* (2018 ▸)
OSOWAV	O’Boyle *et al.* (2011*b* ▸)	JAGLEI	Sekine *et al.* (1989 ▸)
OSOWEZ	O’Boyle *et al.* (2011*b* ▸)	KAHWIA01	Natarajan *et al.* (2005 ▸)
OSOWID	O’Boyle *et al.* (2011*b* ▸)	MIMLIE, MIMLOK, MIMROQ	Cheng & Cheng (2007 ▸)
REFDOY	Fu *et al.* (2017 ▸)	NAZHOM	Natarajan *et al.* (2005 ▸)
XALYAN	Malebari *et al.* (2017 ▸)	PADYAU	Kohmoto *et al.* (1992 ▸)
XAMLUV	Malebari *et al.* (2017 ▸)	PIHVEK	Martinez-Cuezva *et al.* (2018 ▸)
XAMMAC	Malebari *et al.* (2017 ▸)	PIVHEK01	Martinez-Cuezva *et al.* (2019 ▸)
XAMMEG	Malebari *et al.* (2017 ▸)	POFWEP	Chen *et al.* (2019*a* ▸)
ZUWVUK	Greene *et al.* (2016 ▸)	POWMOD	Toda *et al.* (1997 ▸)
ZUWWAR	Greene *et al.* (2016 ▸)	QULNUH	Minato *et al.* (2009 ▸)
ZUWWEV	Greene *et al.* (2016 ▸)	REBKIS	Palomo *et al.* (1997 ▸)
ZUWWIZ	Greene *et al.* (2016 ▸)	RIFYIO	Zaragoza & Zahn (1995 ▸)
ZUWWOF	Greene *et al.* (2016 ▸)	TIVBEH	Mandler *et al.* (2014 ▸)
ZUWWUL	Greene *et al.* (2016 ▸)	YUDKEP	Bandyopadhyay (2015 ▸)
ZUWXAS	Greene *et al.* (2016 ▸)	ZOHPAN	Hashizume *et al.* (1996 ▸)
ZUWXEW	Greene *et al.* (2016 ▸)		

**Table 4:** Experimental details

	**1**	**2**	**3**	**4**	**5**
Crystal data					
Chemical formula	C_25_H_24_FNO_5_	C_23_H_23_NO_6_	C_29_H_27_NO_5_	C_27_H_29_NO_7_	C_32_H_31_NO_6_
*M*_r_	437.45	409.42	469.51	479.51	525.58
Crystal system, space group	Orthorhombic, *P* *b* *c* *a*	Monoclinic, *P*2_1_/*c*	Triclinic, *P* 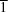	Monoclinic, *P*2_1_/*c*	Triclinic, *P* 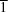
Temperature (K)	100	100	100	100	100
*a*, *b*, *c* (Å)	18.6879 (16), 9.4736 (8), 24.283 (2)	19.794 (2), 9.1396 (9), 23.161 (2)	10.4633 (6), 11.3180 (6), 11.6008 (6)	8.858 (2), 22.769 (5), 12.822 (2)	11.5720 (3), 12.3994 (3), 19.9358 (6)
α, β, γ (°)	90, 90, 90	90, 101.0705 (18), 90	104.628 (3), 99.056 (4), 112.929 (3)	90, 109.839 (6), 90	83.779 (1), 85.748 (1), 71.559 (1)
*V* (Å^3^)	4299.1 (6)	4112.0 (7)	1172.74 (12)	2432.4 (9)	2695.23 (13)
*Z*	8	8	2	4	4
Radiation type	Mo *K*α	Mo *K*α	Cu *K*α	Mo *K*α	Cu *K*α
μ (mm^−1^)	0.10	0.10	0.74	0.10	0.73
Crystal size (mm)	0.38 × 0.12 × 0.06	0.42 × 0.3 × 0.04	0.27 × 0.15 × 0.04	0.41 × 0.24 × 0.12	0.26 × 0.15 × 0.04

Data collection					
Diffractometer	Bruker APEXII Kappa Duo	Bruker APEXII Kappa Duo	Bruker APEXII Kappa Duo	Bruker D8 Quest ECO	Bruker APEXII Kappa Duo
Absorption correction	Multi-scan (*SADABS*; Bruker, 2016 ▸)	Multi-scan (*SADABS*; Bruker, 2016 ▸)	Multi-scan (*SADABS*; Bruker, 2016 ▸)	Multi-scan (*SADABS*; Bruker, 2016 ▸)	Multi-scan (*SADABS*; Bruker, 2016 ▸)
*T*_min_, *T*_max_	0.689, 0.746	0.660, 0.745	0.598, 0.753	0.701, 0.746	0.695, 0.753
No. of measured, independent and observed [*I* > 2σ(*I*)] reflections	36881, 6857, 4084	104383, 8072, 5950	18268, 4385, 3453	27592, 5620, 4062	37871, 9870, 8278
*R*_int_	0.089	0.091	0.065	0.058	0.040
(sin θ/λ)_max_ (Å^−1^)	0.725	0.619	0.610	0.651	0.606

Refinement					
*R*[*F* ^2^ > 2σ(*F* ^2^)], *wR*(*F* ^2^), *S*	0.054, 0.123, 1.01	0.056, 0.112, 1.08	0.060, 0.188, 1.09	0.045, 0.106, 1.03	0.045, 0.137, 1.09
No. of reflections	6857	8072	4385	5620	9870
No. of parameters	293	550	320	322	714
H-atom treatment	H-atom parameters constrained	H-atom parameters constrained	H-atom parameters constrained	H-atom parameters constrained	H-atom parameters constrained
Δρ_max_, Δρ_min_ (e Å^−3^)	0.30, −0.29	0.25, −0.25	0.32, −0.37	0.29, −0.24	0.24, −0.24

Computer programs: *APEX3* (Bruker, 2017 ▸), *SAINT* (Bruker, 2016 ▸), *SHELXT* (Sheldrick, 2015*a* ▸), *SHELXL* (Sheldrick, 2015*b* ▸) and *OLEX2* (Dolomanov *et al.*, 2009 ▸).

## supplementary crystallographic information

### 3-(4-Fluorophenyl)-4-(4-methoxyphenyl)-1-(3,4,5-trimethoxyphenyl)azetidin-2-one (1) Crystal data

**Table:**

C_25_H_24_FNO_5_	*D*_x_ = 1.352 Mg m^−^^3^
*M**_r_* = 437.45	Mo *K*α radiation, λ = 0.71073 Å
Orthorhombic, *P**b**c**a*	Cell parameters from 3522 reflections
*a* = 18.6879 (16) Å	θ = 3.1–31.0°
*b* = 9.4736 (8) Å	µ = 0.10 mm^−^^1^
*c* = 24.283 (2) Å	*T* = 100 K
*V* = 4299.1 (6) Å^3^	Block, clear colourless
*Z* = 8	0.38 × 0.12 × 0.06 mm
*F*(000) = 1840	

### 3-(4-Fluorophenyl)-4-(4-methoxyphenyl)-1-(3,4,5-trimethoxyphenyl)azetidin-2-one (1) Data collection

**Table:**

Bruker APEXII Kappa Duo diffractometer	6857 independent reflections
Radiation source: microfocus sealed X-ray tube, Incoatec Iµs	4084 reflections with *I* > 2σ(*I*)
Mirror optics monochromator	*R*_int_ = 0.089
Detector resolution: 8.33 pixels mm^-1^	θ_max_ = 31.0°, θ_min_ = 1.7°
ω and φ scans	*h* = −26→21
Absorption correction: multi-scan (SADABS; Bruker, 2016)	*k* = −13→13
*T*_min_ = 0.689, *T*_max_ = 0.746	*l* = −35→35
36881 measured reflections	

### 3-(4-Fluorophenyl)-4-(4-methoxyphenyl)-1-(3,4,5-trimethoxyphenyl)azetidin-2-one (1) Refinement

**Table:**

Refinement on *F*^2^	Primary atom site location: dual
Least-squares matrix: full	Hydrogen site location: inferred from neighbouring sites
*R*[*F*^2^ > 2σ(*F*^2^)] = 0.054	H-atom parameters constrained
*wR*(*F*^2^) = 0.123	*w* = 1/[σ^2^(*F*_o_^2^) + (0.0465*P*)^2^ + 1.1677*P*] where *P* = (*F*_o_^2^ + 2*F*_c_^2^)/3
*S* = 1.01	(Δ/σ)_max_ = 0.001
6857 reflections	Δρ_max_ = 0.30 e Å^−^^3^
293 parameters	Δρ_min_ = −0.29 e Å^−^^3^
0 restraints	

### 3-(4-Fluorophenyl)-4-(4-methoxyphenyl)-1-(3,4,5-trimethoxyphenyl)azetidin-2-one (1) Special details

**Table:**

Geometry. All esds (except the esd in the dihedral angle between two l.s. planes) are estimated using the full covariance matrix. The cell esds are taken into account individually in the estimation of esds in distances, angles and torsion angles; correlations between esds in cell parameters are only used when they are defined by crystal symmetry. An approximate (isotropic) treatment of cell esds is used for estimating esds involving l.s. planes.

### 3-(4-Fluorophenyl)-4-(4-methoxyphenyl)-1-(3,4,5-trimethoxyphenyl)azetidin-2-one (1) Fractional atomic coordinates and isotropic or equivalent isotropic displacement parameters (Å^2^)

**Table:**

	*x*	*y*	*z*	*U*_iso_*/*U*_eq_	
C2	0.47067 (9)	0.32435 (16)	0.69423 (6)	0.0167 (3)	
C3	0.54877 (9)	0.34631 (16)	0.67672 (6)	0.0168 (3)	
H3	0.574487	0.253748	0.676899	0.020*	
C4	0.55863 (9)	0.42137 (16)	0.73381 (6)	0.0161 (3)	
H4	0.567673	0.524850	0.729603	0.019*	
C5	0.44073 (9)	0.39639 (16)	0.79263 (6)	0.0157 (3)	
C6	0.47056 (9)	0.46256 (16)	0.83827 (6)	0.0168 (3)	
H6	0.516056	0.506887	0.835923	0.020*	
C7	0.43227 (9)	0.46254 (16)	0.88764 (6)	0.0183 (3)	
C8	0.36475 (9)	0.40188 (17)	0.89083 (6)	0.0192 (3)	
C9	0.33578 (9)	0.33638 (16)	0.84420 (7)	0.0182 (3)	
C10	0.37356 (9)	0.33370 (16)	0.79465 (7)	0.0176 (3)	
H10	0.353756	0.289893	0.762935	0.021*	
C12	0.53206 (11)	0.5642 (2)	0.93492 (7)	0.0284 (4)	
H12A	0.536811	0.643948	0.909487	0.043*	
H12B	0.563192	0.486916	0.922824	0.043*	
H12C	0.545983	0.593991	0.972046	0.043*	
C14	0.28311 (14)	0.5105 (2)	0.95034 (10)	0.0469 (6)	
H14A	0.249823	0.520605	0.919411	0.070*	
H14B	0.311537	0.596888	0.954079	0.070*	
H14C	0.256060	0.494181	0.984326	0.070*	
C16	0.24554 (10)	0.18401 (19)	0.80868 (7)	0.0253 (4)	
H16A	0.239028	0.237693	0.774552	0.038*	
H16B	0.199815	0.141896	0.819672	0.038*	
H16C	0.280870	0.109139	0.802677	0.038*	
C18	0.60855 (9)	0.35478 (16)	0.77494 (6)	0.0153 (3)	
C19	0.65897 (9)	0.43381 (16)	0.80287 (7)	0.0192 (3)	
H19	0.664258	0.531044	0.794202	0.023*	
C20	0.70236 (9)	0.37431 (17)	0.84352 (7)	0.0200 (3)	
H20	0.737438	0.429973	0.861693	0.024*	
C21	0.69374 (9)	0.23316 (17)	0.85708 (7)	0.0181 (3)	
C22	0.64337 (9)	0.15142 (17)	0.82885 (7)	0.0203 (4)	
H22	0.637915	0.054211	0.837481	0.024*	
C23	0.60168 (9)	0.21209 (16)	0.78851 (7)	0.0183 (3)	
H23	0.567589	0.155813	0.769535	0.022*	
C25	0.78712 (10)	0.24316 (19)	0.92421 (7)	0.0245 (4)	
H25A	0.822515	0.272413	0.896703	0.037*	
H25B	0.810239	0.183185	0.951859	0.037*	
H25C	0.767008	0.326872	0.942168	0.037*	
C26	0.56251 (9)	0.42157 (17)	0.62316 (6)	0.0172 (3)	
C27	0.59598 (10)	0.34916 (19)	0.58044 (7)	0.0248 (4)	
H27	0.612957	0.255969	0.586601	0.030*	
C28	0.60505 (11)	0.4109 (2)	0.52881 (7)	0.0290 (4)	
H28	0.627489	0.360816	0.499640	0.035*	
C29	0.58074 (10)	0.54535 (19)	0.52142 (7)	0.0230 (4)	
C30	0.54810 (11)	0.62143 (18)	0.56226 (7)	0.0249 (4)	
H30	0.532024	0.715082	0.555723	0.030*	
C31	0.53903 (10)	0.55855 (17)	0.61345 (7)	0.0222 (4)	
H31	0.516475	0.609864	0.642231	0.027*	
F1	0.58878 (6)	0.60640 (12)	0.47102 (4)	0.0335 (3)	
N1	0.48152 (7)	0.39025 (13)	0.74384 (5)	0.0150 (3)	
O11	0.45909 (7)	0.51679 (12)	0.93550 (5)	0.0235 (3)	
O13	0.32920 (7)	0.39465 (13)	0.94054 (5)	0.0252 (3)	
O15	0.27020 (6)	0.27641 (12)	0.85124 (5)	0.0243 (3)	
O17	0.41799 (7)	0.26893 (12)	0.67469 (5)	0.0217 (3)	
O24	0.73096 (7)	0.16568 (12)	0.89773 (5)	0.0237 (3)	

### 3-(4-Fluorophenyl)-4-(4-methoxyphenyl)-1-(3,4,5-trimethoxyphenyl)azetidin-2-one (1) Atomic displacement parameters (Å^2^)

**Table:**

	*U*^11^	*U*^22^	*U*^33^	*U*^12^	*U*^13^	*U*^23^
C2	0.0231 (9)	0.0135 (7)	0.0133 (7)	0.0008 (7)	−0.0001 (6)	0.0001 (6)
C3	0.0209 (9)	0.0144 (7)	0.0151 (7)	−0.0005 (6)	0.0017 (6)	−0.0012 (6)
C4	0.0190 (9)	0.0150 (7)	0.0143 (7)	−0.0002 (6)	0.0003 (6)	0.0000 (6)
C5	0.0210 (9)	0.0127 (7)	0.0134 (7)	0.0027 (6)	0.0016 (6)	0.0016 (5)
C6	0.0219 (9)	0.0137 (7)	0.0149 (7)	−0.0016 (6)	0.0008 (6)	0.0017 (6)
C7	0.0285 (10)	0.0135 (7)	0.0129 (7)	0.0017 (7)	−0.0021 (6)	0.0005 (6)
C8	0.0244 (10)	0.0193 (8)	0.0139 (7)	0.0031 (7)	0.0040 (6)	0.0024 (6)
C9	0.0197 (9)	0.0160 (7)	0.0190 (8)	0.0019 (7)	0.0012 (7)	0.0021 (6)
C10	0.0206 (9)	0.0161 (7)	0.0161 (7)	0.0017 (7)	−0.0010 (6)	−0.0008 (6)
C12	0.0356 (12)	0.0353 (10)	0.0144 (8)	−0.0139 (9)	−0.0030 (7)	0.0014 (7)
C14	0.0583 (16)	0.0337 (11)	0.0487 (14)	0.0113 (11)	0.0362 (12)	0.0062 (10)
C16	0.0219 (10)	0.0296 (9)	0.0246 (9)	−0.0051 (8)	−0.0005 (7)	−0.0007 (7)
C18	0.0164 (8)	0.0149 (7)	0.0146 (7)	−0.0008 (6)	0.0019 (6)	−0.0009 (6)
C19	0.0224 (9)	0.0136 (7)	0.0217 (8)	−0.0011 (7)	−0.0001 (7)	−0.0002 (6)
C20	0.0185 (9)	0.0196 (8)	0.0217 (8)	−0.0018 (7)	−0.0019 (7)	−0.0039 (6)
C21	0.0183 (9)	0.0182 (8)	0.0178 (7)	0.0028 (7)	−0.0013 (6)	−0.0017 (6)
C22	0.0239 (10)	0.0132 (7)	0.0238 (8)	0.0001 (7)	−0.0022 (7)	0.0009 (6)
C23	0.0196 (9)	0.0156 (7)	0.0197 (8)	−0.0032 (7)	−0.0022 (7)	−0.0016 (6)
C25	0.0220 (9)	0.0302 (9)	0.0212 (8)	0.0001 (8)	−0.0061 (7)	−0.0016 (7)
C26	0.0171 (9)	0.0188 (7)	0.0156 (7)	−0.0030 (7)	−0.0003 (6)	0.0004 (6)
C27	0.0307 (11)	0.0235 (8)	0.0202 (8)	0.0047 (8)	0.0061 (7)	0.0013 (7)
C28	0.0327 (11)	0.0356 (10)	0.0189 (8)	0.0024 (9)	0.0098 (8)	0.0019 (7)
C29	0.0246 (10)	0.0286 (9)	0.0157 (8)	−0.0094 (8)	−0.0011 (7)	0.0058 (7)
C30	0.0363 (12)	0.0182 (8)	0.0204 (8)	−0.0045 (8)	−0.0058 (8)	0.0025 (6)
C31	0.0299 (10)	0.0194 (8)	0.0172 (8)	−0.0001 (7)	0.0006 (7)	−0.0033 (6)
F1	0.0410 (7)	0.0405 (6)	0.0190 (5)	−0.0099 (5)	0.0026 (5)	0.0107 (5)
N1	0.0162 (7)	0.0160 (6)	0.0129 (6)	−0.0001 (5)	0.0001 (5)	−0.0011 (5)
O11	0.0323 (7)	0.0257 (6)	0.0126 (5)	−0.0068 (6)	0.0003 (5)	−0.0012 (4)
O13	0.0322 (8)	0.0275 (6)	0.0160 (6)	0.0033 (6)	0.0087 (5)	0.0018 (5)
O15	0.0206 (7)	0.0290 (6)	0.0235 (6)	−0.0041 (5)	0.0043 (5)	−0.0026 (5)
O17	0.0236 (7)	0.0242 (6)	0.0173 (6)	−0.0049 (5)	−0.0002 (5)	−0.0040 (5)
O24	0.0255 (7)	0.0205 (6)	0.0252 (6)	0.0005 (5)	−0.0091 (5)	0.0008 (5)

### 3-(4-Fluorophenyl)-4-(4-methoxyphenyl)-1-(3,4,5-trimethoxyphenyl)azetidin-2-one (1) Geometric parameters (Å, º)

**Table:**

C2—C3	1.534 (2)	C16—H16B	0.9800
C2—N1	1.3719 (19)	C16—H16C	0.9800
C2—O17	1.213 (2)	C16—O15	1.431 (2)
C3—H3	1.0000	C18—C19	1.381 (2)
C3—C4	1.569 (2)	C18—C23	1.397 (2)
C3—C26	1.505 (2)	C19—H19	0.9500
C4—H4	1.0000	C19—C20	1.396 (2)
C4—C18	1.505 (2)	C20—H20	0.9500
C4—N1	1.491 (2)	C20—C21	1.387 (2)
C5—C6	1.390 (2)	C21—C22	1.399 (2)
C5—C10	1.390 (2)	C21—O24	1.366 (2)
C5—N1	1.410 (2)	C22—H22	0.9500
C6—H6	0.9500	C22—C23	1.377 (2)
C6—C7	1.396 (2)	C23—H23	0.9500
C7—C8	1.389 (2)	C25—H25A	0.9800
C7—O11	1.3659 (19)	C25—H25B	0.9800
C8—C9	1.400 (2)	C25—H25C	0.9800
C8—O13	1.3796 (19)	C25—O24	1.433 (2)
C9—C10	1.395 (2)	C26—C27	1.392 (2)
C9—O15	1.362 (2)	C26—C31	1.390 (2)
C10—H10	0.9500	C27—H27	0.9500
C12—H12A	0.9800	C27—C28	1.394 (2)
C12—H12B	0.9800	C28—H28	0.9500
C12—H12C	0.9800	C28—C29	1.364 (3)
C12—O11	1.436 (2)	C29—C30	1.369 (3)
C14—H14A	0.9800	C29—F1	1.3619 (19)
C14—H14B	0.9800	C30—H30	0.9500
C14—H14C	0.9800	C30—C31	1.389 (2)
C14—O13	1.415 (2)	C31—H31	0.9500
C16—H16A	0.9800		
			
N1—C2—C3	92.36 (12)	O15—C16—H16C	109.5
O17—C2—C3	136.27 (14)	C19—C18—C4	121.42 (13)
O17—C2—N1	131.35 (15)	C19—C18—C23	118.12 (15)
C2—C3—H3	109.7	C23—C18—C4	120.33 (14)
C2—C3—C4	85.89 (11)	C18—C19—H19	119.2
C4—C3—H3	109.7	C18—C19—C20	121.62 (15)
C26—C3—C2	117.77 (14)	C20—C19—H19	119.2
C26—C3—H3	109.7	C19—C20—H20	120.3
C26—C3—C4	121.92 (13)	C21—C20—C19	119.33 (15)
C3—C4—H4	112.0	C21—C20—H20	120.3
C18—C4—C3	117.98 (13)	C20—C21—C22	119.71 (15)
C18—C4—H4	112.0	O24—C21—C20	124.32 (15)
N1—C4—C3	86.63 (11)	O24—C21—C22	115.96 (14)
N1—C4—H4	112.0	C21—C22—H22	120.1
N1—C4—C18	114.06 (13)	C23—C22—C21	119.90 (15)
C6—C5—N1	118.15 (15)	C23—C22—H22	120.1
C10—C5—C6	121.79 (14)	C18—C23—H23	119.4
C10—C5—N1	120.03 (14)	C22—C23—C18	121.29 (15)
C5—C6—H6	120.7	C22—C23—H23	119.4
C5—C6—C7	118.62 (16)	H25A—C25—H25B	109.5
C7—C6—H6	120.7	H25A—C25—H25C	109.5
C8—C7—C6	120.90 (15)	H25B—C25—H25C	109.5
O11—C7—C6	122.84 (16)	O24—C25—H25A	109.5
O11—C7—C8	116.22 (14)	O24—C25—H25B	109.5
C7—C8—C9	119.32 (15)	O24—C25—H25C	109.5
O13—C8—C7	120.45 (15)	C27—C26—C3	119.15 (15)
O13—C8—C9	119.97 (15)	C31—C26—C3	122.34 (15)
C10—C9—C8	120.65 (16)	C31—C26—C27	118.40 (15)
O15—C9—C8	115.56 (14)	C26—C27—H27	119.4
O15—C9—C10	123.79 (15)	C26—C27—C28	121.21 (16)
C5—C10—C9	118.68 (15)	C28—C27—H27	119.4
C5—C10—H10	120.7	C27—C28—H28	121.0
C9—C10—H10	120.7	C29—C28—C27	118.00 (17)
H12A—C12—H12B	109.5	C29—C28—H28	121.0
H12A—C12—H12C	109.5	C28—C29—C30	122.99 (16)
H12B—C12—H12C	109.5	F1—C29—C28	118.56 (16)
O11—C12—H12A	109.5	F1—C29—C30	118.45 (16)
O11—C12—H12B	109.5	C29—C30—H30	120.8
O11—C12—H12C	109.5	C29—C30—C31	118.47 (16)
H14A—C14—H14B	109.5	C31—C30—H30	120.8
H14A—C14—H14C	109.5	C26—C31—H31	119.5
H14B—C14—H14C	109.5	C30—C31—C26	120.91 (16)
O13—C14—H14A	109.5	C30—C31—H31	119.5
O13—C14—H14B	109.5	C2—N1—C4	95.12 (12)
O13—C14—H14C	109.5	C2—N1—C5	132.59 (14)
H16A—C16—H16B	109.5	C5—N1—C4	130.67 (13)
H16A—C16—H16C	109.5	C7—O11—C12	117.25 (13)
H16B—C16—H16C	109.5	C8—O13—C14	113.68 (14)
O15—C16—H16A	109.5	C9—O15—C16	117.04 (13)
O15—C16—H16B	109.5	C21—O24—C25	117.21 (13)
			
C2—C3—C4—C18	115.73 (14)	C18—C4—N1—C5	47.2 (2)
C2—C3—C4—N1	0.19 (11)	C18—C19—C20—C21	−1.4 (3)
C2—C3—C26—C27	−116.12 (17)	C19—C18—C23—C22	0.5 (2)
C2—C3—C26—C31	59.8 (2)	C19—C20—C21—C22	1.9 (3)
C3—C2—N1—C4	0.22 (12)	C19—C20—C21—O24	−177.04 (16)
C3—C2—N1—C5	−165.98 (16)	C20—C21—C22—C23	−1.2 (3)
C3—C4—C18—C19	133.10 (16)	C20—C21—O24—C25	−6.2 (2)
C3—C4—C18—C23	−51.2 (2)	C21—C22—C23—C18	0.0 (3)
C3—C4—N1—C2	−0.21 (12)	C22—C21—O24—C25	174.84 (15)
C3—C4—N1—C5	166.40 (15)	C23—C18—C19—C20	0.1 (2)
C3—C26—C27—C28	175.17 (17)	C26—C3—C4—C18	−123.90 (17)
C3—C26—C31—C30	−175.38 (16)	C26—C3—C4—N1	120.56 (15)
C4—C3—C26—C27	140.43 (17)	C26—C27—C28—C29	0.6 (3)
C4—C3—C26—C31	−43.6 (2)	C27—C26—C31—C30	0.6 (3)
C4—C18—C19—C20	175.95 (15)	C27—C28—C29—C30	0.0 (3)
C4—C18—C23—C22	−175.32 (15)	C27—C28—C29—F1	−179.49 (16)
C5—C6—C7—C8	−2.1 (2)	C28—C29—C30—C31	−0.3 (3)
C5—C6—C7—O11	175.69 (14)	C29—C30—C31—C26	0.0 (3)
C6—C5—C10—C9	−0.7 (2)	C31—C26—C27—C28	−0.9 (3)
C6—C5—N1—C2	173.83 (15)	F1—C29—C30—C31	179.16 (16)
C6—C5—N1—C4	12.1 (2)	N1—C2—C3—C4	−0.20 (11)
C6—C7—C8—C9	1.9 (2)	N1—C2—C3—C26	−124.35 (14)
C6—C7—C8—O13	175.99 (14)	N1—C4—C18—C19	−127.44 (16)
C6—C7—O11—C12	−5.4 (2)	N1—C4—C18—C23	48.3 (2)
C7—C8—C9—C10	−1.0 (2)	N1—C5—C6—C7	−176.57 (14)
C7—C8—C9—O15	178.35 (14)	N1—C5—C10—C9	177.32 (14)
C7—C8—O13—C14	92.8 (2)	O11—C7—C8—C9	−176.06 (14)
C8—C7—O11—C12	172.48 (14)	O11—C7—C8—O13	−1.9 (2)
C8—C9—C10—C5	0.5 (2)	O13—C8—C9—C10	−175.20 (14)
C8—C9—O15—C16	−167.28 (15)	O13—C8—C9—O15	4.2 (2)
C9—C8—O13—C14	−93.2 (2)	O15—C9—C10—C5	−178.86 (14)
C10—C5—C6—C7	1.5 (2)	O17—C2—C3—C4	−178.65 (19)
C10—C5—N1—C2	−4.3 (3)	O17—C2—C3—C26	57.2 (3)
C10—C5—N1—C4	−166.05 (14)	O17—C2—N1—C4	178.78 (17)
C10—C9—O15—C16	12.1 (2)	O17—C2—N1—C5	12.6 (3)
C18—C4—N1—C2	−119.45 (13)	O24—C21—C22—C23	177.78 (15)

### 3-(4-Fluorophenyl)-4-(4-methoxyphenyl)-1-(3,4,5-trimethoxyphenyl)azetidin-2-one (1) Hydrogen-bond geometry (Å, º)

**Table:**

*D*—H···*A*	*D*—H	H···*A*	*D*···*A*	*D*—H···*A*
C10—H10···O17	0.95	2.46	3.091 (2)	123
C12—H12*C*···O11^i^	0.98	2.48	3.243 (2)	135

Symmetry code: (i) −*x*+1, −*y*+1, −*z*+2.

### 3-(Furan-2-yl)-4-(4-methoxyphenyl)-1-(3,4,5-trimethoxyphenyl)azetidin-2-one (2) Crystal data

**Table:**

C_23_H_23_NO_6_	*F*(000) = 1728
*M**_r_* = 409.42	*D*_x_ = 1.323 Mg m^−^^3^
Monoclinic, *P*2_1_/*c*	Mo *K*α radiation, λ = 0.71073 Å
*a* = 19.794 (2) Å	Cell parameters from 9712 reflections
*b* = 9.1396 (9) Å	θ = 2.2–26.1°
*c* = 23.161 (2) Å	µ = 0.10 mm^−^^1^
β = 101.0705 (18)°	*T* = 100 K
*V* = 4112.0 (7) Å^3^	Block, colourless
*Z* = 8	0.42 × 0.3 × 0.04 mm

### 3-(Furan-2-yl)-4-(4-methoxyphenyl)-1-(3,4,5-trimethoxyphenyl)azetidin-2-one (2) Data collection

**Table:**

Bruker APEXII Kappa Duo diffractometer	8072 independent reflections
Radiation source: standard sealed X-ray tube, Siemens, KFF Mo 2K -90 C	5950 reflections with *I* > 2σ(*I*)
Graphite monochromator	*R*_int_ = 0.091
Detector resolution: 8.33 pixels mm^-1^	θ_max_ = 26.1°, θ_min_ = 1.8°
ω and φ scans	*h* = −24→24
Absorption correction: multi-scan (SADABS; Bruker, 2016)	*k* = −11→11
*T*_min_ = 0.660, *T*_max_ = 0.745	*l* = −28→28
104383 measured reflections	

### 3-(Furan-2-yl)-4-(4-methoxyphenyl)-1-(3,4,5-trimethoxyphenyl)azetidin-2-one (2) Refinement

**Table:**

Refinement on *F*^2^	Hydrogen site location: inferred from neighbouring sites
Least-squares matrix: full	H-atom parameters constrained
*R*[*F*^2^ > 2σ(*F*^2^)] = 0.056	*w* = 1/[σ^2^(*F*_o_^2^) + (0.0339*P*)^2^ + 3.2926*P*] where *P* = (*F*_o_^2^ + 2*F*_c_^2^)/3
*wR*(*F*^2^) = 0.112	(Δ/σ)_max_ < 0.001
*S* = 1.08	Δρ_max_ = 0.25 e Å^−^^3^
8072 reflections	Δρ_min_ = −0.25 e Å^−^^3^
550 parameters	Extinction correction: SHELXL (Sheldrick, 2015b), Fc^*^=kFc[1+0.001xFc^2^λ^3^/sin(2θ)]^-1/4^
0 restraints	Extinction coefficient: 0.00110 (18)

### 3-(Furan-2-yl)-4-(4-methoxyphenyl)-1-(3,4,5-trimethoxyphenyl)azetidin-2-one (2) Special details

**Table:**

Geometry. All esds (except the esd in the dihedral angle between two l.s. planes) are estimated using the full covariance matrix. The cell esds are taken into account individually in the estimation of esds in distances, angles and torsion angles; correlations between esds in cell parameters are only used when they are defined by crystal symmetry. An approximate (isotropic) treatment of cell esds is used for estimating esds involving l.s. planes.

### 3-(Furan-2-yl)-4-(4-methoxyphenyl)-1-(3,4,5-trimethoxyphenyl)azetidin-2-one (2) Fractional atomic coordinates and isotropic or equivalent isotropic displacement parameters (Å^2^)

**Table:**

	*x*	*y*	*z*	*U*_iso_*/*U*_eq_	
C2	0.47248 (11)	0.3915 (2)	0.20698 (9)	0.0191 (4)	
C3	0.54420 (10)	0.3731 (2)	0.19170 (9)	0.0197 (4)	
H3	0.567145	0.469890	0.189674	0.024*	
C4	0.56531 (10)	0.3031 (2)	0.25452 (9)	0.0192 (4)	
H4	0.573621	0.195483	0.252203	0.023*	
C5	0.46055 (10)	0.3049 (2)	0.30884 (9)	0.0190 (4)	
C6	0.49630 (10)	0.2258 (2)	0.35596 (9)	0.0186 (4)	
H6	0.542512	0.196365	0.356850	0.022*	
C7	0.46335 (11)	0.1905 (2)	0.40192 (9)	0.0200 (5)	
C8	0.39586 (11)	0.2346 (2)	0.40053 (9)	0.0219 (5)	
C9	0.36241 (10)	0.3206 (2)	0.35366 (9)	0.0226 (5)	
C10	0.39389 (10)	0.3545 (2)	0.30683 (9)	0.0208 (5)	
H10	0.370564	0.410008	0.274372	0.025*	
C12	0.56294 (11)	0.0660 (2)	0.45217 (10)	0.0265 (5)	
H12A	0.592766	0.151371	0.451573	0.040*	
H12B	0.564784	0.003971	0.417994	0.040*	
H12C	0.578719	0.010074	0.488339	0.040*	
C14	0.36424 (13)	0.2922 (3)	0.49058 (10)	0.0351 (6)	
H14A	0.412296	0.307489	0.509826	0.053*	
H14B	0.338187	0.254135	0.519289	0.053*	
H14C	0.344203	0.385328	0.474793	0.053*	
C16	0.26548 (12)	0.4684 (3)	0.31493 (11)	0.0346 (6)	
H16A	0.294965	0.554720	0.315525	0.052*	
H16B	0.221075	0.497658	0.324055	0.052*	
H16C	0.258105	0.423415	0.275804	0.052*	
C18	0.62031 (10)	0.3758 (2)	0.29826 (9)	0.0190 (4)	
C19	0.61448 (10)	0.5225 (2)	0.31302 (9)	0.0220 (5)	
H19	0.575634	0.577145	0.294165	0.026*	
C20	0.66395 (11)	0.5896 (2)	0.35439 (10)	0.0240 (5)	
H20	0.658855	0.689345	0.364289	0.029*	
C21	0.72133 (11)	0.5111 (2)	0.38163 (9)	0.0230 (5)	
C22	0.72891 (11)	0.3664 (2)	0.36712 (10)	0.0267 (5)	
H22	0.768502	0.312916	0.385176	0.032*	
C23	0.67788 (11)	0.2997 (2)	0.32570 (10)	0.0248 (5)	
H23	0.682772	0.199631	0.316130	0.030*	
C25	0.83308 (12)	0.5203 (3)	0.44327 (11)	0.0376 (6)	
H25A	0.862093	0.586967	0.470516	0.056*	
H25B	0.854927	0.500759	0.409499	0.056*	
H25C	0.827427	0.428161	0.463481	0.056*	
C26	0.54927 (10)	0.2819 (2)	0.14043 (9)	0.0204 (5)	
C28	0.52460 (12)	0.0841 (2)	0.08710 (10)	0.0274 (5)	
H28	0.506039	−0.008412	0.073696	0.033*	
C29	0.56570 (11)	0.1641 (2)	0.06038 (10)	0.0276 (5)	
H29	0.580973	0.140432	0.025057	0.033*	
C30	0.58228 (11)	0.2926 (2)	0.09538 (9)	0.0257 (5)	
H30	0.611211	0.370625	0.088044	0.031*	
N1	0.49383 (8)	0.33475 (18)	0.26178 (7)	0.0186 (4)	
O11	0.49377 (7)	0.11359 (16)	0.45011 (6)	0.0243 (3)	
O13	0.36136 (7)	0.18958 (17)	0.44377 (6)	0.0274 (4)	
O15	0.29799 (7)	0.36539 (18)	0.35776 (7)	0.0299 (4)	
O17	0.41698 (7)	0.43656 (16)	0.18205 (6)	0.0233 (3)	
O24	0.76701 (7)	0.58592 (17)	0.42327 (7)	0.0283 (4)	
O27	0.51283 (7)	0.15338 (16)	0.13638 (6)	0.0262 (4)	
C2A	0.01341 (11)	0.9003 (2)	0.19312 (10)	0.0235 (5)	
C3A	−0.06450 (10)	0.8773 (2)	0.17615 (9)	0.0238 (5)	
H3A	−0.089512	0.972865	0.172892	0.029*	
C4A	−0.05884 (10)	0.8100 (2)	0.23922 (9)	0.0221 (5)	
H4A	−0.064913	0.701345	0.237090	0.027*	
C5A	0.06718 (10)	0.8324 (2)	0.29907 (9)	0.0222 (5)	
C6A	0.05023 (11)	0.7687 (2)	0.34848 (10)	0.0246 (5)	
H6A	0.004496	0.736727	0.348188	0.030*	
C7A	0.10090 (11)	0.7520 (2)	0.39857 (10)	0.0263 (5)	
C8A	0.16773 (11)	0.7963 (2)	0.39858 (10)	0.0276 (5)	
C9A	0.18367 (11)	0.8617 (2)	0.34839 (10)	0.0272 (5)	
C10A	0.13357 (10)	0.8799 (2)	0.29815 (10)	0.0243 (5)	
H10A	0.144426	0.923947	0.263904	0.029*	
C12A	0.01940 (13)	0.6680 (3)	0.45433 (11)	0.0419 (6)	
H12D	0.017145	0.638564	0.494599	0.063*	
H12E	0.000957	0.589440	0.427025	0.063*	
H12F	−0.007888	0.757043	0.444148	0.063*	
C14A	0.25700 (12)	0.6580 (3)	0.45336 (11)	0.0379 (6)	
H14D	0.285078	0.660480	0.422828	0.057*	
H14E	0.226759	0.572192	0.447434	0.057*	
H14F	0.287129	0.652246	0.492163	0.057*	
C16A	0.26520 (12)	0.9975 (3)	0.30756 (12)	0.0381 (6)	
H16D	0.257857	0.942833	0.270470	0.057*	
H16E	0.313269	1.029574	0.317547	0.057*	
H16F	0.234848	1.083105	0.303271	0.057*	
C18A	−0.09903 (10)	0.8755 (2)	0.28105 (9)	0.0214 (5)	
C19A	−0.09323 (11)	1.0232 (2)	0.29593 (11)	0.0289 (5)	
H19A	−0.064140	1.084194	0.278196	0.035*	
C20A	−0.12873 (11)	1.0823 (2)	0.33575 (11)	0.0312 (6)	
H20A	−0.124086	1.183340	0.345306	0.037*	
C21A	−0.17135 (11)	0.9945 (2)	0.36199 (10)	0.0246 (5)	
C22A	−0.17905 (11)	0.8480 (2)	0.34707 (10)	0.0243 (5)	
H22A	−0.209307	0.787966	0.363932	0.029*	
C23A	−0.14226 (10)	0.7898 (2)	0.30737 (9)	0.0233 (5)	
H23A	−0.146783	0.688701	0.297950	0.028*	
C25A	−0.25363 (12)	0.9820 (3)	0.42498 (11)	0.0310 (5)	
H25D	−0.233899	0.890512	0.442831	0.046*	
H25E	−0.292126	0.959781	0.392753	0.046*	
H25F	−0.270186	1.041064	0.454722	0.046*	
C26A	−0.09111 (11)	0.7821 (2)	0.12535 (10)	0.0250 (5)	
C28A	−0.17691 (13)	0.6706 (3)	0.06858 (11)	0.0385 (6)	
H28A	−0.222141	0.639372	0.051626	0.046*	
C29A	−0.11983 (13)	0.6254 (3)	0.05174 (11)	0.0361 (6)	
H29A	−0.116986	0.558946	0.020772	0.043*	
C30A	−0.06364 (13)	0.6962 (3)	0.08924 (11)	0.0356 (6)	
H30A	−0.016085	0.684579	0.088640	0.043*	
N1A	0.01505 (8)	0.84972 (19)	0.24877 (8)	0.0220 (4)	
O11A	0.08906 (8)	0.69564 (19)	0.45029 (7)	0.0363 (4)	
O13A	0.21642 (8)	0.78714 (19)	0.44995 (7)	0.0371 (4)	
O15A	0.25015 (8)	0.90595 (19)	0.35308 (7)	0.0346 (4)	
O17A	0.05790 (7)	0.94552 (17)	0.16854 (7)	0.0293 (4)	
O24A	−0.20227 (8)	1.06133 (17)	0.40262 (7)	0.0320 (4)	
O27A	−0.16117 (8)	0.7684 (2)	0.11372 (7)	0.0379 (4)	

### 3-(Furan-2-yl)-4-(4-methoxyphenyl)-1-(3,4,5-trimethoxyphenyl)azetidin-2-one (2) Atomic displacement parameters (Å^2^)

**Table:**

	*U*^11^	*U*^22^	*U*^33^	*U*^12^	*U*^13^	*U*^23^
C2	0.0248 (11)	0.0146 (10)	0.0181 (11)	−0.0017 (9)	0.0052 (9)	−0.0009 (9)
C3	0.0215 (11)	0.0187 (10)	0.0193 (11)	0.0014 (8)	0.0051 (9)	0.0039 (9)
C4	0.0223 (11)	0.0178 (10)	0.0193 (11)	0.0023 (8)	0.0084 (9)	0.0037 (9)
C5	0.0231 (11)	0.0171 (10)	0.0174 (11)	−0.0063 (8)	0.0054 (9)	−0.0045 (9)
C6	0.0220 (11)	0.0166 (10)	0.0179 (11)	−0.0029 (8)	0.0052 (9)	−0.0023 (9)
C7	0.0276 (12)	0.0160 (10)	0.0165 (11)	−0.0047 (8)	0.0046 (9)	−0.0017 (9)
C8	0.0258 (12)	0.0240 (11)	0.0174 (11)	−0.0073 (9)	0.0083 (9)	−0.0016 (9)
C9	0.0168 (11)	0.0269 (12)	0.0243 (12)	−0.0059 (9)	0.0047 (9)	−0.0048 (10)
C10	0.0207 (11)	0.0226 (11)	0.0183 (11)	−0.0033 (9)	0.0020 (9)	−0.0005 (9)
C12	0.0312 (12)	0.0304 (12)	0.0183 (12)	0.0048 (10)	0.0060 (9)	0.0039 (10)
C14	0.0405 (14)	0.0417 (15)	0.0264 (13)	−0.0054 (11)	0.0150 (11)	−0.0070 (11)
C16	0.0228 (12)	0.0516 (16)	0.0291 (14)	0.0048 (11)	0.0041 (10)	0.0026 (12)
C18	0.0203 (11)	0.0199 (11)	0.0181 (11)	0.0007 (8)	0.0068 (8)	0.0037 (9)
C19	0.0196 (11)	0.0213 (11)	0.0246 (12)	0.0047 (9)	0.0033 (9)	0.0036 (9)
C20	0.0258 (12)	0.0204 (11)	0.0257 (12)	0.0020 (9)	0.0043 (9)	0.0006 (9)
C21	0.0216 (11)	0.0261 (12)	0.0214 (12)	−0.0035 (9)	0.0042 (9)	0.0044 (9)
C22	0.0227 (11)	0.0255 (12)	0.0306 (13)	0.0058 (9)	0.0018 (10)	0.0084 (10)
C23	0.0283 (12)	0.0194 (11)	0.0271 (12)	0.0047 (9)	0.0067 (10)	0.0044 (9)
C25	0.0261 (13)	0.0438 (15)	0.0379 (15)	0.0004 (11)	−0.0068 (11)	0.0047 (12)
C26	0.0203 (11)	0.0187 (11)	0.0221 (11)	0.0013 (8)	0.0041 (9)	0.0031 (9)
C28	0.0359 (13)	0.0240 (12)	0.0214 (12)	0.0018 (10)	0.0031 (10)	−0.0033 (10)
C29	0.0329 (13)	0.0306 (12)	0.0201 (12)	0.0045 (10)	0.0068 (10)	−0.0005 (10)
C30	0.0271 (12)	0.0287 (12)	0.0229 (12)	−0.0019 (9)	0.0088 (10)	0.0024 (10)
N1	0.0189 (9)	0.0195 (9)	0.0182 (9)	−0.0007 (7)	0.0054 (7)	0.0020 (7)
O11	0.0283 (8)	0.0265 (8)	0.0189 (8)	0.0012 (6)	0.0066 (6)	0.0039 (7)
O13	0.0299 (8)	0.0343 (9)	0.0208 (8)	−0.0075 (7)	0.0116 (7)	0.0000 (7)
O15	0.0195 (8)	0.0453 (10)	0.0254 (9)	−0.0007 (7)	0.0059 (6)	0.0033 (7)
O17	0.0218 (8)	0.0253 (8)	0.0227 (8)	0.0014 (6)	0.0038 (6)	0.0045 (6)
O24	0.0240 (8)	0.0291 (9)	0.0283 (9)	−0.0020 (7)	−0.0037 (7)	0.0040 (7)
O27	0.0327 (9)	0.0227 (8)	0.0252 (9)	−0.0032 (7)	0.0099 (7)	−0.0005 (7)
C2A	0.0229 (11)	0.0211 (11)	0.0263 (13)	0.0016 (9)	0.0048 (10)	−0.0006 (10)
C3A	0.0195 (11)	0.0259 (11)	0.0261 (12)	0.0007 (9)	0.0046 (9)	0.0036 (10)
C4A	0.0181 (11)	0.0226 (11)	0.0245 (12)	−0.0004 (9)	0.0010 (9)	0.0027 (9)
C5A	0.0202 (11)	0.0206 (11)	0.0243 (12)	0.0030 (8)	0.0006 (9)	−0.0029 (9)
C6A	0.0206 (11)	0.0237 (11)	0.0286 (13)	0.0003 (9)	0.0022 (9)	0.0000 (10)
C7A	0.0287 (12)	0.0236 (11)	0.0257 (13)	0.0030 (9)	0.0027 (10)	−0.0003 (10)
C8A	0.0261 (12)	0.0273 (12)	0.0253 (13)	0.0055 (9)	−0.0055 (10)	−0.0045 (10)
C9A	0.0185 (11)	0.0256 (12)	0.0363 (14)	0.0016 (9)	0.0025 (10)	−0.0075 (10)
C10A	0.0203 (11)	0.0251 (11)	0.0279 (12)	0.0022 (9)	0.0057 (9)	−0.0030 (10)
C12A	0.0418 (15)	0.0535 (17)	0.0307 (14)	−0.0064 (13)	0.0075 (12)	0.0085 (13)
C14A	0.0298 (13)	0.0469 (16)	0.0350 (15)	0.0078 (11)	0.0011 (11)	0.0061 (12)
C16A	0.0217 (12)	0.0458 (16)	0.0472 (16)	−0.0046 (11)	0.0072 (11)	−0.0060 (13)
C18A	0.0172 (11)	0.0231 (11)	0.0226 (12)	0.0011 (8)	0.0004 (9)	0.0027 (9)
C19A	0.0262 (12)	0.0230 (12)	0.0398 (14)	−0.0037 (9)	0.0124 (11)	0.0034 (11)
C20A	0.0303 (13)	0.0199 (12)	0.0454 (15)	−0.0003 (9)	0.0122 (11)	−0.0009 (11)
C21A	0.0228 (11)	0.0247 (12)	0.0266 (12)	0.0049 (9)	0.0054 (10)	0.0023 (10)
C22A	0.0235 (11)	0.0246 (11)	0.0250 (12)	−0.0011 (9)	0.0052 (9)	0.0047 (10)
C23A	0.0244 (11)	0.0200 (11)	0.0239 (12)	−0.0022 (9)	0.0008 (9)	0.0008 (9)
C25A	0.0309 (13)	0.0324 (13)	0.0319 (13)	0.0033 (10)	0.0120 (11)	0.0038 (11)
C26A	0.0188 (11)	0.0317 (12)	0.0230 (12)	−0.0008 (9)	0.0005 (9)	0.0063 (10)
C28A	0.0380 (15)	0.0428 (15)	0.0313 (14)	−0.0098 (12)	−0.0016 (11)	−0.0039 (12)
C29A	0.0427 (15)	0.0367 (14)	0.0281 (14)	−0.0022 (12)	0.0048 (11)	−0.0033 (11)
C30A	0.0306 (13)	0.0400 (14)	0.0364 (14)	0.0034 (11)	0.0072 (11)	0.0010 (12)
N1A	0.0162 (9)	0.0255 (10)	0.0237 (10)	0.0004 (7)	0.0021 (7)	0.0016 (8)
O11A	0.0347 (9)	0.0438 (10)	0.0282 (9)	−0.0004 (8)	0.0003 (7)	0.0078 (8)
O13A	0.0313 (9)	0.0430 (10)	0.0317 (9)	0.0080 (8)	−0.0073 (7)	−0.0044 (8)
O15A	0.0191 (8)	0.0415 (10)	0.0409 (10)	−0.0019 (7)	0.0003 (7)	−0.0027 (8)
O17A	0.0227 (8)	0.0339 (9)	0.0322 (9)	−0.0029 (7)	0.0079 (7)	0.0040 (7)
O24A	0.0345 (9)	0.0252 (9)	0.0404 (10)	0.0022 (7)	0.0176 (8)	−0.0011 (7)
O27A	0.0264 (9)	0.0499 (11)	0.0360 (10)	−0.0033 (8)	0.0026 (7)	−0.0075 (8)

### 3-(Furan-2-yl)-4-(4-methoxyphenyl)-1-(3,4,5-trimethoxyphenyl)azetidin-2-one (2) Geometric parameters (Å, º)

**Table:**

C2—C3	1.537 (3)	C2A—C3A	1.531 (3)
C2—N1	1.361 (3)	C2A—N1A	1.364 (3)
C2—O17	1.211 (2)	C2A—O17A	1.209 (3)
C3—H3	1.0000	C3A—H3A	1.0000
C3—C4	1.571 (3)	C3A—C4A	1.569 (3)
C3—C26	1.469 (3)	C3A—C26A	1.477 (3)
C4—H4	1.0000	C4A—H4A	1.0000
C4—C18	1.493 (3)	C4A—C18A	1.492 (3)
C4—N1	1.485 (2)	C4A—N1A	1.482 (3)
C5—C6	1.384 (3)	C5A—C6A	1.382 (3)
C5—C10	1.388 (3)	C5A—C10A	1.388 (3)
C5—N1	1.404 (3)	C5A—N1A	1.409 (3)
C6—H6	0.9500	C6A—H6A	0.9500
C6—C7	1.389 (3)	C6A—C7A	1.389 (3)
C7—C8	1.390 (3)	C7A—C8A	1.383 (3)
C7—O11	1.358 (2)	C7A—O11A	1.365 (3)
C8—C9	1.399 (3)	C8A—C9A	1.396 (3)
C8—O13	1.379 (2)	C8A—O13A	1.383 (3)
C9—C10	1.386 (3)	C9A—C10A	1.386 (3)
C9—O15	1.360 (2)	C9A—O15A	1.361 (3)
C10—H10	0.9500	C10A—H10A	0.9500
C12—H12A	0.9800	C12A—H12D	0.9800
C12—H12B	0.9800	C12A—H12E	0.9800
C12—H12C	0.9800	C12A—H12F	0.9800
C12—O11	1.429 (3)	C12A—O11A	1.422 (3)
C14—H14A	0.9800	C14A—H14D	0.9800
C14—H14B	0.9800	C14A—H14E	0.9800
C14—H14C	0.9800	C14A—H14F	0.9800
C14—O13	1.426 (3)	C14A—O13A	1.421 (3)
C16—H16A	0.9800	C16A—H16D	0.9800
C16—H16B	0.9800	C16A—H16E	0.9800
C16—H16C	0.9800	C16A—H16F	0.9800
C16—O15	1.428 (3)	C16A—O15A	1.422 (3)
C18—C19	1.394 (3)	C18A—C19A	1.392 (3)
C18—C23	1.382 (3)	C18A—C23A	1.385 (3)
C19—H19	0.9500	C19A—H19A	0.9500
C19—C20	1.376 (3)	C19A—C20A	1.373 (3)
C20—H20	0.9500	C20A—H20A	0.9500
C20—C21	1.388 (3)	C20A—C21A	1.386 (3)
C21—C22	1.380 (3)	C21A—C22A	1.383 (3)
C21—O24	1.371 (3)	C21A—O24A	1.362 (3)
C22—H22	0.9500	C22A—H22A	0.9500
C22—C23	1.393 (3)	C22A—C23A	1.384 (3)
C23—H23	0.9500	C23A—H23A	0.9500
C25—H25A	0.9800	C25A—H25D	0.9800
C25—H25B	0.9800	C25A—H25E	0.9800
C25—H25C	0.9800	C25A—H25F	0.9800
C25—O24	1.433 (3)	C25A—O24A	1.425 (3)
C26—C30	1.337 (3)	C26A—C30A	1.336 (3)
C26—O27	1.373 (2)	C26A—O27A	1.367 (3)
C28—H28	0.9500	C28A—H28A	0.9500
C28—C29	1.331 (3)	C28A—C29A	1.330 (4)
C28—O27	1.364 (3)	C28A—O27A	1.365 (3)
C29—H29	0.9500	C29A—H29A	0.9500
C29—C30	1.429 (3)	C29A—C30A	1.428 (3)
C30—H30	0.9500	C30A—H30A	0.9500
			
N1—C2—C3	92.16 (16)	N1A—C2A—C3A	92.05 (16)
O17—C2—C3	136.14 (19)	O17A—C2A—C3A	135.6 (2)
O17—C2—N1	131.70 (19)	O17A—C2A—N1A	132.3 (2)
C2—C3—H3	111.2	C2A—C3A—H3A	111.0
C2—C3—C4	85.67 (15)	C2A—C3A—C4A	85.79 (15)
C4—C3—H3	111.2	C4A—C3A—H3A	111.0
C26—C3—C2	117.24 (17)	C26A—C3A—C2A	118.24 (18)
C26—C3—H3	111.2	C26A—C3A—H3A	111.0
C26—C3—C4	118.06 (17)	C26A—C3A—C4A	117.50 (18)
C3—C4—H4	111.6	C3A—C4A—H4A	110.9
C18—C4—C3	118.46 (17)	C18A—C4A—C3A	119.68 (18)
C18—C4—H4	111.6	C18A—C4A—H4A	110.9
N1—C4—C3	86.31 (14)	N1A—C4A—C3A	86.26 (15)
N1—C4—H4	111.6	N1A—C4A—H4A	110.9
N1—C4—C18	115.00 (17)	N1A—C4A—C18A	116.01 (17)
C6—C5—C10	122.39 (19)	C6A—C5A—C10A	121.7 (2)
C6—C5—N1	117.93 (18)	C6A—C5A—N1A	118.44 (18)
C10—C5—N1	119.68 (19)	C10A—C5A—N1A	119.8 (2)
C5—C6—H6	120.6	C5A—C6A—H6A	120.4
C5—C6—C7	118.77 (19)	C5A—C6A—C7A	119.2 (2)
C7—C6—H6	120.6	C7A—C6A—H6A	120.4
C6—C7—C8	120.36 (19)	C8A—C7A—C6A	120.3 (2)
O11—C7—C6	123.42 (19)	O11A—C7A—C6A	123.8 (2)
O11—C7—C8	116.21 (18)	O11A—C7A—C8A	115.8 (2)
C7—C8—C9	119.36 (18)	C7A—C8A—C9A	119.6 (2)
O13—C8—C7	120.31 (19)	O13A—C8A—C7A	119.4 (2)
O13—C8—C9	120.29 (18)	O13A—C8A—C9A	120.7 (2)
C10—C9—C8	121.06 (19)	C10A—C9A—C8A	120.7 (2)
O15—C9—C8	115.18 (18)	O15A—C9A—C8A	115.5 (2)
O15—C9—C10	123.8 (2)	O15A—C9A—C10A	123.8 (2)
C5—C10—H10	121.0	C5A—C10A—H10A	120.8
C9—C10—C5	117.9 (2)	C9A—C10A—C5A	118.4 (2)
C9—C10—H10	121.0	C9A—C10A—H10A	120.8
H12A—C12—H12B	109.5	H12D—C12A—H12E	109.5
H12A—C12—H12C	109.5	H12D—C12A—H12F	109.5
H12B—C12—H12C	109.5	H12E—C12A—H12F	109.5
O11—C12—H12A	109.5	O11A—C12A—H12D	109.5
O11—C12—H12B	109.5	O11A—C12A—H12E	109.5
O11—C12—H12C	109.5	O11A—C12A—H12F	109.5
H14A—C14—H14B	109.5	H14D—C14A—H14E	109.5
H14A—C14—H14C	109.5	H14D—C14A—H14F	109.5
H14B—C14—H14C	109.5	H14E—C14A—H14F	109.5
O13—C14—H14A	109.5	O13A—C14A—H14D	109.5
O13—C14—H14B	109.5	O13A—C14A—H14E	109.5
O13—C14—H14C	109.5	O13A—C14A—H14F	109.5
H16A—C16—H16B	109.5	H16D—C16A—H16E	109.5
H16A—C16—H16C	109.5	H16D—C16A—H16F	109.5
H16B—C16—H16C	109.5	H16E—C16A—H16F	109.5
O15—C16—H16A	109.5	O15A—C16A—H16D	109.5
O15—C16—H16B	109.5	O15A—C16A—H16E	109.5
O15—C16—H16C	109.5	O15A—C16A—H16F	109.5
C19—C18—C4	120.71 (18)	C19A—C18A—C4A	121.42 (19)
C23—C18—C4	121.15 (19)	C23A—C18A—C4A	120.67 (19)
C23—C18—C19	118.1 (2)	C23A—C18A—C19A	117.9 (2)
C18—C19—H19	119.4	C18A—C19A—H19A	119.3
C20—C19—C18	121.22 (19)	C20A—C19A—C18A	121.3 (2)
C20—C19—H19	119.4	C20A—C19A—H19A	119.3
C19—C20—H20	120.1	C19A—C20A—H20A	120.0
C19—C20—C21	119.8 (2)	C19A—C20A—C21A	120.0 (2)
C21—C20—H20	120.1	C21A—C20A—H20A	120.0
C22—C21—C20	120.1 (2)	C22A—C21A—C20A	119.8 (2)
O24—C21—C20	115.69 (19)	O24A—C21A—C20A	115.8 (2)
O24—C21—C22	124.15 (19)	O24A—C21A—C22A	124.3 (2)
C21—C22—H22	120.4	C21A—C22A—H22A	120.3
C21—C22—C23	119.3 (2)	C21A—C22A—C23A	119.4 (2)
C23—C22—H22	120.4	C23A—C22A—H22A	120.3
C18—C23—C22	121.4 (2)	C18A—C23A—H23A	119.2
C18—C23—H23	119.3	C22A—C23A—C18A	121.6 (2)
C22—C23—H23	119.3	C22A—C23A—H23A	119.2
H25A—C25—H25B	109.5	H25D—C25A—H25E	109.5
H25A—C25—H25C	109.5	H25D—C25A—H25F	109.5
H25B—C25—H25C	109.5	H25E—C25A—H25F	109.5
O24—C25—H25A	109.5	O24A—C25A—H25D	109.5
O24—C25—H25B	109.5	O24A—C25A—H25E	109.5
O24—C25—H25C	109.5	O24A—C25A—H25F	109.5
C30—C26—C3	135.1 (2)	C30A—C26A—C3A	136.0 (2)
C30—C26—O27	109.77 (18)	C30A—C26A—O27A	110.0 (2)
O27—C26—C3	115.08 (17)	O27A—C26A—C3A	113.85 (18)
C29—C28—H28	124.7	C29A—C28A—H28A	124.9
C29—C28—O27	110.6 (2)	C29A—C28A—O27A	110.3 (2)
O27—C28—H28	124.7	O27A—C28A—H28A	124.9
C28—C29—H29	126.8	C28A—C29A—H29A	126.7
C28—C29—C30	106.4 (2)	C28A—C29A—C30A	106.7 (2)
C30—C29—H29	126.8	C30A—C29A—H29A	126.7
C26—C30—C29	106.8 (2)	C26A—C30A—C29A	106.5 (2)
C26—C30—H30	126.6	C26A—C30A—H30A	126.8
C29—C30—H30	126.6	C29A—C30A—H30A	126.8
C2—N1—C4	95.79 (15)	C2A—N1A—C4A	95.65 (16)
C2—N1—C5	133.62 (17)	C2A—N1A—C5A	134.43 (18)
C5—N1—C4	130.46 (17)	C5A—N1A—C4A	129.87 (17)
C7—O11—C12	117.12 (16)	C7A—O11A—C12A	117.18 (18)
C8—O13—C14	113.77 (17)	C8A—O13A—C14A	113.03 (18)
C9—O15—C16	116.97 (17)	C9A—O15A—C16A	116.93 (18)
C21—O24—C25	117.15 (18)	C21A—O24A—C25A	118.01 (17)
C28—O27—C26	106.42 (16)	C28A—O27A—C26A	106.54 (18)
			
C2—C3—C4—C18	−118.34 (18)	C2A—C3A—C4A—C18A	121.34 (19)
C2—C3—C4—N1	−1.80 (14)	C2A—C3A—C4A—N1A	3.40 (15)
C2—C3—C26—C30	137.0 (2)	C2A—C3A—C26A—C30A	5.5 (4)
C2—C3—C26—O27	−43.8 (2)	C2A—C3A—C26A—O27A	180.00 (18)
C3—C2—N1—C4	−2.08 (16)	C3A—C2A—N1A—C4A	3.91 (17)
C3—C2—N1—C5	−178.1 (2)	C3A—C2A—N1A—C5A	−178.4 (2)
C3—C4—C18—C19	55.5 (3)	C3A—C4A—C18A—C19A	−55.5 (3)
C3—C4—C18—C23	−125.2 (2)	C3A—C4A—C18A—C23A	126.0 (2)
C3—C4—N1—C2	2.04 (16)	C3A—C4A—N1A—C2A	−3.82 (17)
C3—C4—N1—C5	178.3 (2)	C3A—C4A—N1A—C5A	178.3 (2)
C3—C26—C30—C29	179.5 (2)	C3A—C26A—C30A—C29A	175.6 (2)
C3—C26—O27—C28	−179.18 (17)	C3A—C26A—O27A—C28A	−176.12 (19)
C4—C3—C26—C30	−122.7 (3)	C4A—C3A—C26A—C30A	−95.2 (3)
C4—C3—C26—O27	56.4 (2)	C4A—C3A—C26A—O27A	79.3 (2)
C4—C18—C19—C20	178.27 (19)	C4A—C18A—C19A—C20A	−178.1 (2)
C4—C18—C23—C22	−179.13 (19)	C4A—C18A—C23A—C22A	178.84 (19)
C5—C6—C7—C8	−0.4 (3)	C5A—C6A—C7A—C8A	−1.1 (3)
C5—C6—C7—O11	−179.94 (18)	C5A—C6A—C7A—O11A	177.4 (2)
C6—C5—C10—C9	−1.1 (3)	C6A—C5A—C10A—C9A	0.3 (3)
C6—C5—N1—C2	170.6 (2)	C6A—C5A—N1A—C2A	−176.6 (2)
C6—C5—N1—C4	−4.3 (3)	C6A—C5A—N1A—C4A	0.4 (3)
C6—C7—C8—C9	−2.8 (3)	C6A—C7A—C8A—C9A	1.7 (3)
C6—C7—C8—O13	174.78 (18)	C6A—C7A—C8A—O13A	176.0 (2)
C6—C7—O11—C12	−1.0 (3)	C6A—C7A—O11A—C12A	−7.1 (3)
C7—C8—C9—C10	4.2 (3)	C7A—C8A—C9A—C10A	−1.3 (3)
C7—C8—C9—O15	−176.39 (18)	C7A—C8A—C9A—O15A	178.0 (2)
C7—C8—O13—C14	95.2 (2)	C7A—C8A—O13A—C14A	96.8 (2)
C8—C7—O11—C12	179.44 (18)	C8A—C7A—O11A—C12A	171.5 (2)
C8—C9—C10—C5	−2.2 (3)	C8A—C9A—C10A—C5A	0.4 (3)
C8—C9—O15—C16	172.20 (19)	C8A—C9A—O15A—C16A	−168.2 (2)
C9—C8—O13—C14	−87.2 (2)	C9A—C8A—O13A—C14A	−89.0 (3)
C10—C5—C6—C7	2.4 (3)	C10A—C5A—C6A—C7A	0.1 (3)
C10—C5—N1—C2	−9.4 (3)	C10A—C5A—N1A—C2A	3.8 (4)
C10—C5—N1—C4	175.72 (19)	C10A—C5A—N1A—C4A	−179.3 (2)
C10—C9—O15—C16	−8.4 (3)	C10A—C9A—O15A—C16A	11.1 (3)
C18—C4—N1—C2	121.84 (18)	C18A—C4A—N1A—C2A	−125.18 (19)
C18—C4—N1—C5	−61.9 (3)	C18A—C4A—N1A—C5A	57.0 (3)
C18—C19—C20—C21	0.8 (3)	C18A—C19A—C20A—C21A	0.0 (4)
C19—C18—C23—C22	0.1 (3)	C19A—C18A—C23A—C22A	0.3 (3)
C19—C20—C21—C22	0.3 (3)	C19A—C20A—C21A—C22A	−1.3 (3)
C19—C20—C21—O24	−178.46 (19)	C19A—C20A—C21A—O24A	177.5 (2)
C20—C21—C22—C23	−1.1 (3)	C20A—C21A—C22A—C23A	2.0 (3)
C20—C21—O24—C25	−167.21 (19)	C20A—C21A—O24A—C25A	171.6 (2)
C21—C22—C23—C18	0.9 (3)	C21A—C22A—C23A—C18A	−1.5 (3)
C22—C21—O24—C25	14.1 (3)	C22A—C21A—O24A—C25A	−9.7 (3)
C23—C18—C19—C20	−1.0 (3)	C23A—C18A—C19A—C20A	0.5 (3)
C26—C3—C4—C18	123.0 (2)	C26A—C3A—C4A—C18A	−118.9 (2)
C26—C3—C4—N1	−120.48 (18)	C26A—C3A—C4A—N1A	123.18 (19)
C28—C29—C30—C26	−0.8 (3)	C28A—C29A—C30A—C26A	−1.4 (3)
C29—C28—O27—C26	−0.7 (2)	C29A—C28A—O27A—C26A	−0.7 (3)
C30—C26—O27—C28	0.2 (2)	C30A—C26A—O27A—C28A	−0.2 (3)
N1—C2—C3—C4	1.96 (15)	N1A—C2A—C3A—C4A	−3.69 (16)
N1—C2—C3—C26	121.41 (19)	N1A—C2A—C3A—C26A	−122.8 (2)
N1—C4—C18—C19	−44.4 (3)	N1A—C4A—C18A—C19A	45.7 (3)
N1—C4—C18—C23	134.9 (2)	N1A—C4A—C18A—C23A	−132.8 (2)
N1—C5—C6—C7	−177.62 (18)	N1A—C5A—C6A—C7A	−179.48 (19)
N1—C5—C10—C9	178.89 (18)	N1A—C5A—C10A—C9A	179.85 (19)
O11—C7—C8—C9	176.79 (18)	O11A—C7A—C8A—C9A	−176.9 (2)
O11—C7—C8—O13	−5.6 (3)	O11A—C7A—C8A—O13A	−2.7 (3)
O13—C8—C9—C10	−173.46 (19)	O13A—C8A—C9A—C10A	−175.5 (2)
O13—C8—C9—O15	6.0 (3)	O13A—C8A—C9A—O15A	3.8 (3)
O15—C9—C10—C5	178.39 (19)	O15A—C9A—C10A—C5A	−178.9 (2)
O17—C2—C3—C4	−177.5 (3)	O17A—C2A—C3A—C4A	176.5 (3)
O17—C2—C3—C26	−58.0 (3)	O17A—C2A—C3A—C26A	57.4 (3)
O17—C2—N1—C4	177.4 (2)	O17A—C2A—N1A—C4A	−176.3 (2)
O17—C2—N1—C5	1.3 (4)	O17A—C2A—N1A—C5A	1.4 (4)
O24—C21—C22—C23	177.50 (19)	O24A—C21A—C22A—C23A	−176.6 (2)
O27—C26—C30—C29	0.4 (2)	O27A—C26A—C30A—C29A	1.0 (3)
O27—C28—C29—C30	0.9 (3)	O27A—C28A—C29A—C30A	1.3 (3)

### 3-(Furan-2-yl)-4-(4-methoxyphenyl)-1-(3,4,5-trimethoxyphenyl)azetidin-2-one (2) Hydrogen-bond geometry (Å, º)

**Table:**

*D*—H···*A*	*D*—H	H···*A*	*D*···*A*	*D*—H···*A*
C10—H10···O17	0.95	2.50	3.103 (3)	122
C12—H12*C*···O11^i^	0.98	2.48	3.171 (3)	127
C12—H12*C*···O13^i^	0.98	2.55	3.482 (3)	159
C22—H22···O24*A*^ii^	0.95	2.39	3.143 (3)	136
C10*A*—H10*A*···O17*A*	0.95	2.53	3.144 (3)	123
C22*A*—H22*A*···O24^iii^	0.95	2.40	3.274 (3)	153

Symmetry codes: (i) −*x*+1, −*y*, −*z*+1; (ii) *x*+1, *y*−1, *z*; (iii) *x*−1, *y*, *z*.

### 4-(4-Methoxyphenyl)-3-(naphthalen-1-yl)-1-(3,4,5-trimethoxyphenyl)azetidin-2-one (3) Crystal data

**Table:**

C_29_H_27_NO_5_	*Z* = 2
*M**_r_* = 469.51	*F*(000) = 496
Triclinic, *P*1	*D*_x_ = 1.330 Mg m^−^^3^
*a* = 10.4633 (6) Å	Cu *K*α radiation, λ = 1.54178 Å
*b* = 11.3180 (6) Å	Cell parameters from 6199 reflections
*c* = 11.6008 (6) Å	θ = 4.1–69.6°
α = 104.628 (3)°	µ = 0.74 mm^−^^1^
β = 99.056 (4)°	*T* = 100 K
γ = 112.929 (3)°	Parallelepiped, clear colourless
*V* = 1172.74 (12) Å^3^	0.27 × 0.15 × 0.04 mm

### 4-(4-Methoxyphenyl)-3-(naphthalen-1-yl)-1-(3,4,5-trimethoxyphenyl)azetidin-2-one (3) Data collection

**Table:**

Bruker APEXII Kappa Duo diffractometer	4385 independent reflections
Radiation source: microfocus sealed X-ray tube, Incoatec Iµs	3453 reflections with *I* > 2σ(*I*)
Graphite monochromator	*R*_int_ = 0.065
Detector resolution: 8.33 pixels mm^-1^	θ_max_ = 70.1°, θ_min_ = 4.1°
ω and φ scans	*h* = −12→12
Absorption correction: multi-scan (SADABS; Bruker, 2016)	*k* = −13→13
*T*_min_ = 0.598, *T*_max_ = 0.753	*l* = −13→14
18268 measured reflections	

### 4-(4-Methoxyphenyl)-3-(naphthalen-1-yl)-1-(3,4,5-trimethoxyphenyl)azetidin-2-one (3) Refinement

**Table:**

Refinement on *F*^2^	Primary atom site location: dual
Least-squares matrix: full	Hydrogen site location: inferred from neighbouring sites
*R*[*F*^2^ > 2σ(*F*^2^)] = 0.060	H-atom parameters constrained
*wR*(*F*^2^) = 0.188	*w* = 1/[σ^2^(*F*_o_^2^) + (0.1228*P*)^2^ + 0.0444*P*] where *P* = (*F*_o_^2^ + 2*F*_c_^2^)/3
*S* = 1.09	(Δ/σ)_max_ < 0.001
4385 reflections	Δρ_max_ = 0.32 e Å^−^^3^
320 parameters	Δρ_min_ = −0.37 e Å^−^^3^
0 restraints	

### 4-(4-Methoxyphenyl)-3-(naphthalen-1-yl)-1-(3,4,5-trimethoxyphenyl)azetidin-2-one (3) Special details

**Table:**

Geometry. All esds (except the esd in the dihedral angle between two l.s. planes) are estimated using the full covariance matrix. The cell esds are taken into account individually in the estimation of esds in distances, angles and torsion angles; correlations between esds in cell parameters are only used when they are defined by crystal symmetry. An approximate (isotropic) treatment of cell esds is used for estimating esds involving l.s. planes.

### 4-(4-Methoxyphenyl)-3-(naphthalen-1-yl)-1-(3,4,5-trimethoxyphenyl)azetidin-2-one (3) Fractional atomic coordinates and isotropic or equivalent isotropic displacement parameters (Å^2^)

**Table:**

	*x*	*y*	*z*	*U*_iso_*/*U*_eq_	
O11	0.27184 (19)	0.98480 (17)	0.56161 (14)	0.0318 (4)	
O13	0.28619 (18)	1.19859 (16)	0.49316 (14)	0.0318 (4)	
O15	0.33384 (18)	1.21545 (16)	0.27846 (14)	0.0310 (4)	
O17	0.50893 (19)	0.88524 (17)	0.04762 (15)	0.0335 (4)	
O24	−0.05470 (18)	0.24692 (16)	0.19767 (15)	0.0330 (4)	
N1	0.4283 (2)	0.81934 (18)	0.20997 (16)	0.0252 (4)	
C2	0.4908 (2)	0.8174 (2)	0.1145 (2)	0.0262 (5)	
C3	0.5262 (2)	0.7014 (2)	0.13022 (19)	0.0260 (5)	
H3	0.465982	0.614244	0.058406	0.031*	
C4	0.4548 (2)	0.7094 (2)	0.2403 (2)	0.0265 (5)	
H4	0.527251	0.744487	0.323123	0.032*	
C5	0.3828 (2)	0.9105 (2)	0.27668 (19)	0.0246 (4)	
C6	0.3466 (2)	0.8950 (2)	0.38390 (19)	0.0257 (4)	
H6	0.346697	0.821091	0.408594	0.031*	
C7	0.3102 (2)	0.9898 (2)	0.45462 (19)	0.0266 (5)	
C8	0.3106 (2)	1.0983 (2)	0.41895 (19)	0.0275 (5)	
C9	0.3416 (2)	1.1088 (2)	0.3075 (2)	0.0257 (4)	
C10	0.3777 (2)	1.0147 (2)	0.23476 (19)	0.0266 (5)	
H10	0.398219	1.021108	0.159038	0.032*	
C12	0.2438 (3)	0.8634 (2)	0.5902 (2)	0.0333 (5)	
H12A	0.171099	0.783988	0.519281	0.050*	
H12B	0.207551	0.868452	0.663356	0.050*	
H12C	0.333556	0.854173	0.607777	0.050*	
C14	0.1374 (3)	1.1670 (3)	0.4731 (3)	0.0387 (6)	
H14A	0.092431	1.143801	0.384612	0.058*	
H14B	0.128477	1.246329	0.521876	0.058*	
H14C	0.088702	1.089081	0.499073	0.058*	
C16	0.3114 (3)	1.2035 (2)	0.1502 (2)	0.0307 (5)	
H16A	0.227553	1.116488	0.099011	0.046*	
H16B	0.397713	1.206794	0.125353	0.046*	
H16C	0.293765	1.279194	0.138445	0.046*	
C18	0.3190 (2)	0.5863 (2)	0.22621 (19)	0.0257 (5)	
C19	0.2062 (2)	0.5191 (2)	0.11641 (19)	0.0267 (5)	
H19	0.215837	0.551660	0.048795	0.032*	
C20	0.0801 (2)	0.4060 (2)	0.1029 (2)	0.0274 (5)	
H20	0.004458	0.361916	0.026919	0.033*	
C21	0.0648 (2)	0.3571 (2)	0.2016 (2)	0.0280 (5)	
C22	0.1773 (3)	0.4226 (2)	0.3123 (2)	0.0299 (5)	
H22	0.167774	0.389856	0.379824	0.036*	
C23	0.3029 (3)	0.5352 (2)	0.3241 (2)	0.0290 (5)	
H23	0.379153	0.578520	0.399561	0.035*	
C25	−0.1770 (3)	0.1897 (2)	0.0908 (2)	0.0376 (5)	
H25A	−0.152589	0.151104	0.016782	0.056*	
H25B	−0.203443	0.261295	0.080580	0.056*	
H25C	−0.258858	0.117424	0.101861	0.056*	
C26	0.6851 (2)	0.7344 (2)	0.16019 (19)	0.0280 (5)	
C27	0.7848 (3)	0.8487 (2)	0.1473 (2)	0.0310 (5)	
H27	0.753751	0.909115	0.122201	0.037*	
C28	0.9327 (3)	0.8786 (3)	0.1706 (2)	0.0348 (5)	
H28	0.999336	0.957458	0.159343	0.042*	
C29	0.9801 (3)	0.7948 (3)	0.2092 (2)	0.0335 (5)	
H29	1.079396	0.814681	0.223379	0.040*	
C30	0.8821 (3)	0.6781 (2)	0.2281 (2)	0.0313 (5)	
C31	0.9306 (3)	0.5940 (3)	0.2752 (2)	0.0359 (6)	
H31	1.029853	0.613391	0.290242	0.043*	
C32	0.8369 (3)	0.4857 (3)	0.2994 (2)	0.0408 (6)	
H32	0.871431	0.431380	0.332242	0.049*	
C33	0.6895 (3)	0.4547 (3)	0.2755 (3)	0.0392 (6)	
H33	0.624951	0.379803	0.293207	0.047*	
C34	0.6376 (3)	0.5316 (2)	0.2268 (2)	0.0347 (5)	
H34	0.537114	0.507401	0.208794	0.042*	
C35	0.7323 (2)	0.6464 (2)	0.2033 (2)	0.0289 (5)	

### 4-(4-Methoxyphenyl)-3-(naphthalen-1-yl)-1-(3,4,5-trimethoxyphenyl)azetidin-2-one (3) Atomic displacement parameters (Å^2^)

**Table:**

	*U*^11^	*U*^22^	*U*^33^	*U*^12^	*U*^13^	*U*^23^
O11	0.0418 (9)	0.0346 (9)	0.0217 (7)	0.0216 (7)	0.0101 (7)	0.0060 (6)
O13	0.0374 (9)	0.0288 (8)	0.0259 (8)	0.0197 (7)	0.0064 (6)	−0.0019 (6)
O15	0.0426 (9)	0.0261 (8)	0.0262 (8)	0.0201 (7)	0.0106 (7)	0.0041 (6)
O17	0.0398 (9)	0.0336 (9)	0.0345 (9)	0.0219 (7)	0.0173 (7)	0.0109 (7)
O24	0.0339 (9)	0.0256 (8)	0.0346 (8)	0.0113 (7)	0.0078 (7)	0.0074 (6)
N1	0.0282 (9)	0.0220 (9)	0.0256 (9)	0.0142 (7)	0.0088 (7)	0.0032 (7)
C2	0.0267 (11)	0.0246 (10)	0.0249 (10)	0.0126 (9)	0.0066 (8)	0.0034 (8)
C3	0.0280 (11)	0.0211 (10)	0.0233 (10)	0.0114 (8)	0.0053 (8)	−0.0008 (8)
C4	0.0286 (11)	0.0241 (10)	0.0251 (10)	0.0144 (9)	0.0068 (8)	0.0020 (8)
C5	0.0204 (10)	0.0216 (10)	0.0238 (10)	0.0089 (8)	0.0017 (8)	−0.0014 (8)
C6	0.0234 (10)	0.0242 (10)	0.0237 (10)	0.0102 (8)	0.0026 (8)	0.0021 (8)
C7	0.0264 (11)	0.0290 (11)	0.0197 (9)	0.0136 (9)	0.0036 (8)	0.0011 (8)
C8	0.0278 (11)	0.0275 (11)	0.0210 (10)	0.0140 (9)	0.0033 (8)	−0.0019 (8)
C9	0.0248 (10)	0.0214 (10)	0.0250 (10)	0.0106 (8)	0.0032 (8)	0.0006 (8)
C10	0.0273 (11)	0.0250 (10)	0.0240 (10)	0.0120 (9)	0.0073 (8)	0.0022 (8)
C12	0.0383 (13)	0.0344 (12)	0.0249 (10)	0.0159 (10)	0.0095 (9)	0.0067 (9)
C14	0.0397 (14)	0.0311 (12)	0.0471 (14)	0.0199 (11)	0.0189 (11)	0.0058 (10)
C16	0.0369 (12)	0.0272 (11)	0.0284 (11)	0.0169 (10)	0.0098 (9)	0.0060 (9)
C18	0.0282 (11)	0.0208 (10)	0.0259 (10)	0.0140 (9)	0.0066 (8)	0.0006 (8)
C19	0.0313 (11)	0.0254 (10)	0.0236 (10)	0.0153 (9)	0.0081 (8)	0.0044 (8)
C20	0.0288 (11)	0.0235 (10)	0.0246 (10)	0.0124 (9)	0.0040 (8)	0.0009 (8)
C21	0.0295 (11)	0.0218 (10)	0.0317 (11)	0.0135 (9)	0.0103 (9)	0.0038 (8)
C22	0.0349 (12)	0.0263 (11)	0.0293 (11)	0.0160 (9)	0.0083 (9)	0.0077 (8)
C23	0.0326 (12)	0.0275 (11)	0.0246 (10)	0.0154 (9)	0.0044 (9)	0.0046 (8)
C25	0.0340 (13)	0.0284 (12)	0.0369 (12)	0.0060 (10)	0.0058 (10)	0.0057 (10)
C26	0.0294 (12)	0.0272 (11)	0.0210 (9)	0.0142 (9)	0.0049 (8)	−0.0028 (8)
C27	0.0318 (12)	0.0323 (11)	0.0241 (10)	0.0147 (9)	0.0073 (9)	0.0019 (9)
C28	0.0292 (12)	0.0356 (12)	0.0284 (11)	0.0103 (10)	0.0063 (9)	0.0015 (9)
C29	0.0247 (11)	0.0399 (13)	0.0253 (10)	0.0129 (10)	0.0047 (8)	−0.0014 (9)
C30	0.0303 (12)	0.0319 (11)	0.0230 (10)	0.0167 (10)	0.0037 (8)	−0.0061 (8)
C31	0.0339 (13)	0.0354 (12)	0.0333 (12)	0.0223 (11)	0.0040 (10)	−0.0033 (10)
C32	0.0439 (15)	0.0338 (13)	0.0422 (14)	0.0250 (12)	0.0047 (11)	0.0018 (10)
C33	0.0391 (14)	0.0284 (12)	0.0480 (14)	0.0186 (10)	0.0091 (11)	0.0067 (10)
C34	0.0292 (12)	0.0286 (11)	0.0390 (12)	0.0138 (10)	0.0070 (10)	0.0009 (9)
C35	0.0278 (11)	0.0259 (10)	0.0241 (10)	0.0128 (9)	0.0036 (8)	−0.0045 (8)

### 4-(4-Methoxyphenyl)-3-(naphthalen-1-yl)-1-(3,4,5-trimethoxyphenyl)azetidin-2-one (3) Geometric parameters (Å, º)

**Table:**

O11—C7	1.371 (3)	C16—H16B	0.9800
O11—C12	1.426 (3)	C16—H16C	0.9800
O13—C8	1.377 (2)	C18—C19	1.390 (3)
O13—C14	1.420 (3)	C18—C23	1.402 (3)
O15—C9	1.360 (3)	C19—H19	0.9500
O15—C16	1.432 (3)	C19—C20	1.387 (3)
O17—C2	1.209 (3)	C20—H20	0.9500
O24—C21	1.363 (3)	C20—C21	1.397 (3)
O24—C25	1.429 (3)	C21—C22	1.396 (3)
N1—C2	1.372 (3)	C22—H22	0.9500
N1—C4	1.486 (3)	C22—C23	1.386 (3)
N1—C5	1.409 (3)	C23—H23	0.9500
C2—C3	1.540 (3)	C25—H25A	0.9800
C3—H3	1.0000	C25—H25B	0.9800
C3—C4	1.578 (3)	C25—H25C	0.9800
C3—C26	1.514 (3)	C26—C27	1.370 (3)
C4—H4	1.0000	C26—C35	1.429 (3)
C4—C18	1.502 (3)	C27—H27	0.9500
C5—C6	1.388 (3)	C27—C28	1.413 (3)
C5—C10	1.400 (3)	C28—H28	0.9500
C6—H6	0.9500	C28—C29	1.363 (4)
C6—C7	1.394 (3)	C29—H29	0.9500
C7—C8	1.390 (3)	C29—C30	1.416 (4)
C8—C9	1.404 (3)	C30—C31	1.420 (3)
C9—C10	1.396 (3)	C30—C35	1.427 (3)
C10—H10	0.9500	C31—H31	0.9500
C12—H12A	0.9800	C31—C32	1.366 (4)
C12—H12B	0.9800	C32—H32	0.9500
C12—H12C	0.9800	C32—C33	1.405 (4)
C14—H14A	0.9800	C33—H33	0.9500
C14—H14B	0.9800	C33—C34	1.375 (4)
C14—H14C	0.9800	C34—H34	0.9500
C16—H16A	0.9800	C34—C35	1.416 (3)
			
C7—O11—C12	117.44 (17)	H16A—C16—H16C	109.5
C8—O13—C14	113.86 (17)	H16B—C16—H16C	109.5
C9—O15—C16	116.64 (16)	C19—C18—C4	121.4 (2)
C21—O24—C25	116.74 (18)	C19—C18—C23	118.0 (2)
C2—N1—C4	95.51 (16)	C23—C18—C4	120.6 (2)
C2—N1—C5	133.37 (19)	C18—C19—H19	119.1
C5—N1—C4	129.86 (18)	C20—C19—C18	121.8 (2)
O17—C2—N1	131.4 (2)	C20—C19—H19	119.1
O17—C2—C3	136.3 (2)	C19—C20—H20	120.2
N1—C2—C3	92.34 (17)	C19—C20—C21	119.6 (2)
C2—C3—H3	111.6	C21—C20—H20	120.2
C2—C3—C4	85.48 (16)	O24—C21—C20	124.1 (2)
C4—C3—H3	111.6	O24—C21—C22	116.5 (2)
C26—C3—C2	115.81 (18)	C22—C21—C20	119.4 (2)
C26—C3—H3	111.6	C21—C22—H22	119.9
C26—C3—C4	118.38 (17)	C23—C22—C21	120.2 (2)
N1—C4—C3	86.66 (16)	C23—C22—H22	119.9
N1—C4—H4	112.2	C18—C23—H23	119.5
N1—C4—C18	113.86 (18)	C22—C23—C18	121.0 (2)
C3—C4—H4	112.2	C22—C23—H23	119.5
C18—C4—C3	117.43 (17)	O24—C25—H25A	109.5
C18—C4—H4	112.2	O24—C25—H25B	109.5
C6—C5—N1	118.38 (19)	O24—C25—H25C	109.5
C6—C5—C10	122.10 (19)	H25A—C25—H25B	109.5
C10—C5—N1	119.51 (19)	H25A—C25—H25C	109.5
C5—C6—H6	120.6	H25B—C25—H25C	109.5
C5—C6—C7	118.7 (2)	C27—C26—C3	120.7 (2)
C7—C6—H6	120.6	C27—C26—C35	119.4 (2)
O11—C7—C6	124.1 (2)	C35—C26—C3	119.9 (2)
O11—C7—C8	115.20 (19)	C26—C27—H27	119.3
C8—C7—C6	120.7 (2)	C26—C27—C28	121.5 (2)
O13—C8—C7	121.2 (2)	C28—C27—H27	119.3
O13—C8—C9	119.2 (2)	C27—C28—H28	119.9
C7—C8—C9	119.57 (19)	C29—C28—C27	120.2 (2)
O15—C9—C8	115.04 (18)	C29—C28—H28	119.9
O15—C9—C10	124.3 (2)	C28—C29—H29	119.8
C10—C9—C8	120.7 (2)	C28—C29—C30	120.4 (2)
C5—C10—H10	121.0	C30—C29—H29	119.8
C9—C10—C5	118.1 (2)	C29—C30—C31	121.3 (2)
C9—C10—H10	121.0	C29—C30—C35	119.7 (2)
O11—C12—H12A	109.5	C31—C30—C35	119.1 (2)
O11—C12—H12B	109.5	C30—C31—H31	119.5
O11—C12—H12C	109.5	C32—C31—C30	121.0 (2)
H12A—C12—H12B	109.5	C32—C31—H31	119.5
H12A—C12—H12C	109.5	C31—C32—H32	120.0
H12B—C12—H12C	109.5	C31—C32—C33	120.0 (2)
O13—C14—H14A	109.5	C33—C32—H32	120.0
O13—C14—H14B	109.5	C32—C33—H33	119.6
O13—C14—H14C	109.5	C34—C33—C32	120.7 (3)
H14A—C14—H14B	109.5	C34—C33—H33	119.6
H14A—C14—H14C	109.5	C33—C34—H34	119.6
H14B—C14—H14C	109.5	C33—C34—C35	120.8 (2)
O15—C16—H16A	109.5	C35—C34—H34	119.6
O15—C16—H16B	109.5	C30—C35—C26	118.7 (2)
O15—C16—H16C	109.5	C34—C35—C26	122.8 (2)
H16A—C16—H16B	109.5	C34—C35—C30	118.4 (2)
			
O11—C7—C8—O13	5.3 (3)	C6—C7—C8—O13	−174.7 (2)
O11—C7—C8—C9	−176.97 (19)	C6—C7—C8—C9	2.9 (3)
O13—C8—C9—O15	−4.7 (3)	C7—C8—C9—O15	177.54 (19)
O13—C8—C9—C10	175.13 (19)	C7—C8—C9—C10	−2.6 (3)
O15—C9—C10—C5	179.47 (19)	C8—C9—C10—C5	−0.4 (3)
O17—C2—C3—C4	178.8 (3)	C10—C5—C6—C7	−2.8 (3)
O17—C2—C3—C26	59.3 (3)	C12—O11—C7—C6	−11.1 (3)
O24—C21—C22—C23	179.86 (19)	C12—O11—C7—C8	168.8 (2)
N1—C2—C3—C4	−0.65 (16)	C14—O13—C8—C7	−86.9 (3)
N1—C2—C3—C26	−120.09 (18)	C14—O13—C8—C9	95.4 (2)
N1—C4—C18—C19	53.2 (3)	C16—O15—C9—C8	−157.75 (19)
N1—C4—C18—C23	−127.1 (2)	C16—O15—C9—C10	22.4 (3)
N1—C5—C6—C7	176.06 (19)	C18—C19—C20—C21	−0.1 (3)
N1—C5—C10—C9	−175.73 (19)	C19—C18—C23—C22	−1.0 (3)
C2—N1—C4—C3	−0.68 (17)	C19—C20—C21—O24	179.90 (18)
C2—N1—C4—C18	−119.28 (19)	C19—C20—C21—C22	−0.3 (3)
C2—N1—C5—C6	−168.6 (2)	C20—C21—C22—C23	0.1 (3)
C2—N1—C5—C10	10.3 (4)	C21—C22—C23—C18	0.6 (3)
C2—C3—C4—N1	0.60 (15)	C23—C18—C19—C20	0.8 (3)
C2—C3—C4—C18	115.8 (2)	C25—O24—C21—C20	−7.7 (3)
C2—C3—C26—C27	−13.3 (3)	C25—O24—C21—C22	172.53 (19)
C2—C3—C26—C35	166.16 (18)	C26—C3—C4—N1	117.59 (19)
C3—C4—C18—C19	−45.9 (3)	C26—C3—C4—C18	−127.2 (2)
C3—C4—C18—C23	133.8 (2)	C26—C27—C28—C29	−1.3 (3)
C3—C26—C27—C28	−177.73 (19)	C27—C26—C35—C30	−1.9 (3)
C3—C26—C35—C30	178.56 (17)	C27—C26—C35—C34	175.7 (2)
C3—C26—C35—C34	−3.8 (3)	C27—C28—C29—C30	−1.0 (3)
C4—N1—C2—O17	−178.8 (2)	C28—C29—C30—C31	−176.2 (2)
C4—N1—C2—C3	0.70 (17)	C28—C29—C30—C35	1.7 (3)
C4—N1—C5—C6	−4.7 (3)	C29—C30—C31—C32	176.6 (2)
C4—N1—C5—C10	174.2 (2)	C29—C30—C35—C26	−0.3 (3)
C4—C3—C26—C27	−112.6 (2)	C29—C30—C35—C34	−178.04 (19)
C4—C3—C26—C35	66.8 (2)	C30—C31—C32—C33	1.1 (4)
C4—C18—C19—C20	−179.57 (18)	C31—C30—C35—C26	177.73 (19)
C4—C18—C23—C22	179.31 (19)	C31—C30—C35—C34	0.0 (3)
C5—N1—C2—O17	−11.1 (4)	C31—C32—C33—C34	0.6 (4)
C5—N1—C2—C3	168.4 (2)	C32—C33—C34—C35	−2.0 (4)
C5—N1—C4—C3	−169.0 (2)	C33—C34—C35—C26	−176.0 (2)
C5—N1—C4—C18	72.4 (3)	C33—C34—C35—C30	1.7 (3)
C5—C6—C7—O11	179.61 (19)	C35—C26—C27—C28	2.8 (3)
C5—C6—C7—C8	−0.3 (3)	C35—C30—C31—C32	−1.4 (3)
C6—C5—C10—C9	3.1 (3)		

### 4-(4-Methoxyphenyl)-3-(naphthalen-1-yl)-1-(3,4,5-trimethoxyphenyl)azetidin-2-one (3) Hydrogen-bond geometry (Å, º)

**Table:**

*D*—H···*A*	*D*—H	H···*A*	*D*···*A*	*D*—H···*A*
C4—H4···O13^i^	1.00	2.41	3.396 (3)	171
C10—H10···O17	0.95	2.50	3.105 (3)	122
C16—H16*B*···O17^ii^	0.98	2.53	3.387 (3)	146
C27—H27···O17	0.95	2.46	3.156 (3)	131

Symmetry codes: (i) −*x*+1, −*y*+2, −*z*+1; (ii) −*x*+1, −*y*+2, −*z*.

### 3-(3,4-Dimethoxyphenyl)-4-(4-methoxyphenyl)-1-(3,4,5-trimethoxyphenyl)azetidin-2-one (4) Crystal data

**Table:**

C_27_H_29_NO_7_	*F*(000) = 1016
*M**_r_* = 479.51	*D*_x_ = 1.309 Mg m^−^^3^
Monoclinic, *P*2_1_/*c*	Mo *K*α radiation, λ = 0.71073 Å
*a* = 8.858 (2) Å	Cell parameters from 1212 reflections
*b* = 22.769 (5) Å	θ = 2.6–23.1°
*c* = 12.822 (2) Å	µ = 0.10 mm^−^^1^
β = 109.839 (6)°	*T* = 100 K
*V* = 2432.4 (9) Å^3^	Block, clear colourless
*Z* = 4	0.41 × 0.24 × 0.12 mm

### 3-(3,4-Dimethoxyphenyl)-4-(4-methoxyphenyl)-1-(3,4,5-trimethoxyphenyl)azetidin-2-one (4) Data collection

**Table:**

Bruker D8 Quest ECO diffractometer	5620 independent reflections
Radiation source: standard sealed X-ray tube, Siemens, KFF Mo 2K -90 C	4062 reflections with *I* > 2σ(*I*)
Graphite monochromator	*R*_int_ = 0.058
Detector resolution: 5.12 pixels mm^-1^	θ_max_ = 27.6°, θ_min_ = 3.0°
ω and φ scans	*h* = −11→11
Absorption correction: multi-scan (SADABS; Bruker, 2016)	*k* = −29→29
*T*_min_ = 0.701, *T*_max_ = 0.746	*l* = −16→16
27592 measured reflections	

### 3-(3,4-Dimethoxyphenyl)-4-(4-methoxyphenyl)-1-(3,4,5-trimethoxyphenyl)azetidin-2-one (4) Refinement

**Table:**

Refinement on *F*^2^	Primary atom site location: dual
Least-squares matrix: full	Hydrogen site location: inferred from neighbouring sites
*R*[*F*^2^ > 2σ(*F*^2^)] = 0.045	H-atom parameters constrained
*wR*(*F*^2^) = 0.106	*w* = 1/[σ^2^(*F*_o_^2^) + (0.041*P*)^2^ + 1.0883*P*] where *P* = (*F*_o_^2^ + 2*F*_c_^2^)/3
*S* = 1.03	(Δ/σ)_max_ < 0.001
5620 reflections	Δρ_max_ = 0.29 e Å^−^^3^
322 parameters	Δρ_min_ = −0.24 e Å^−^^3^
0 restraints	

### 3-(3,4-Dimethoxyphenyl)-4-(4-methoxyphenyl)-1-(3,4,5-trimethoxyphenyl)azetidin-2-one (4) Special details

**Table:**

Geometry. All esds (except the esd in the dihedral angle between two l.s. planes) are estimated using the full covariance matrix. The cell esds are taken into account individually in the estimation of esds in distances, angles and torsion angles; correlations between esds in cell parameters are only used when they are defined by crystal symmetry. An approximate (isotropic) treatment of cell esds is used for estimating esds involving l.s. planes.

### 3-(3,4-Dimethoxyphenyl)-4-(4-methoxyphenyl)-1-(3,4,5-trimethoxyphenyl)azetidin-2-one (4) Fractional atomic coordinates and isotropic or equivalent isotropic displacement parameters (Å^2^)

**Table:**

	*x*	*y*	*z*	*U*_iso_*/*U*_eq_	
C2	0.84113 (18)	0.49962 (7)	0.37411 (13)	0.0162 (3)	
C3	0.87377 (18)	0.51826 (7)	0.26892 (12)	0.0157 (3)	
H3	0.990712	0.526648	0.287047	0.019*	
C4	0.78269 (18)	0.57636 (7)	0.27621 (12)	0.0148 (3)	
H4	0.674264	0.577567	0.217418	0.018*	
C5	0.71160 (18)	0.57666 (7)	0.45968 (12)	0.0159 (3)	
C6	0.65806 (18)	0.63452 (7)	0.44685 (13)	0.0172 (3)	
H6	0.662260	0.657388	0.385923	0.021*	
C7	0.59827 (18)	0.65825 (7)	0.52487 (13)	0.0180 (3)	
C8	0.59158 (19)	0.62472 (7)	0.61437 (13)	0.0191 (3)	
C9	0.63928 (19)	0.56597 (7)	0.62198 (13)	0.0184 (3)	
C10	0.70149 (18)	0.54172 (7)	0.54604 (13)	0.0172 (3)	
H10	0.736532	0.50	0.552834	0.021*	
C12	0.5546 (2)	0.75159 (7)	0.43424 (15)	0.0287 (4)	
H12A	0.666405	0.754183	0.437184	0.043*	
H12B	0.515127	0.790932	0.442413	0.043*	
H12C	0.488671	0.734895	0.362860	0.043*	
C14	0.3821 (2)	0.66089 (8)	0.67118 (15)	0.0280 (4)	
H14A	0.320459	0.625990	0.636791	0.042*	
H14B	0.351633	0.693908	0.619170	0.042*	
H14C	0.359175	0.670784	0.738701	0.042*	
C16	0.65212 (19)	0.47368 (7)	0.71345 (14)	0.0206 (3)	
H16A	0.621470	0.456024	0.773065	0.031*	
H16B	0.766690	0.466983	0.727773	0.031*	
H16C	0.589438	0.455723	0.642492	0.031*	
C18	0.86952 (18)	0.63357 (7)	0.28249 (12)	0.0156 (3)	
C19	1.02182 (19)	0.64340 (7)	0.36055 (13)	0.0180 (3)	
H19	1.072542	0.613428	0.412061	0.022*	
C20	1.09906 (19)	0.69640 (7)	0.36331 (13)	0.0196 (3)	
H20	1.202840	0.702656	0.416259	0.024*	
C21	1.02479 (19)	0.74069 (7)	0.28846 (13)	0.0186 (3)	
C22	0.8729 (2)	0.73193 (7)	0.21209 (14)	0.0212 (3)	
H22	0.821209	0.762239	0.161711	0.025*	
C23	0.79668 (19)	0.67835 (7)	0.20982 (13)	0.0186 (3)	
H23	0.692445	0.672332	0.157316	0.022*	
C25	1.0344 (2)	0.83756 (7)	0.22111 (17)	0.0295 (4)	
H25A	0.933370	0.848261	0.231571	0.044*	
H25B	1.012457	0.824058	0.144805	0.044*	
H25C	1.105287	0.871891	0.235303	0.044*	
C26	0.81566 (18)	0.47948 (7)	0.16849 (12)	0.0160 (3)	
C27	0.65677 (18)	0.45884 (7)	0.13051 (12)	0.0155 (3)	
H27	0.584360	0.470964	0.166563	0.019*	
C28	0.60498 (18)	0.42096 (7)	0.04097 (13)	0.0162 (3)	
C29	0.7138 (2)	0.40170 (7)	−0.01069 (13)	0.0186 (3)	
C30	0.8684 (2)	0.42271 (7)	0.02550 (14)	0.0211 (3)	
H30	0.941328	0.410543	−0.010129	0.025*	
C31	0.91876 (19)	0.46188 (7)	0.11445 (13)	0.0197 (3)	
H31	1.025421	0.476615	0.138081	0.024*	
C33	0.33850 (19)	0.41571 (7)	0.04667 (14)	0.0211 (3)	
H33A	0.374581	0.402140	0.123772	0.032*	
H33B	0.234442	0.397885	0.005896	0.032*	
H33C	0.327728	0.458564	0.044877	0.032*	
C35	0.7669 (2)	0.33184 (8)	−0.13163 (16)	0.0336 (4)	
H35A	0.847282	0.313216	−0.067872	0.050*	
H35B	0.819835	0.359731	−0.166161	0.050*	
H35C	0.711845	0.301696	−0.185730	0.050*	
N1	0.77324 (15)	0.55260 (5)	0.38186 (10)	0.0153 (3)	
O11	0.54523 (14)	0.71481 (5)	0.52208 (9)	0.0219 (3)	
O13	0.54909 (14)	0.64926 (5)	0.69862 (9)	0.0237 (3)	
O15	0.62117 (15)	0.53530 (5)	0.70912 (10)	0.0250 (3)	
O17	0.86311 (14)	0.45559 (5)	0.43039 (9)	0.0210 (3)	
O24	1.11080 (14)	0.79146 (5)	0.29664 (10)	0.0247 (3)	
O32	0.45374 (13)	0.39892 (5)	−0.00387 (9)	0.0193 (2)	
O34	0.65265 (14)	0.36228 (5)	−0.09539 (10)	0.0243 (3)	

### 3-(3,4-Dimethoxyphenyl)-4-(4-methoxyphenyl)-1-(3,4,5-trimethoxyphenyl)azetidin-2-one (4) Atomic displacement parameters (Å^2^)

**Table:**

	*U*^11^	*U*^22^	*U*^33^	*U*^12^	*U*^13^	*U*^23^
C2	0.0141 (7)	0.0173 (8)	0.0137 (7)	−0.0023 (6)	0.0002 (6)	−0.0024 (6)
C3	0.0149 (7)	0.0166 (7)	0.0137 (7)	0.0005 (6)	0.0022 (6)	−0.0003 (6)
C4	0.0162 (7)	0.0161 (7)	0.0117 (7)	−0.0016 (6)	0.0042 (6)	0.0010 (6)
C5	0.0134 (7)	0.0186 (7)	0.0141 (7)	−0.0030 (6)	0.0025 (6)	−0.0016 (6)
C6	0.0179 (7)	0.0174 (7)	0.0155 (7)	−0.0026 (6)	0.0045 (6)	0.0013 (6)
C7	0.0172 (7)	0.0158 (7)	0.0178 (8)	0.0002 (6)	0.0018 (6)	−0.0018 (6)
C8	0.0202 (8)	0.0231 (8)	0.0142 (7)	0.0014 (6)	0.0062 (6)	−0.0019 (6)
C9	0.0184 (8)	0.0220 (8)	0.0142 (7)	−0.0011 (6)	0.0046 (6)	0.0032 (6)
C10	0.0184 (7)	0.0155 (7)	0.0166 (8)	0.0003 (6)	0.0045 (6)	0.0024 (6)
C12	0.0444 (11)	0.0174 (8)	0.0211 (9)	0.0034 (8)	0.0071 (8)	0.0029 (7)
C14	0.0256 (9)	0.0335 (10)	0.0279 (10)	−0.0012 (8)	0.0130 (8)	−0.0089 (8)
C16	0.0206 (8)	0.0214 (8)	0.0195 (8)	−0.0009 (7)	0.0065 (7)	0.0041 (6)
C18	0.0177 (7)	0.0169 (7)	0.0131 (7)	−0.0020 (6)	0.0065 (6)	−0.0016 (6)
C19	0.0187 (8)	0.0188 (8)	0.0153 (8)	0.0006 (6)	0.0045 (6)	0.0017 (6)
C20	0.0171 (8)	0.0222 (8)	0.0168 (8)	−0.0033 (6)	0.0023 (6)	−0.0020 (6)
C21	0.0201 (8)	0.0165 (8)	0.0202 (8)	−0.0042 (6)	0.0081 (7)	−0.0032 (6)
C22	0.0224 (8)	0.0167 (8)	0.0210 (8)	0.0002 (6)	0.0027 (7)	0.0030 (6)
C23	0.0176 (8)	0.0192 (8)	0.0160 (8)	−0.0023 (6)	0.0019 (6)	−0.0010 (6)
C25	0.0274 (9)	0.0163 (8)	0.0425 (11)	−0.0036 (7)	0.0091 (8)	0.0043 (8)
C26	0.0191 (7)	0.0152 (7)	0.0123 (7)	0.0003 (6)	0.0034 (6)	0.0014 (6)
C27	0.0177 (7)	0.0148 (7)	0.0140 (7)	0.0020 (6)	0.0055 (6)	0.0009 (6)
C28	0.0186 (7)	0.0149 (7)	0.0136 (7)	−0.0018 (6)	0.0036 (6)	0.0025 (6)
C29	0.0256 (8)	0.0164 (8)	0.0131 (7)	−0.0008 (6)	0.0059 (6)	−0.0017 (6)
C30	0.0221 (8)	0.0232 (8)	0.0211 (8)	0.0020 (7)	0.0112 (7)	−0.0024 (7)
C31	0.0166 (7)	0.0218 (8)	0.0204 (8)	−0.0014 (6)	0.0061 (6)	−0.0013 (6)
C33	0.0185 (8)	0.0262 (9)	0.0184 (8)	−0.0038 (7)	0.0058 (6)	−0.0013 (7)
C35	0.0427 (11)	0.0297 (10)	0.0326 (11)	−0.0002 (9)	0.0185 (9)	−0.0140 (8)
N1	0.0182 (6)	0.0146 (6)	0.0133 (6)	0.0000 (5)	0.0054 (5)	0.0021 (5)
O11	0.0282 (6)	0.0153 (6)	0.0218 (6)	0.0027 (5)	0.0078 (5)	−0.0001 (5)
O13	0.0258 (6)	0.0283 (6)	0.0178 (6)	0.0032 (5)	0.0082 (5)	−0.0034 (5)
O15	0.0366 (7)	0.0226 (6)	0.0205 (6)	0.0035 (5)	0.0158 (5)	0.0053 (5)
O17	0.0275 (6)	0.0156 (6)	0.0166 (6)	0.0012 (5)	0.0029 (5)	0.0025 (4)
O24	0.0215 (6)	0.0164 (6)	0.0326 (7)	−0.0058 (5)	0.0047 (5)	0.0010 (5)
O32	0.0183 (6)	0.0221 (6)	0.0168 (6)	−0.0039 (5)	0.0050 (5)	−0.0034 (5)
O34	0.0292 (6)	0.0248 (6)	0.0204 (6)	−0.0031 (5)	0.0105 (5)	−0.0100 (5)

### 3-(3,4-Dimethoxyphenyl)-4-(4-methoxyphenyl)-1-(3,4,5-trimethoxyphenyl)azetidin-2-one (4) Geometric parameters (Å, º)

**Table:**

C2—C3	1.532 (2)	C18—C23	1.384 (2)
C2—N1	1.367 (2)	C19—H19	0.9500
C2—O17	1.2117 (19)	C19—C20	1.382 (2)
C3—H3	1.0000	C20—H20	0.9500
C3—C4	1.569 (2)	C20—C21	1.394 (2)
C3—C26	1.501 (2)	C21—C22	1.384 (2)
C4—H4	1.0000	C21—O24	1.3687 (19)
C4—C18	1.501 (2)	C22—H22	0.9500
C4—N1	1.4873 (19)	C22—C23	1.390 (2)
C5—C6	1.391 (2)	C23—H23	0.9500
C5—C10	1.391 (2)	C25—H25A	0.9800
C5—N1	1.4012 (19)	C25—H25B	0.9800
C6—H6	0.9500	C25—H25C	0.9800
C6—C7	1.390 (2)	C25—O24	1.433 (2)
C7—C8	1.396 (2)	C26—C27	1.405 (2)
C7—O11	1.3672 (19)	C26—C31	1.380 (2)
C8—C9	1.396 (2)	C27—H27	0.9500
C8—O13	1.3772 (19)	C27—C28	1.384 (2)
C9—C10	1.386 (2)	C28—C29	1.412 (2)
C9—O15	1.3724 (19)	C28—O32	1.3617 (18)
C10—H10	0.9500	C29—C30	1.374 (2)
C12—H12A	0.9800	C29—O34	1.3709 (19)
C12—H12B	0.9800	C30—H30	0.9500
C12—H12C	0.9800	C30—C31	1.396 (2)
C12—O11	1.428 (2)	C31—H31	0.9500
C14—H14A	0.9800	C33—H33A	0.9800
C14—H14B	0.9800	C33—H33B	0.9800
C14—H14C	0.9800	C33—H33C	0.9800
C14—O13	1.425 (2)	C33—O32	1.4342 (19)
C16—H16A	0.9800	C35—H35A	0.9800
C16—H16B	0.9800	C35—H35B	0.9800
C16—H16C	0.9800	C35—H35C	0.9800
C16—O15	1.4271 (19)	C35—O34	1.428 (2)
C18—C19	1.398 (2)		
			
N1—C2—C3	92.21 (12)	C19—C20—H20	120.0
O17—C2—C3	135.51 (15)	C19—C20—C21	120.02 (15)
O17—C2—N1	132.28 (15)	C21—C20—H20	120.0
C2—C3—H3	109.8	C22—C21—C20	120.09 (15)
C2—C3—C4	85.80 (11)	O24—C21—C20	115.97 (14)
C4—C3—H3	109.8	O24—C21—C22	123.94 (15)
C26—C3—C2	118.68 (13)	C21—C22—H22	120.3
C26—C3—H3	109.8	C21—C22—C23	119.35 (15)
C26—C3—C4	120.91 (13)	C23—C22—H22	120.3
C3—C4—H4	111.1	C18—C23—C22	121.35 (15)
C18—C4—C3	118.07 (13)	C18—C23—H23	119.3
C18—C4—H4	111.1	C22—C23—H23	119.3
N1—C4—C3	86.37 (11)	H25A—C25—H25B	109.5
N1—C4—H4	111.1	H25A—C25—H25C	109.5
N1—C4—C18	116.94 (12)	H25B—C25—H25C	109.5
C6—C5—N1	119.06 (14)	O24—C25—H25A	109.5
C10—C5—C6	121.64 (14)	O24—C25—H25B	109.5
C10—C5—N1	119.28 (14)	O24—C25—H25C	109.5
C5—C6—H6	120.6	C27—C26—C3	120.38 (14)
C7—C6—C5	118.74 (14)	C31—C26—C3	120.59 (14)
C7—C6—H6	120.6	C31—C26—C27	118.99 (14)
C6—C7—C8	120.78 (15)	C26—C27—H27	119.8
O11—C7—C6	123.97 (14)	C28—C27—C26	120.47 (14)
O11—C7—C8	115.24 (14)	C28—C27—H27	119.8
C7—C8—C9	119.01 (14)	C27—C28—C29	119.73 (14)
O13—C8—C7	121.47 (14)	O32—C28—C27	125.56 (14)
O13—C8—C9	119.38 (14)	O32—C28—C29	114.70 (13)
C10—C9—C8	121.05 (14)	C30—C29—C28	119.63 (14)
O15—C9—C8	115.35 (14)	O34—C29—C28	115.20 (14)
O15—C9—C10	123.61 (14)	O34—C29—C30	125.17 (14)
C5—C10—H10	120.7	C29—C30—H30	119.9
C9—C10—C5	118.67 (14)	C29—C30—C31	120.23 (15)
C9—C10—H10	120.7	C31—C30—H30	119.9
H12A—C12—H12B	109.5	C26—C31—C30	120.89 (15)
H12A—C12—H12C	109.5	C26—C31—H31	119.6
H12B—C12—H12C	109.5	C30—C31—H31	119.6
O11—C12—H12A	109.5	H33A—C33—H33B	109.5
O11—C12—H12B	109.5	H33A—C33—H33C	109.5
O11—C12—H12C	109.5	H33B—C33—H33C	109.5
H14A—C14—H14B	109.5	O32—C33—H33A	109.5
H14A—C14—H14C	109.5	O32—C33—H33B	109.5
H14B—C14—H14C	109.5	O32—C33—H33C	109.5
O13—C14—H14A	109.5	H35A—C35—H35B	109.5
O13—C14—H14B	109.5	H35A—C35—H35C	109.5
O13—C14—H14C	109.5	H35B—C35—H35C	109.5
H16A—C16—H16B	109.5	O34—C35—H35A	109.5
H16A—C16—H16C	109.5	O34—C35—H35B	109.5
H16B—C16—H16C	109.5	O34—C35—H35C	109.5
O15—C16—H16A	109.5	C2—N1—C4	95.31 (12)
O15—C16—H16B	109.5	C2—N1—C5	133.20 (13)
O15—C16—H16C	109.5	C5—N1—C4	131.48 (13)
C19—C18—C4	121.98 (14)	C7—O11—C12	117.48 (13)
C23—C18—C4	119.33 (14)	C8—O13—C14	114.77 (13)
C23—C18—C19	118.69 (14)	C9—O15—C16	117.25 (12)
C18—C19—H19	119.8	C21—O24—C25	116.37 (13)
C20—C19—C18	120.48 (15)	C28—O32—C33	117.39 (12)
C20—C19—H19	119.8	C29—O34—C35	116.26 (13)
			
C2—C3—C4—C18	122.60 (13)	C19—C20—C21—O24	−179.43 (14)
C2—C3—C4—N1	3.87 (10)	C20—C21—C22—C23	−1.0 (2)
C2—C3—C26—C27	49.0 (2)	C20—C21—O24—C25	−179.10 (15)
C2—C3—C26—C31	−128.69 (16)	C21—C22—C23—C18	0.1 (2)
C3—C2—N1—C4	4.44 (12)	C22—C21—O24—C25	0.7 (2)
C3—C2—N1—C5	−176.97 (15)	C23—C18—C19—C20	−1.3 (2)
C3—C4—C18—C19	−51.9 (2)	C26—C3—C4—C18	−116.28 (16)
C3—C4—C18—C23	128.81 (15)	C26—C3—C4—N1	124.99 (14)
C3—C4—N1—C2	−4.35 (12)	C26—C27—C28—C29	1.7 (2)
C3—C4—N1—C5	177.03 (15)	C26—C27—C28—O32	−178.98 (14)
C3—C26—C27—C28	−177.09 (14)	C27—C26—C31—C30	−2.0 (2)
C3—C26—C31—C30	175.73 (14)	C27—C28—C29—C30	−2.7 (2)
C4—C3—C26—C27	−54.3 (2)	C27—C28—C29—O34	177.23 (14)
C4—C3—C26—C31	128.01 (16)	C27—C28—O32—C33	−0.8 (2)
C4—C18—C19—C20	179.34 (14)	C28—C29—C30—C31	1.4 (2)
C4—C18—C23—C22	−179.58 (15)	C28—C29—O34—C35	−165.89 (15)
C5—C6—C7—C8	−0.1 (2)	C29—C28—O32—C33	178.51 (13)
C5—C6—C7—O11	178.70 (14)	C29—C30—C31—C26	1.0 (2)
C6—C5—C10—C9	−1.3 (2)	C30—C29—O34—C35	14.1 (2)
C6—C5—N1—C2	176.19 (16)	C31—C26—C27—C28	0.6 (2)
C6—C5—N1—C4	−5.7 (2)	N1—C2—C3—C4	−4.21 (11)
C6—C7—C8—C9	−2.8 (2)	N1—C2—C3—C26	−127.37 (14)
C6—C7—C8—O13	172.91 (14)	N1—C4—C18—C19	49.1 (2)
C6—C7—O11—C12	−1.0 (2)	N1—C4—C18—C23	−130.20 (15)
C7—C8—C9—C10	3.8 (2)	N1—C5—C6—C7	−179.48 (14)
C7—C8—C9—O15	−176.74 (14)	N1—C5—C10—C9	−179.57 (14)
C7—C8—O13—C14	75.6 (2)	O11—C7—C8—C9	178.24 (14)
C8—C7—O11—C12	177.94 (14)	O11—C7—C8—O13	−6.0 (2)
C8—C9—C10—C5	−1.8 (2)	O13—C8—C9—C10	−172.02 (14)
C8—C9—O15—C16	173.48 (14)	O13—C8—C9—O15	7.4 (2)
C9—C8—O13—C14	−108.73 (17)	O15—C9—C10—C5	178.82 (14)
C10—C5—C6—C7	2.3 (2)	O17—C2—C3—C4	175.09 (18)
C10—C5—N1—C2	−5.5 (2)	O17—C2—C3—C26	51.9 (2)
C10—C5—N1—C4	172.62 (14)	O17—C2—N1—C4	−174.89 (17)
C10—C9—O15—C16	−7.1 (2)	O17—C2—N1—C5	3.7 (3)
C18—C4—N1—C2	−124.13 (14)	O24—C21—C22—C23	179.19 (15)
C18—C4—N1—C5	57.3 (2)	O32—C28—C29—C30	177.87 (14)
C18—C19—C20—C21	0.4 (2)	O32—C28—C29—O34	−2.2 (2)
C19—C18—C23—C22	1.1 (2)	O34—C29—C30—C31	−178.55 (15)
C19—C20—C21—C22	0.7 (2)		

### 3-(3,4-Dimethoxyphenyl)-4-(4-methoxyphenyl)-1-(3,4,5-trimethoxyphenyl)azetidin-2-one (4) Hydrogen-bond geometry (Å, º)

**Table:**

*D*—H···*A*	*D*—H	H···*A*	*D*···*A*	*D*—H···*A*
C10—H10···O17	0.95	2.46	3.088 (2)	124
C33—H33*A*···O13^i^	0.98	2.44	3.411 (2)	170
C33—H33*A*···O15^i^	0.98	2.56	3.228 (2)	125

Symmetry code: (i) −*x*+1, −*y*+1, −*z*+1.

### 4,4-Bis(4-methoxyphenyl)-3-phenyl-1-(3,4,5-trimethoxyphenyl)azetidin-2-one (5) Crystal data

**Table:**

C_32_H_31_NO_6_	*Z* = 4
*M**_r_* = 525.58	*F*(000) = 1112
Triclinic, *P*1	*D*_x_ = 1.295 Mg m^−^^3^
*a* = 11.5720 (3) Å	Cu *K*α radiation, λ = 1.54178 Å
*b* = 12.3994 (3) Å	Cell parameters from 9822 reflections
*c* = 19.9358 (6) Å	θ = 4.0–69.1°
α = 83.779 (1)°	µ = 0.73 mm^−^^1^
β = 85.748 (1)°	*T* = 100 K
γ = 71.559 (1)°	Plate, colourless
*V* = 2695.23 (13) Å^3^	0.26 × 0.14 × 0.04 mm

### 4,4-Bis(4-methoxyphenyl)-3-phenyl-1-(3,4,5-trimethoxyphenyl)azetidin-2-one (5) Data collection

**Table:**

Bruker APEXII Kappa Duo diffractometer	9870 independent reflections
Radiation source: microfocus sealed X-ray tube, Incoatec Iµs	8278 reflections with *I* > 2σ(*I*)
Mirror optics monochromator	*R*_int_ = 0.040
Detector resolution: 8.33 pixels mm^-1^	θ_max_ = 69.2°, θ_min_ = 3.8°
ω and φ scans	*h* = −12→13
Absorption correction: multi-scan (SADABS; Bruker, 2016)	*k* = −15→14
*T*_min_ = 0.695, *T*_max_ = 0.753	*l* = −24→24
37871 measured reflections	

### 4,4-Bis(4-methoxyphenyl)-3-phenyl-1-(3,4,5-trimethoxyphenyl)azetidin-2-one (5) Refinement

**Table:**

Refinement on *F*^2^	Hydrogen site location: inferred from neighbouring sites
Least-squares matrix: full	H-atom parameters constrained
*R*[*F*^2^ > 2σ(*F*^2^)] = 0.045	*w* = 1/[σ^2^(*F*_o_^2^) + (0.082*P*)^2^ + 0.4264*P*] where *P* = (*F*_o_^2^ + 2*F*_c_^2^)/3
*wR*(*F*^2^) = 0.137	(Δ/σ)_max_ < 0.001
*S* = 1.09	Δρ_max_ = 0.24 e Å^−^^3^
9870 reflections	Δρ_min_ = −0.24 e Å^−^^3^
714 parameters	Extinction correction: SHELXL (Sheldrick, 2015b), Fc^*^=kFc[1+0.001xFc^2^λ^3^/sin(2θ)]^-1/4^
0 restraints	Extinction coefficient: 0.00057 (14)
Primary atom site location: dual	

### 4,4-Bis(4-methoxyphenyl)-3-phenyl-1-(3,4,5-trimethoxyphenyl)azetidin-2-one (5) Special details

**Table:**

Geometry. All esds (except the esd in the dihedral angle between two l.s. planes) are estimated using the full covariance matrix. The cell esds are taken into account individually in the estimation of esds in distances, angles and torsion angles; correlations between esds in cell parameters are only used when they are defined by crystal symmetry. An approximate (isotropic) treatment of cell esds is used for estimating esds involving l.s. planes.

### 4,4-Bis(4-methoxyphenyl)-3-phenyl-1-(3,4,5-trimethoxyphenyl)azetidin-2-one (5) Fractional atomic coordinates and isotropic or equivalent isotropic displacement parameters (Å^2^)

**Table:**

	*x*	*y*	*z*	*U*_iso_*/*U*_eq_	
C2	0.45948 (14)	0.12352 (13)	0.41098 (7)	0.0258 (3)	
C3	0.50570 (13)	0.00251 (12)	0.38849 (7)	0.0238 (3)	
H3	0.509131	−0.055507	0.427887	0.029*	
C4	0.37704 (13)	0.03633 (12)	0.35271 (7)	0.0227 (3)	
C5	0.24579 (13)	0.24637 (12)	0.37898 (7)	0.0232 (3)	
C6	0.13733 (13)	0.23552 (13)	0.36077 (7)	0.0246 (3)	
H6	0.133657	0.163916	0.350017	0.030*	
C7	0.03343 (13)	0.33081 (13)	0.35836 (7)	0.0247 (3)	
C8	0.03928 (13)	0.43662 (12)	0.37288 (7)	0.0249 (3)	
C9	0.14979 (14)	0.44524 (12)	0.39159 (7)	0.0248 (3)	
C10	0.25432 (13)	0.35051 (13)	0.39515 (7)	0.0243 (3)	
H10	0.329209	0.356513	0.408179	0.029*	
C12	−0.08797 (14)	0.21821 (13)	0.33449 (8)	0.0302 (3)	
H12A	−0.034969	0.184520	0.296552	0.045*	
H12B	−0.172727	0.225907	0.326136	0.045*	
H12C	−0.063494	0.168771	0.376149	0.045*	
C14	−0.14653 (15)	0.54176 (15)	0.42181 (9)	0.0381 (4)	
H14A	−0.214092	0.612816	0.414879	0.057*	
H14B	−0.106492	0.541914	0.463409	0.057*	
H14C	−0.178026	0.476608	0.425514	0.057*	
C16	0.25984 (15)	0.56848 (14)	0.41581 (10)	0.0384 (4)	
H16A	0.315037	0.549050	0.376144	0.058*	
H16B	0.296371	0.519193	0.455526	0.058*	
H16C	0.246036	0.648458	0.423381	0.058*	
C18	0.28998 (13)	−0.02714 (12)	0.38480 (7)	0.0239 (3)	
C19	0.28217 (14)	−0.04663 (13)	0.45531 (7)	0.0268 (3)	
H19	0.335945	−0.026095	0.481531	0.032*	
C20	0.19842 (14)	−0.09485 (14)	0.48762 (8)	0.0292 (3)	
H20	0.195818	−0.107944	0.535467	0.035*	
C21	0.11740 (14)	−0.12452 (13)	0.45020 (8)	0.0276 (3)	
C22	0.12299 (14)	−0.10582 (13)	0.38041 (8)	0.0290 (3)	
H22	0.068320	−0.125593	0.354447	0.035*	
C23	0.20890 (14)	−0.05795 (13)	0.34824 (8)	0.0272 (3)	
H23	0.212225	−0.046069	0.300346	0.033*	
C25	−0.05326 (15)	−0.19440 (15)	0.45198 (8)	0.0346 (4)	
H25A	−0.014348	−0.251659	0.420227	0.052*	
H25B	−0.101539	−0.123914	0.427085	0.052*	
H25C	−0.106692	−0.223322	0.484436	0.052*	
C26	0.38961 (12)	0.04393 (12)	0.27645 (7)	0.0224 (3)	
C27	0.42081 (14)	−0.05596 (12)	0.24303 (7)	0.0267 (3)	
H27	0.431530	−0.127338	0.268706	0.032*	
C28	0.43621 (14)	−0.05264 (13)	0.17374 (7)	0.0275 (3)	
H28	0.457296	−0.121385	0.152212	0.033*	
C29	0.42097 (13)	0.05141 (13)	0.13495 (7)	0.0239 (3)	
C30	0.39408 (13)	0.15089 (12)	0.16707 (7)	0.0247 (3)	
H30	0.386085	0.221892	0.141444	0.030*	
C31	0.37894 (13)	0.14571 (12)	0.23716 (7)	0.0244 (3)	
H31	0.360704	0.214143	0.258770	0.029*	
C33	0.40625 (16)	0.15099 (13)	0.02546 (8)	0.0322 (3)	
H33A	0.416410	0.135899	−0.022220	0.048*	
H33B	0.460695	0.193626	0.034783	0.048*	
H33C	0.321553	0.195969	0.035415	0.048*	
C34	0.62277 (13)	−0.02837 (13)	0.34623 (7)	0.0245 (3)	
C35	0.68780 (14)	−0.14197 (13)	0.33960 (8)	0.0298 (3)	
H35	0.658822	−0.199750	0.363203	0.036*	
C36	0.79423 (15)	−0.17350 (15)	0.29934 (8)	0.0345 (4)	
H36	0.836626	−0.251826	0.294889	0.041*	
C37	0.83797 (15)	−0.08931 (16)	0.26569 (8)	0.0357 (4)	
H37	0.910466	−0.109579	0.237856	0.043*	
C38	0.77509 (15)	0.02458 (15)	0.27298 (8)	0.0346 (4)	
H38	0.805478	0.082329	0.250588	0.042*	
C39	0.66849 (15)	0.05477 (13)	0.31260 (8)	0.0292 (3)	
H39	0.626039	0.133131	0.316881	0.035*	
N1	0.35155 (11)	0.14964 (10)	0.38044 (6)	0.0229 (3)	
O11	−0.07734 (9)	0.32837 (9)	0.34127 (5)	0.0297 (2)	
O13	−0.06042 (9)	0.53295 (9)	0.36581 (6)	0.0303 (2)	
O15	0.14621 (10)	0.55205 (9)	0.40470 (6)	0.0321 (3)	
O17	0.50057 (10)	0.17855 (10)	0.44389 (6)	0.0362 (3)	
O24	0.03835 (10)	−0.17149 (10)	0.48725 (5)	0.0338 (3)	
O32	0.43557 (10)	0.04515 (9)	0.06690 (5)	0.0278 (2)	
C2A	0.53453 (13)	0.41897 (12)	0.09308 (7)	0.0223 (3)	
C3A	0.49183 (13)	0.54262 (12)	0.11030 (7)	0.0224 (3)	
H3A	0.492800	0.596081	0.069084	0.027*	
C4A	0.61774 (13)	0.50903 (12)	0.14866 (7)	0.0216 (3)	
C5A	0.74428 (13)	0.29455 (12)	0.13000 (7)	0.0217 (3)	
C6A	0.85084 (13)	0.30569 (13)	0.15133 (7)	0.0243 (3)	
H6A	0.851693	0.375945	0.165797	0.029*	
C7A	0.95683 (13)	0.21244 (13)	0.15129 (7)	0.0245 (3)	
C8A	0.95452 (13)	0.10805 (12)	0.13221 (7)	0.0247 (3)	
C9A	0.84549 (14)	0.09828 (12)	0.11262 (7)	0.0244 (3)	
C10A	0.73910 (13)	0.19127 (12)	0.11059 (7)	0.0242 (3)	
H10A	0.665261	0.184581	0.096420	0.029*	
C12A	1.07288 (14)	0.32712 (14)	0.17942 (8)	0.0300 (3)	
H12D	1.018780	0.356834	0.217905	0.045*	
H12E	1.047390	0.378997	0.138755	0.045*	
H12F	1.156840	0.321179	0.188543	0.045*	
C14A	1.13658 (15)	0.01136 (15)	0.07683 (9)	0.0368 (4)	
H14D	1.210710	−0.053961	0.082699	0.055*	
H14E	1.158385	0.082093	0.069899	0.055*	
H14F	1.094672	0.003889	0.037458	0.055*	
C16A	0.73881 (15)	−0.02728 (14)	0.08713 (9)	0.0347 (4)	
H16D	0.684824	−0.008542	0.127316	0.052*	
H16E	0.754104	−0.107633	0.079924	0.052*	
H16F	0.700069	0.021189	0.047667	0.052*	
C26A	0.60072 (12)	0.51623 (12)	0.22396 (7)	0.0216 (3)	
C31A	0.61578 (13)	0.42025 (12)	0.27005 (7)	0.0245 (3)	
H31A	0.640381	0.346634	0.253973	0.029*	
C30A	0.59559 (14)	0.43020 (12)	0.33877 (7)	0.0259 (3)	
H30A	0.606527	0.363789	0.369355	0.031*	
C29A	0.55924 (13)	0.53756 (12)	0.36301 (7)	0.0231 (3)	
C28A	0.54078 (13)	0.63421 (12)	0.31783 (7)	0.0249 (3)	
H28A	0.513964	0.707941	0.333806	0.030*	
C27A	0.56175 (14)	0.62245 (12)	0.24916 (7)	0.0250 (3)	
H27A	0.549064	0.689006	0.218549	0.030*	
C33A	0.50372 (15)	0.64958 (13)	0.45788 (7)	0.0292 (3)	
H33D	0.567288	0.686386	0.447766	0.044*	
H33E	0.489437	0.639710	0.506905	0.044*	
H33F	0.428160	0.697423	0.437099	0.044*	
C18A	0.71158 (13)	0.56193 (12)	0.11335 (7)	0.0228 (3)	
C23A	0.78448 (14)	0.60614 (13)	0.14767 (7)	0.0266 (3)	
H23A	0.772010	0.609585	0.195132	0.032*	
C22A	0.87563 (14)	0.64558 (13)	0.11385 (8)	0.0280 (3)	
H22A	0.924198	0.675596	0.138215	0.034*	
C21A	0.89479 (13)	0.64071 (12)	0.04483 (7)	0.0263 (3)	
C20A	0.82355 (14)	0.59507 (13)	0.00985 (7)	0.0272 (3)	
H20A	0.837117	0.590360	−0.037483	0.033*	
C19A	0.73369 (13)	0.55683 (12)	0.04369 (7)	0.0256 (3)	
H19A	0.685758	0.526369	0.019150	0.031*	
C25A	1.06280 (15)	0.71438 (15)	0.04038 (9)	0.0361 (4)	
H25D	1.105247	0.652591	0.073325	0.054*	
H25E	1.018167	0.781856	0.063803	0.054*	
H25F	1.122402	0.733250	0.007567	0.054*	
C34A	0.37341 (13)	0.58151 (12)	0.15049 (7)	0.0239 (3)	
C35A	0.30917 (14)	0.69713 (13)	0.15043 (8)	0.0296 (3)	
H35A	0.338347	0.751051	0.122653	0.036*	
C36A	0.20311 (15)	0.73525 (15)	0.19026 (9)	0.0366 (4)	
H36A	0.161329	0.814660	0.190374	0.044*	
C37A	0.15862 (15)	0.65724 (16)	0.22969 (8)	0.0366 (4)	
H37A	0.086319	0.682890	0.257163	0.044*	
C38A	0.21981 (15)	0.54132 (15)	0.22907 (8)	0.0350 (4)	
H38A	0.188481	0.487557	0.255528	0.042*	
C39A	0.32649 (14)	0.50376 (13)	0.18995 (8)	0.0286 (3)	
H39A	0.368080	0.424281	0.190017	0.034*	
N1A	0.63857 (11)	0.39102 (10)	0.12797 (6)	0.0212 (2)	
O11A	1.06653 (9)	0.21689 (9)	0.16931 (5)	0.0295 (2)	
O13A	1.05774 (10)	0.01439 (9)	0.13582 (5)	0.0299 (2)	
O15A	0.85179 (10)	−0.00808 (9)	0.09658 (6)	0.0296 (2)	
O17A	0.49438 (10)	0.36300 (9)	0.06014 (5)	0.0285 (2)	
O32A	0.54215 (10)	0.54027 (9)	0.43138 (5)	0.0275 (2)	
O24A	0.97899 (10)	0.67854 (10)	0.00599 (5)	0.0330 (3)	

### 4,4-Bis(4-methoxyphenyl)-3-phenyl-1-(3,4,5-trimethoxyphenyl)azetidin-2-one (5) Atomic displacement parameters (Å^2^)

**Table:**

	*U*^11^	*U*^22^	*U*^33^	*U*^12^	*U*^13^	*U*^23^
C2	0.0239 (7)	0.0275 (7)	0.0247 (7)	−0.0054 (6)	−0.0024 (6)	−0.0040 (6)
C3	0.0225 (7)	0.0249 (7)	0.0233 (7)	−0.0066 (6)	−0.0039 (6)	0.0001 (5)
C4	0.0218 (7)	0.0220 (7)	0.0229 (7)	−0.0048 (6)	−0.0018 (5)	−0.0019 (5)
C5	0.0238 (7)	0.0249 (7)	0.0189 (7)	−0.0057 (6)	0.0001 (5)	−0.0004 (5)
C6	0.0256 (8)	0.0255 (7)	0.0232 (7)	−0.0086 (6)	−0.0006 (6)	−0.0020 (6)
C7	0.0215 (7)	0.0311 (8)	0.0213 (7)	−0.0083 (6)	0.0000 (5)	−0.0009 (6)
C8	0.0217 (7)	0.0253 (7)	0.0244 (7)	−0.0043 (6)	0.0009 (6)	0.0007 (6)
C9	0.0257 (8)	0.0233 (7)	0.0248 (7)	−0.0077 (6)	0.0025 (6)	−0.0023 (6)
C10	0.0223 (7)	0.0269 (7)	0.0242 (7)	−0.0090 (6)	0.0012 (6)	−0.0018 (6)
C12	0.0269 (8)	0.0326 (8)	0.0338 (8)	−0.0129 (6)	−0.0023 (6)	−0.0029 (6)
C14	0.0256 (8)	0.0368 (9)	0.0468 (10)	−0.0031 (7)	0.0073 (7)	−0.0079 (7)
C16	0.0302 (9)	0.0282 (8)	0.0592 (11)	−0.0113 (7)	−0.0036 (8)	−0.0059 (7)
C18	0.0232 (7)	0.0218 (7)	0.0251 (7)	−0.0044 (6)	−0.0008 (6)	−0.0024 (5)
C19	0.0244 (8)	0.0296 (8)	0.0256 (7)	−0.0072 (6)	−0.0023 (6)	−0.0020 (6)
C20	0.0292 (8)	0.0341 (8)	0.0238 (7)	−0.0097 (6)	0.0012 (6)	−0.0021 (6)
C21	0.0262 (8)	0.0262 (7)	0.0305 (8)	−0.0090 (6)	0.0021 (6)	−0.0022 (6)
C22	0.0298 (8)	0.0289 (8)	0.0304 (8)	−0.0111 (6)	−0.0021 (6)	−0.0047 (6)
C23	0.0290 (8)	0.0278 (7)	0.0249 (7)	−0.0089 (6)	−0.0012 (6)	−0.0028 (6)
C25	0.0307 (9)	0.0407 (9)	0.0374 (9)	−0.0180 (7)	0.0053 (7)	−0.0089 (7)
C26	0.0178 (7)	0.0247 (7)	0.0240 (7)	−0.0058 (5)	−0.0019 (5)	−0.0014 (6)
C27	0.0297 (8)	0.0215 (7)	0.0272 (7)	−0.0054 (6)	−0.0022 (6)	−0.0015 (6)
C28	0.0309 (8)	0.0228 (7)	0.0277 (7)	−0.0062 (6)	0.0000 (6)	−0.0045 (6)
C29	0.0207 (7)	0.0282 (7)	0.0228 (7)	−0.0077 (6)	0.0000 (5)	−0.0031 (6)
C30	0.0255 (7)	0.0219 (7)	0.0266 (7)	−0.0082 (6)	−0.0015 (6)	−0.0001 (6)
C31	0.0257 (7)	0.0227 (7)	0.0256 (7)	−0.0076 (6)	−0.0009 (6)	−0.0047 (6)
C33	0.0455 (10)	0.0272 (8)	0.0244 (7)	−0.0126 (7)	−0.0032 (7)	0.0002 (6)
C34	0.0234 (7)	0.0269 (7)	0.0237 (7)	−0.0079 (6)	−0.0032 (6)	−0.0020 (6)
C35	0.0268 (8)	0.0267 (8)	0.0354 (8)	−0.0086 (6)	−0.0009 (6)	−0.0002 (6)
C36	0.0264 (8)	0.0350 (9)	0.0389 (9)	−0.0040 (7)	−0.0012 (7)	−0.0073 (7)
C37	0.0265 (8)	0.0512 (10)	0.0289 (8)	−0.0114 (7)	0.0024 (6)	−0.0061 (7)
C38	0.0361 (9)	0.0446 (9)	0.0274 (8)	−0.0204 (8)	0.0011 (7)	0.0009 (7)
C39	0.0323 (8)	0.0288 (8)	0.0277 (7)	−0.0114 (6)	−0.0025 (6)	−0.0012 (6)
N1	0.0227 (6)	0.0227 (6)	0.0233 (6)	−0.0061 (5)	−0.0017 (5)	−0.0036 (5)
O11	0.0220 (5)	0.0307 (6)	0.0364 (6)	−0.0073 (4)	−0.0049 (4)	−0.0032 (4)
O13	0.0224 (5)	0.0277 (6)	0.0359 (6)	−0.0023 (4)	0.0010 (4)	0.0003 (4)
O15	0.0260 (6)	0.0236 (5)	0.0470 (7)	−0.0071 (4)	−0.0020 (5)	−0.0061 (5)
O17	0.0315 (6)	0.0352 (6)	0.0413 (6)	−0.0032 (5)	−0.0128 (5)	−0.0143 (5)
O24	0.0328 (6)	0.0420 (7)	0.0313 (6)	−0.0195 (5)	0.0042 (5)	−0.0028 (5)
O32	0.0361 (6)	0.0258 (5)	0.0216 (5)	−0.0100 (4)	0.0004 (4)	−0.0021 (4)
C2A	0.0220 (7)	0.0256 (7)	0.0191 (6)	−0.0077 (6)	−0.0001 (5)	−0.0004 (5)
C3A	0.0224 (7)	0.0237 (7)	0.0210 (7)	−0.0073 (6)	−0.0018 (5)	−0.0001 (5)
C4A	0.0218 (7)	0.0198 (7)	0.0221 (7)	−0.0049 (5)	−0.0014 (5)	−0.0022 (5)
C5A	0.0226 (7)	0.0236 (7)	0.0173 (6)	−0.0060 (6)	0.0009 (5)	0.0002 (5)
C6A	0.0262 (8)	0.0260 (7)	0.0204 (7)	−0.0075 (6)	−0.0004 (6)	−0.0027 (5)
C7A	0.0222 (7)	0.0288 (7)	0.0219 (7)	−0.0079 (6)	0.0004 (5)	−0.0005 (6)
C8A	0.0223 (7)	0.0255 (7)	0.0223 (7)	−0.0030 (6)	0.0021 (6)	−0.0005 (6)
C9A	0.0270 (8)	0.0244 (7)	0.0210 (7)	−0.0076 (6)	0.0022 (6)	−0.0017 (5)
C10A	0.0236 (7)	0.0259 (7)	0.0226 (7)	−0.0076 (6)	0.0012 (6)	−0.0020 (6)
C12A	0.0252 (8)	0.0345 (8)	0.0324 (8)	−0.0116 (6)	−0.0027 (6)	−0.0034 (6)
C14A	0.0262 (8)	0.0363 (9)	0.0417 (9)	−0.0028 (7)	0.0084 (7)	−0.0045 (7)
C16A	0.0305 (9)	0.0277 (8)	0.0475 (10)	−0.0103 (7)	−0.0014 (7)	−0.0068 (7)
C26A	0.0179 (7)	0.0258 (7)	0.0216 (7)	−0.0077 (5)	−0.0010 (5)	−0.0017 (5)
C31A	0.0245 (7)	0.0218 (7)	0.0261 (7)	−0.0063 (6)	0.0011 (6)	−0.0020 (6)
C30A	0.0286 (8)	0.0237 (7)	0.0235 (7)	−0.0073 (6)	0.0003 (6)	0.0017 (6)
C29A	0.0217 (7)	0.0274 (7)	0.0208 (7)	−0.0086 (6)	0.0012 (5)	−0.0032 (6)
C28A	0.0278 (8)	0.0226 (7)	0.0251 (7)	−0.0084 (6)	−0.0009 (6)	−0.0045 (6)
C27A	0.0289 (8)	0.0229 (7)	0.0233 (7)	−0.0087 (6)	−0.0014 (6)	0.0002 (6)
C33A	0.0375 (9)	0.0279 (8)	0.0245 (7)	−0.0131 (7)	0.0043 (6)	−0.0070 (6)
C18A	0.0222 (7)	0.0208 (7)	0.0243 (7)	−0.0056 (5)	−0.0004 (6)	−0.0006 (5)
C23A	0.0289 (8)	0.0294 (8)	0.0226 (7)	−0.0107 (6)	−0.0004 (6)	−0.0020 (6)
C22A	0.0292 (8)	0.0286 (8)	0.0292 (8)	−0.0134 (6)	−0.0019 (6)	−0.0014 (6)
C21A	0.0222 (7)	0.0259 (7)	0.0289 (7)	−0.0068 (6)	0.0015 (6)	0.0023 (6)
C20A	0.0264 (8)	0.0307 (8)	0.0227 (7)	−0.0073 (6)	0.0005 (6)	−0.0001 (6)
C19A	0.0254 (8)	0.0270 (7)	0.0238 (7)	−0.0073 (6)	−0.0022 (6)	−0.0010 (6)
C25A	0.0316 (9)	0.0407 (9)	0.0410 (9)	−0.0203 (7)	0.0042 (7)	−0.0018 (7)
C34A	0.0219 (7)	0.0266 (7)	0.0226 (7)	−0.0063 (6)	−0.0029 (6)	−0.0029 (6)
C35A	0.0257 (8)	0.0277 (8)	0.0342 (8)	−0.0069 (6)	−0.0009 (6)	−0.0018 (6)
C36A	0.0303 (9)	0.0331 (9)	0.0411 (9)	−0.0011 (7)	−0.0009 (7)	−0.0068 (7)
C37A	0.0248 (8)	0.0485 (10)	0.0320 (8)	−0.0047 (7)	0.0049 (6)	−0.0081 (7)
C38A	0.0320 (9)	0.0433 (9)	0.0301 (8)	−0.0146 (7)	0.0046 (7)	−0.0001 (7)
C39A	0.0280 (8)	0.0279 (8)	0.0291 (8)	−0.0083 (6)	0.0010 (6)	−0.0017 (6)
N1A	0.0206 (6)	0.0211 (6)	0.0216 (6)	−0.0056 (5)	−0.0005 (4)	−0.0033 (4)
O11A	0.0222 (5)	0.0310 (6)	0.0350 (6)	−0.0067 (4)	−0.0049 (4)	−0.0030 (4)
O13A	0.0231 (5)	0.0274 (5)	0.0329 (6)	−0.0007 (4)	0.0023 (4)	0.0002 (4)
O15A	0.0273 (6)	0.0230 (5)	0.0380 (6)	−0.0062 (4)	0.0008 (5)	−0.0065 (4)
O17A	0.0287 (6)	0.0288 (5)	0.0296 (5)	−0.0091 (4)	−0.0074 (4)	−0.0054 (4)
O32A	0.0371 (6)	0.0257 (5)	0.0195 (5)	−0.0103 (4)	0.0031 (4)	−0.0030 (4)
O24A	0.0302 (6)	0.0403 (6)	0.0309 (6)	−0.0170 (5)	0.0045 (5)	0.0013 (5)

### 4,4-Bis(4-methoxyphenyl)-3-phenyl-1-(3,4,5-trimethoxyphenyl)azetidin-2-one (5) Geometric parameters (Å, º)

**Table:**

C2—C3	1.530 (2)	C2A—C3A	1.5240 (19)
C2—N1	1.3600 (19)	C2A—N1A	1.3643 (18)
C2—O17	1.2127 (18)	C2A—O17A	1.2152 (17)
C3—H3	1.0000	C3A—H3A	1.0000
C3—C4	1.6122 (19)	C3A—C4A	1.6105 (19)
C3—C34	1.505 (2)	C3A—C34A	1.5017 (19)
C4—C18	1.5299 (19)	C4A—C26A	1.5080 (18)
C4—C26	1.5119 (19)	C4A—C18A	1.5304 (18)
C4—N1	1.4996 (18)	C4A—N1A	1.5042 (17)
C5—C6	1.382 (2)	C5A—C6A	1.385 (2)
C5—C10	1.399 (2)	C5A—C10A	1.397 (2)
C5—N1	1.4164 (18)	C5A—N1A	1.4142 (18)
C6—H6	0.9500	C6A—H6A	0.9500
C6—C7	1.393 (2)	C6A—C7A	1.394 (2)
C7—C8	1.397 (2)	C7A—C8A	1.397 (2)
C7—O11	1.3608 (17)	C7A—O11A	1.3637 (18)
C8—C9	1.397 (2)	C8A—C9A	1.392 (2)
C8—O13	1.3760 (17)	C8A—O13A	1.3776 (18)
C9—C10	1.394 (2)	C9A—C10A	1.395 (2)
C9—O15	1.3649 (18)	C9A—O15A	1.3689 (18)
C10—H10	0.9500	C10A—H10A	0.9500
C12—H12A	0.9800	C12A—H12D	0.9800
C12—H12B	0.9800	C12A—H12E	0.9800
C12—H12C	0.9800	C12A—H12F	0.9800
C12—O11	1.4310 (18)	C12A—O11A	1.4282 (19)
C14—H14A	0.9800	C14A—H14D	0.9800
C14—H14B	0.9800	C14A—H14E	0.9800
C14—H14C	0.9800	C14A—H14F	0.9800
C14—O13	1.4294 (19)	C14A—O13A	1.4303 (19)
C16—H16A	0.9800	C16A—H16D	0.9800
C16—H16B	0.9800	C16A—H16E	0.9800
C16—H16C	0.9800	C16A—H16F	0.9800
C16—O15	1.4290 (19)	C16A—O15A	1.4299 (19)
C18—C19	1.401 (2)	C26A—C31A	1.395 (2)
C18—C23	1.395 (2)	C26A—C27A	1.387 (2)
C19—H19	0.9500	C31A—H31A	0.9500
C19—C20	1.379 (2)	C31A—C30A	1.385 (2)
C20—H20	0.9500	C30A—H30A	0.9500
C20—C21	1.397 (2)	C30A—C29A	1.392 (2)
C21—C22	1.386 (2)	C29A—C28A	1.388 (2)
C21—O24	1.3657 (18)	C29A—O32A	1.3652 (16)
C22—H22	0.9500	C28A—H28A	0.9500
C22—C23	1.396 (2)	C28A—C27A	1.388 (2)
C23—H23	0.9500	C27A—H27A	0.9500
C25—H25A	0.9800	C33A—H33D	0.9800
C25—H25B	0.9800	C33A—H33E	0.9800
C25—H25C	0.9800	C33A—H33F	0.9800
C25—O24	1.4294 (19)	C33A—O32A	1.4329 (17)
C26—C27	1.403 (2)	C18A—C23A	1.393 (2)
C26—C31	1.387 (2)	C18A—C19A	1.398 (2)
C27—H27	0.9500	C23A—H23A	0.9500
C27—C28	1.377 (2)	C23A—C22A	1.398 (2)
C28—H28	0.9500	C22A—H22A	0.9500
C28—C29	1.398 (2)	C22A—C21A	1.382 (2)
C29—C30	1.388 (2)	C21A—C20A	1.397 (2)
C29—O32	1.3621 (17)	C21A—O24A	1.3665 (17)
C30—H30	0.9500	C20A—H20A	0.9500
C30—C31	1.392 (2)	C20A—C19A	1.378 (2)
C31—H31	0.9500	C19A—H19A	0.9500
C33—H33A	0.9800	C25A—H25D	0.9800
C33—H33B	0.9800	C25A—H25E	0.9800
C33—H33C	0.9800	C25A—H25F	0.9800
C33—O32	1.4299 (18)	C25A—O24A	1.4302 (19)
C34—C35	1.387 (2)	C34A—C35A	1.390 (2)
C34—C39	1.391 (2)	C34A—C39A	1.395 (2)
C35—H35	0.9500	C35A—H35A	0.9500
C35—C36	1.390 (2)	C35A—C36A	1.389 (2)
C36—H36	0.9500	C36A—H36A	0.9500
C36—C37	1.389 (2)	C36A—C37A	1.382 (2)
C37—H37	0.9500	C37A—H37A	0.9500
C37—C38	1.387 (3)	C37A—C38A	1.388 (2)
C38—H38	0.9500	C38A—H38A	0.9500
C38—C39	1.383 (2)	C38A—C39A	1.386 (2)
C39—H39	0.9500	C39A—H39A	0.9500
			
N1—C2—C3	93.66 (11)	N1A—C2A—C3A	93.91 (11)
O17—C2—C3	134.62 (14)	O17A—C2A—C3A	134.56 (13)
O17—C2—N1	131.71 (14)	O17A—C2A—N1A	131.53 (14)
C2—C3—H3	110.9	C2A—C3A—H3A	111.1
C2—C3—C4	85.08 (10)	C2A—C3A—C4A	85.24 (10)
C4—C3—H3	110.9	C4A—C3A—H3A	111.1
C34—C3—C2	117.03 (12)	C34A—C3A—C2A	116.99 (11)
C34—C3—H3	110.9	C34A—C3A—H3A	111.1
C34—C3—C4	119.75 (11)	C34A—C3A—C4A	118.91 (11)
C18—C4—C3	114.51 (11)	C26A—C4A—C3A	113.84 (11)
C26—C4—C3	113.35 (11)	C26A—C4A—C18A	115.44 (11)
C26—C4—C18	115.70 (12)	C18A—C4A—C3A	113.97 (11)
N1—C4—C3	85.36 (10)	N1A—C4A—C3A	85.39 (10)
N1—C4—C18	111.32 (11)	N1A—C4A—C26A	113.61 (11)
N1—C4—C26	112.88 (11)	N1A—C4A—C18A	110.97 (11)
C6—C5—C10	121.83 (14)	C6A—C5A—C10A	121.79 (13)
C6—C5—N1	119.03 (13)	C6A—C5A—N1A	118.52 (13)
C10—C5—N1	119.13 (13)	C10A—C5A—N1A	119.70 (13)
C5—C6—H6	120.4	C5A—C6A—H6A	120.4
C5—C6—C7	119.24 (13)	C5A—C6A—C7A	119.14 (13)
C7—C6—H6	120.4	C7A—C6A—H6A	120.4
C6—C7—C8	120.42 (13)	C6A—C7A—C8A	120.37 (13)
O11—C7—C6	123.47 (13)	O11A—C7A—C6A	123.07 (13)
O11—C7—C8	116.11 (13)	O11A—C7A—C8A	116.55 (13)
C7—C8—C9	119.25 (13)	C9A—C8A—C7A	119.34 (13)
O13—C8—C7	120.89 (13)	O13A—C8A—C7A	120.22 (13)
O13—C8—C9	119.79 (13)	O13A—C8A—C9A	120.37 (13)
C10—C9—C8	121.14 (13)	C8A—C9A—C10A	121.25 (14)
O15—C9—C8	114.82 (13)	O15A—C9A—C8A	114.95 (13)
O15—C9—C10	124.04 (13)	O15A—C9A—C10A	123.80 (13)
C5—C10—H10	120.9	C5A—C10A—H10A	121.0
C9—C10—C5	118.11 (13)	C9A—C10A—C5A	118.07 (13)
C9—C10—H10	120.9	C9A—C10A—H10A	121.0
H12A—C12—H12B	109.5	H12D—C12A—H12E	109.5
H12A—C12—H12C	109.5	H12D—C12A—H12F	109.5
H12B—C12—H12C	109.5	H12E—C12A—H12F	109.5
O11—C12—H12A	109.5	O11A—C12A—H12D	109.5
O11—C12—H12B	109.5	O11A—C12A—H12E	109.5
O11—C12—H12C	109.5	O11A—C12A—H12F	109.5
H14A—C14—H14B	109.5	H14D—C14A—H14E	109.5
H14A—C14—H14C	109.5	H14D—C14A—H14F	109.5
H14B—C14—H14C	109.5	H14E—C14A—H14F	109.5
O13—C14—H14A	109.5	O13A—C14A—H14D	109.5
O13—C14—H14B	109.5	O13A—C14A—H14E	109.5
O13—C14—H14C	109.5	O13A—C14A—H14F	109.5
H16A—C16—H16B	109.5	H16D—C16A—H16E	109.5
H16A—C16—H16C	109.5	H16D—C16A—H16F	109.5
H16B—C16—H16C	109.5	H16E—C16A—H16F	109.5
O15—C16—H16A	109.5	O15A—C16A—H16D	109.5
O15—C16—H16B	109.5	O15A—C16A—H16E	109.5
O15—C16—H16C	109.5	O15A—C16A—H16F	109.5
C19—C18—C4	118.84 (13)	C31A—C26A—C4A	122.78 (12)
C23—C18—C4	123.58 (13)	C27A—C26A—C4A	119.41 (12)
C23—C18—C19	117.31 (13)	C27A—C26A—C31A	117.71 (13)
C18—C19—H19	119.2	C26A—C31A—H31A	119.4
C20—C19—C18	121.69 (14)	C30A—C31A—C26A	121.29 (13)
C20—C19—H19	119.2	C30A—C31A—H31A	119.4
C19—C20—H20	119.9	C31A—C30A—H30A	120.0
C19—C20—C21	120.22 (14)	C31A—C30A—C29A	119.96 (13)
C21—C20—H20	119.9	C29A—C30A—H30A	120.0
C22—C21—C20	119.23 (14)	C28A—C29A—C30A	119.59 (13)
O24—C21—C20	115.35 (13)	O32A—C29A—C30A	116.48 (12)
O24—C21—C22	125.42 (14)	O32A—C29A—C28A	123.92 (13)
C21—C22—H22	120.0	C29A—C28A—H28A	120.2
C21—C22—C23	120.04 (14)	C29A—C28A—C27A	119.57 (13)
C23—C22—H22	120.0	C27A—C28A—H28A	120.2
C18—C23—C22	121.51 (14)	C26A—C27A—C28A	121.85 (13)
C18—C23—H23	119.2	C26A—C27A—H27A	119.1
C22—C23—H23	119.2	C28A—C27A—H27A	119.1
H25A—C25—H25B	109.5	H33D—C33A—H33E	109.5
H25A—C25—H25C	109.5	H33D—C33A—H33F	109.5
H25B—C25—H25C	109.5	H33E—C33A—H33F	109.5
O24—C25—H25A	109.5	O32A—C33A—H33D	109.5
O24—C25—H25B	109.5	O32A—C33A—H33E	109.5
O24—C25—H25C	109.5	O32A—C33A—H33F	109.5
C27—C26—C4	119.73 (12)	C23A—C18A—C4A	123.46 (12)
C31—C26—C4	122.80 (13)	C23A—C18A—C19A	117.62 (13)
C31—C26—C27	117.35 (13)	C19A—C18A—C4A	118.67 (12)
C26—C27—H27	119.3	C18A—C23A—H23A	119.3
C28—C27—C26	121.34 (13)	C18A—C23A—C22A	121.49 (13)
C28—C27—H27	119.3	C22A—C23A—H23A	119.3
C27—C28—H28	119.8	C23A—C22A—H22A	120.1
C27—C28—C29	120.31 (13)	C21A—C22A—C23A	119.75 (14)
C29—C28—H28	119.8	C21A—C22A—H22A	120.1
C30—C29—C28	119.35 (13)	C22A—C21A—C20A	119.42 (13)
O32—C29—C28	115.42 (13)	O24A—C21A—C22A	125.27 (14)
O32—C29—C30	125.22 (13)	O24A—C21A—C20A	115.31 (13)
C29—C30—H30	120.3	C21A—C20A—H20A	119.8
C29—C30—C31	119.43 (13)	C19A—C20A—C21A	120.31 (14)
C31—C30—H30	120.3	C19A—C20A—H20A	119.8
C26—C31—C30	122.15 (13)	C18A—C19A—H19A	119.3
C26—C31—H31	118.9	C20A—C19A—C18A	121.41 (14)
C30—C31—H31	118.9	C20A—C19A—H19A	119.3
H33A—C33—H33B	109.5	H25D—C25A—H25E	109.5
H33A—C33—H33C	109.5	H25D—C25A—H25F	109.5
H33B—C33—H33C	109.5	H25E—C25A—H25F	109.5
O32—C33—H33A	109.5	O24A—C25A—H25D	109.5
O32—C33—H33B	109.5	O24A—C25A—H25E	109.5
O32—C33—H33C	109.5	O24A—C25A—H25F	109.5
C35—C34—C3	120.19 (13)	C35A—C34A—C3A	120.34 (13)
C35—C34—C39	118.17 (14)	C35A—C34A—C39A	118.31 (14)
C39—C34—C3	121.64 (14)	C39A—C34A—C3A	121.34 (13)
C34—C35—H35	119.1	C34A—C35A—H35A	119.4
C34—C35—C36	121.72 (15)	C36A—C35A—C34A	121.21 (15)
C36—C35—H35	119.1	C36A—C35A—H35A	119.4
C35—C36—H36	120.4	C35A—C36A—H36A	120.1
C37—C36—C35	119.30 (16)	C37A—C36A—C35A	119.76 (16)
C37—C36—H36	120.4	C37A—C36A—H36A	120.1
C36—C37—H37	120.2	C36A—C37A—H37A	120.1
C38—C37—C36	119.54 (15)	C36A—C37A—C38A	119.83 (15)
C38—C37—H37	120.2	C38A—C37A—H37A	120.1
C37—C38—H38	119.7	C37A—C38A—H38A	119.9
C39—C38—C37	120.54 (15)	C39A—C38A—C37A	120.20 (15)
C39—C38—H38	119.7	C39A—C38A—H38A	119.9
C34—C39—H39	119.6	C34A—C39A—H39A	119.7
C38—C39—C34	120.72 (15)	C38A—C39A—C34A	120.65 (15)
C38—C39—H39	119.6	C38A—C39A—H39A	119.7
C2—N1—C4	95.90 (11)	C2A—N1A—C4A	95.38 (11)
C2—N1—C5	132.98 (12)	C2A—N1A—C5A	132.40 (12)
C5—N1—C4	130.96 (12)	C5A—N1A—C4A	130.49 (11)
C7—O11—C12	116.43 (12)	C7A—O11A—C12A	116.42 (12)
C8—O13—C14	112.87 (12)	C8A—O13A—C14A	112.31 (12)
C9—O15—C16	116.94 (12)	C9A—O15A—C16A	116.85 (12)
C21—O24—C25	117.60 (12)	C29A—O32A—C33A	117.84 (11)
C29—O32—C33	116.79 (11)	C21A—O24A—C25A	117.25 (12)
			
C2—C3—C4—C18	−111.46 (12)	C2A—C3A—C4A—C26A	−115.74 (12)
C2—C3—C4—C26	112.82 (12)	C2A—C3A—C4A—C18A	108.99 (12)
C2—C3—C4—N1	−0.12 (9)	C2A—C3A—C4A—N1A	−1.93 (9)
C2—C3—C34—C35	162.12 (14)	C2A—C3A—C34A—C35A	−159.75 (13)
C2—C3—C34—C39	−18.3 (2)	C2A—C3A—C34A—C39A	21.86 (19)
C3—C2—N1—C4	−0.15 (11)	C3A—C2A—N1A—C4A	−2.28 (11)
C3—C2—N1—C5	175.38 (14)	C3A—C2A—N1A—C5A	−168.04 (14)
C3—C4—C18—C19	39.59 (18)	C3A—C4A—C26A—C31A	101.97 (15)
C3—C4—C18—C23	−146.56 (14)	C3A—C4A—C26A—C27A	−74.25 (16)
C3—C4—C26—C27	81.23 (16)	C3A—C4A—C18A—C23A	139.46 (14)
C3—C4—C26—C31	−94.68 (16)	C3A—C4A—C18A—C19A	−46.49 (17)
C3—C4—N1—C2	0.14 (11)	C3A—C4A—N1A—C2A	2.16 (10)
C3—C4—N1—C5	−175.52 (14)	C3A—C4A—N1A—C5A	168.34 (13)
C3—C34—C35—C36	178.00 (14)	C3A—C34A—C35A—C36A	−176.29 (14)
C3—C34—C39—C38	−178.78 (14)	C3A—C34A—C39A—C38A	177.17 (14)
C4—C3—C34—C35	−97.47 (17)	C4A—C3A—C34A—C35A	100.18 (16)
C4—C3—C34—C39	82.12 (17)	C4A—C3A—C34A—C39A	−78.21 (17)
C4—C18—C19—C20	174.58 (14)	C4A—C26A—C31A—C30A	−177.88 (13)
C4—C18—C23—C22	−173.67 (14)	C4A—C26A—C27A—C28A	177.88 (13)
C4—C26—C27—C28	−178.27 (13)	C4A—C18A—C23A—C22A	174.94 (13)
C4—C26—C31—C30	178.18 (13)	C4A—C18A—C19A—C20A	−174.99 (13)
C5—C6—C7—C8	1.3 (2)	C5A—C6A—C7A—C8A	−2.2 (2)
C5—C6—C7—O11	−179.75 (12)	C5A—C6A—C7A—O11A	177.77 (12)
C6—C5—C10—C9	−0.9 (2)	C6A—C5A—C10A—C9A	−0.5 (2)
C6—C5—N1—C2	−160.61 (14)	C6A—C5A—N1A—C2A	158.07 (14)
C6—C5—N1—C4	13.5 (2)	C6A—C5A—N1A—C4A	−3.1 (2)
C6—C7—C8—C9	−1.7 (2)	C6A—C7A—C8A—C9A	0.6 (2)
C6—C7—C8—O13	175.14 (13)	C6A—C7A—C8A—O13A	−176.38 (12)
C6—C7—O11—C12	8.4 (2)	C6A—C7A—O11A—C12A	−8.64 (19)
C7—C8—C9—C10	0.8 (2)	C7A—C8A—C9A—C10A	1.1 (2)
C7—C8—C9—O15	179.97 (13)	C7A—C8A—C9A—O15A	−178.35 (12)
C7—C8—O13—C14	80.37 (17)	C7A—C8A—O13A—C14A	−84.95 (17)
C8—C7—O11—C12	−172.52 (13)	C8A—C7A—O11A—C12A	171.34 (13)
C8—C9—C10—C5	0.4 (2)	C8A—C9A—C10A—C5A	−1.1 (2)
C8—C9—O15—C16	−172.44 (13)	C8A—C9A—O15A—C16A	169.90 (13)
C9—C8—O13—C14	−102.83 (16)	C9A—C8A—O13A—C14A	98.10 (16)
C10—C5—C6—C7	0.0 (2)	C10A—C5A—C6A—C7A	2.2 (2)
C10—C5—N1—C2	20.2 (2)	C10A—C5A—N1A—C2A	−21.8 (2)
C10—C5—N1—C4	−165.69 (13)	C10A—C5A—N1A—C4A	176.95 (12)
C10—C9—O15—C16	6.7 (2)	C10A—C9A—O15A—C16A	−9.5 (2)
C18—C4—C26—C27	−53.94 (18)	C26A—C4A—C18A—C23A	4.9 (2)
C18—C4—C26—C31	130.15 (14)	C26A—C4A—C18A—C19A	178.98 (13)
C18—C4—N1—C2	114.66 (12)	C26A—C4A—N1A—C2A	116.19 (12)
C18—C4—N1—C5	−61.00 (18)	C26A—C4A—N1A—C5A	−77.63 (17)
C18—C19—C20—C21	−0.8 (2)	C26A—C31A—C30A—C29A	0.1 (2)
C19—C18—C23—C22	0.3 (2)	C31A—C26A—C27A—C28A	1.5 (2)
C19—C20—C21—C22	0.5 (2)	C31A—C30A—C29A—C28A	1.5 (2)
C19—C20—C21—O24	−179.92 (14)	C31A—C30A—C29A—O32A	−179.47 (13)
C20—C21—C22—C23	0.1 (2)	C30A—C29A—C28A—C27A	−1.6 (2)
C20—C21—O24—C25	174.79 (14)	C30A—C29A—O32A—C33A	−179.49 (13)
C21—C22—C23—C18	−0.5 (2)	C29A—C28A—C27A—C26A	0.1 (2)
C22—C21—O24—C25	−5.7 (2)	C28A—C29A—O32A—C33A	−0.5 (2)
C23—C18—C19—C20	0.4 (2)	C27A—C26A—C31A—C30A	−1.6 (2)
C26—C4—C18—C19	174.24 (13)	C18A—C4A—C26A—C31A	−123.44 (14)
C26—C4—C18—C23	−11.9 (2)	C18A—C4A—C26A—C27A	60.34 (17)
C26—C4—N1—C2	−113.27 (13)	C18A—C4A—N1A—C2A	−111.77 (12)
C26—C4—N1—C5	71.07 (18)	C18A—C4A—N1A—C5A	54.41 (18)
C26—C27—C28—C29	0.0 (2)	C18A—C23A—C22A—C21A	−0.2 (2)
C27—C26—C31—C30	2.2 (2)	C23A—C18A—C19A—C20A	−0.6 (2)
C27—C28—C29—C30	2.1 (2)	C23A—C22A—C21A—C20A	−0.8 (2)
C27—C28—C29—O32	−178.53 (13)	C23A—C22A—C21A—O24A	178.60 (14)
C28—C29—C30—C31	−2.0 (2)	C22A—C21A—C20A—C19A	1.0 (2)
C28—C29—O32—C33	172.66 (13)	C22A—C21A—O24A—C25A	7.3 (2)
C29—C30—C31—C26	−0.1 (2)	C21A—C20A—C19A—C18A	−0.3 (2)
C30—C29—O32—C33	−8.0 (2)	C20A—C21A—O24A—C25A	−173.35 (14)
C31—C26—C27—C28	−2.1 (2)	C19A—C18A—C23A—C22A	0.8 (2)
C34—C3—C4—C18	130.10 (13)	C34A—C3A—C4A—C26A	2.56 (17)
C34—C3—C4—C26	−5.61 (18)	C34A—C3A—C4A—C18A	−132.71 (13)
C34—C3—C4—N1	−118.56 (13)	C34A—C3A—C4A—N1A	116.37 (12)
C34—C35—C36—C37	1.1 (2)	C34A—C35A—C36A—C37A	−1.4 (2)
C35—C34—C39—C38	0.8 (2)	C35A—C34A—C39A—C38A	−1.3 (2)
C35—C36—C37—C38	0.2 (2)	C35A—C36A—C37A—C38A	−0.3 (3)
C36—C37—C38—C39	−1.0 (2)	C36A—C37A—C38A—C39A	1.1 (3)
C37—C38—C39—C34	0.5 (2)	C37A—C38A—C39A—C34A	−0.4 (2)
C39—C34—C35—C36	−1.6 (2)	C39A—C34A—C35A—C36A	2.1 (2)
N1—C2—C3—C4	0.14 (10)	N1A—C2A—C3A—C4A	2.13 (10)
N1—C2—C3—C34	121.15 (13)	N1A—C2A—C3A—C34A	−118.01 (12)
N1—C4—C18—C19	−55.13 (17)	N1A—C4A—C26A—C31A	6.39 (19)
N1—C4—C18—C23	118.71 (15)	N1A—C4A—C26A—C27A	−169.83 (12)
N1—C4—C26—C27	176.19 (12)	N1A—C4A—C18A—C23A	−126.17 (14)
N1—C4—C26—C31	0.28 (19)	N1A—C4A—C18A—C19A	47.89 (17)
N1—C5—C6—C7	−179.12 (12)	N1A—C5A—C6A—C7A	−177.72 (12)
N1—C5—C10—C9	178.29 (12)	N1A—C5A—C10A—C9A	179.37 (12)
O11—C7—C8—C9	179.26 (12)	O11A—C7A—C8A—C9A	−179.38 (12)
O11—C7—C8—O13	−3.93 (19)	O11A—C7A—C8A—O13A	3.65 (19)
O13—C8—C9—C10	−176.03 (12)	O13A—C8A—C9A—C10A	178.07 (12)
O13—C8—C9—O15	3.12 (19)	O13A—C8A—C9A—O15A	−1.38 (19)
O15—C9—C10—C5	−178.64 (13)	O15A—C9A—C10A—C5A	178.26 (12)
O17—C2—C3—C4	179.89 (18)	O17A—C2A—C3A—C4A	−177.17 (16)
O17—C2—C3—C34	−59.1 (2)	O17A—C2A—C3A—C34A	62.7 (2)
O17—C2—N1—C4	−179.91 (17)	O17A—C2A—N1A—C4A	177.05 (15)
O17—C2—N1—C5	−4.4 (3)	O17A—C2A—N1A—C5A	11.3 (3)
O24—C21—C22—C23	−179.42 (14)	O32A—C29A—C28A—C27A	179.42 (13)
O32—C29—C30—C31	178.64 (13)	O24A—C21A—C20A—C19A	−178.42 (13)

### 4,4-Bis(4-methoxyphenyl)-3-phenyl-1-(3,4,5-trimethoxyphenyl)azetidin-2-one (5) Hydrogen-bond geometry (Å, º)

**Table:**

*D*—H···*A*	*D*—H	H···*A*	*D*···*A*	*D*—H···*A*
C10—H10···O17	0.95	2.53	3.1190 (19)	120
C19—H19···O17^i^	0.95	2.63	3.2357 (18)	122
C33—H33*A*···O32^ii^	0.98	2.56	3.2110 (19)	124
C33—H33*B*···O17*A*	0.98	2.37	3.2593 (18)	151
C10*A*—H10*A*···O17*A*	0.95	2.53	3.1158 (18)	120
C33*A*—H33*E*···O32*A*^iii^	0.98	2.55	3.1918 (18)	123

Symmetry codes: (i) −*x*+1, −*y*, −*z*+1; (ii) −*x*+1, −*y*, −*z*; (iii) −*x*+1, −*y*+1, −*z*+1.
