# Supplementary figures and images for: Azetidin-2-ones: structures of anti­mitotic compounds based on the 1-(3,4,5-tri­meth­oxy­phen­yl)azetidin-2-one core
Source: Acta Crystallogr E Crystallogr Commun. 2020 Jul 3;76(Pt 8):1187–94. doi: 10.1107/S2056989020008555 (PMC7405576; doi:10.1107/S2056989020008555)

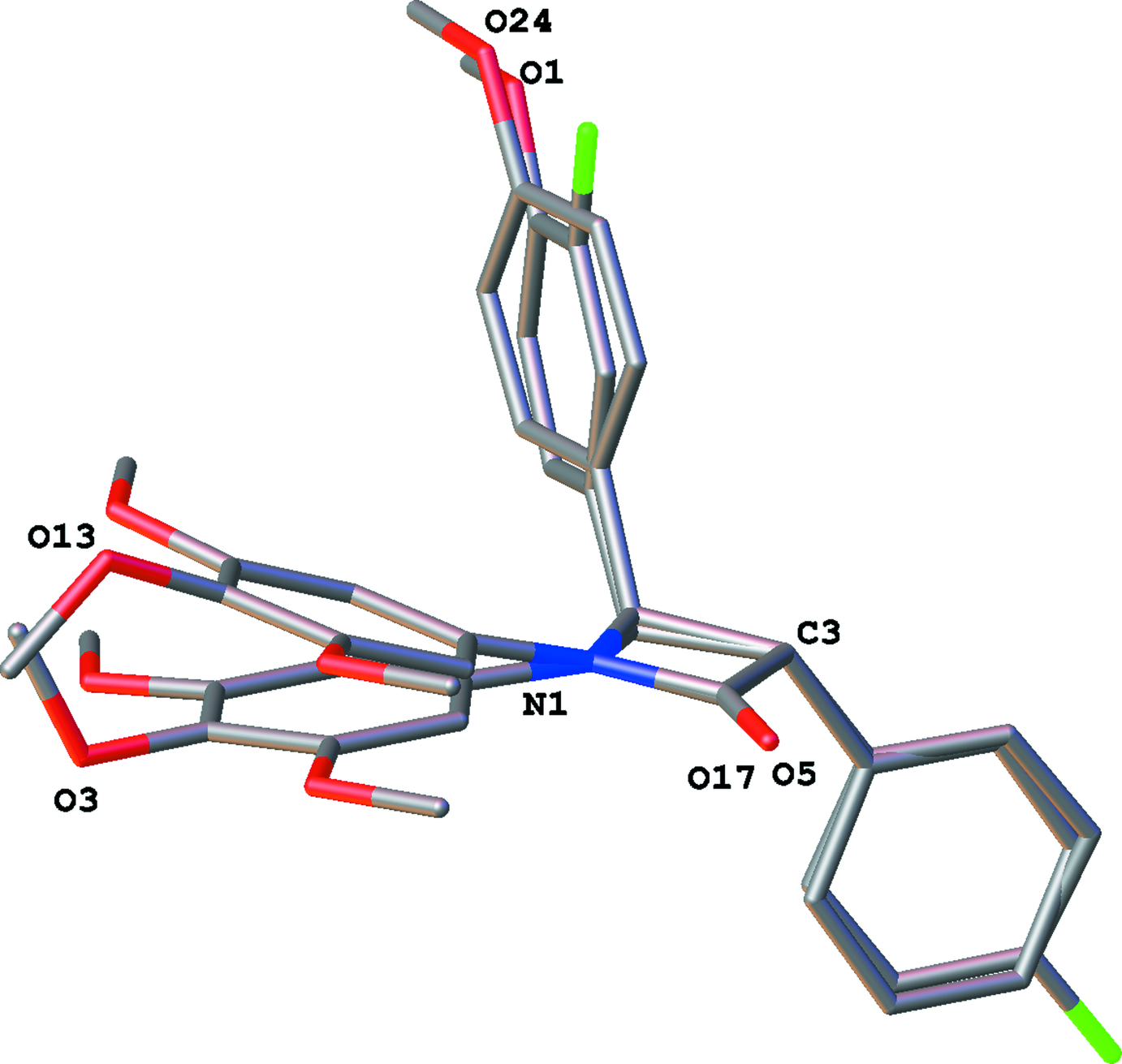

Supplement: Supplementary file 12 [file e-76-01187-sup12.tif]

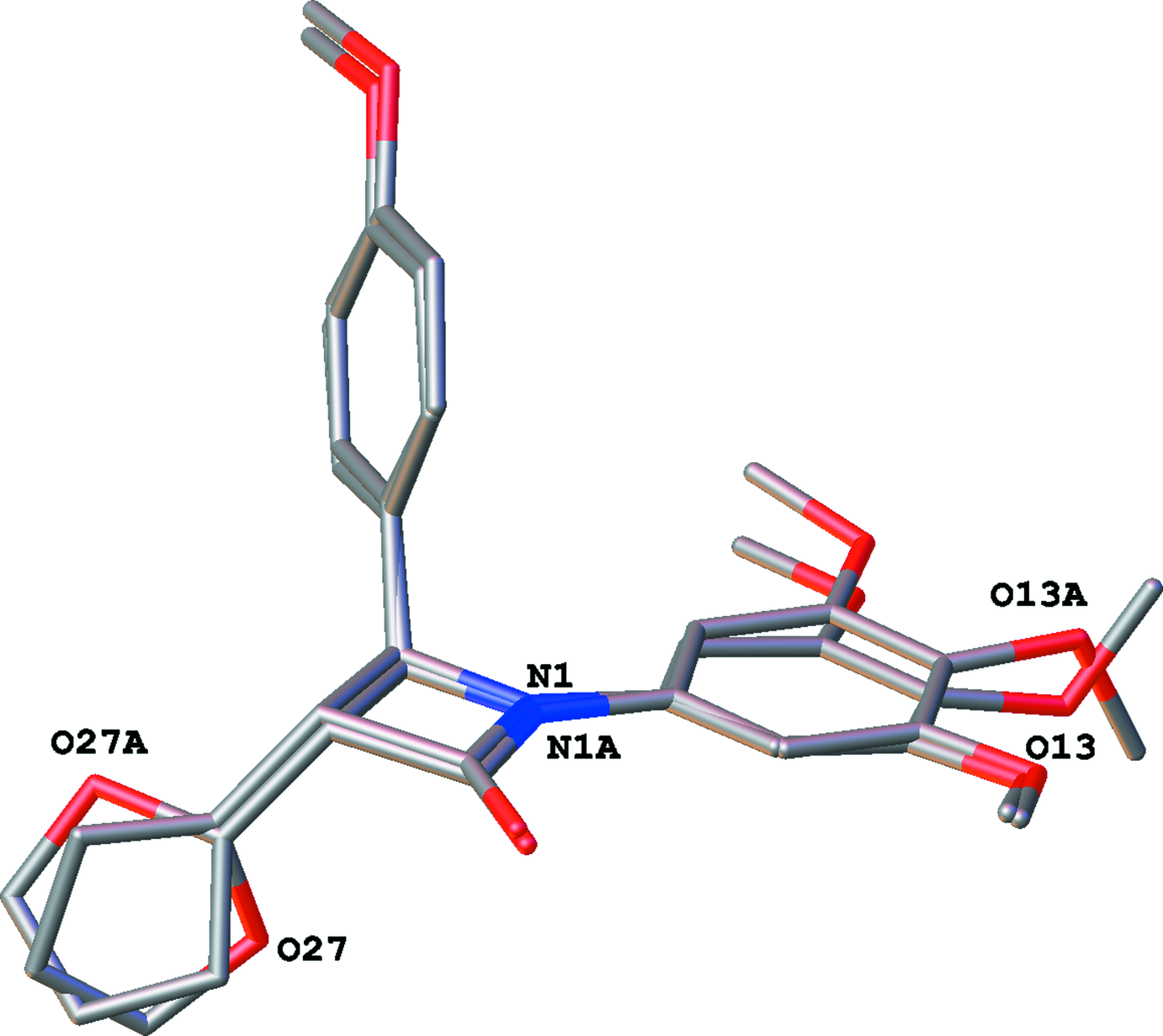

Supplement: Supplementary file 13 [file e-76-01187-sup13.tif]

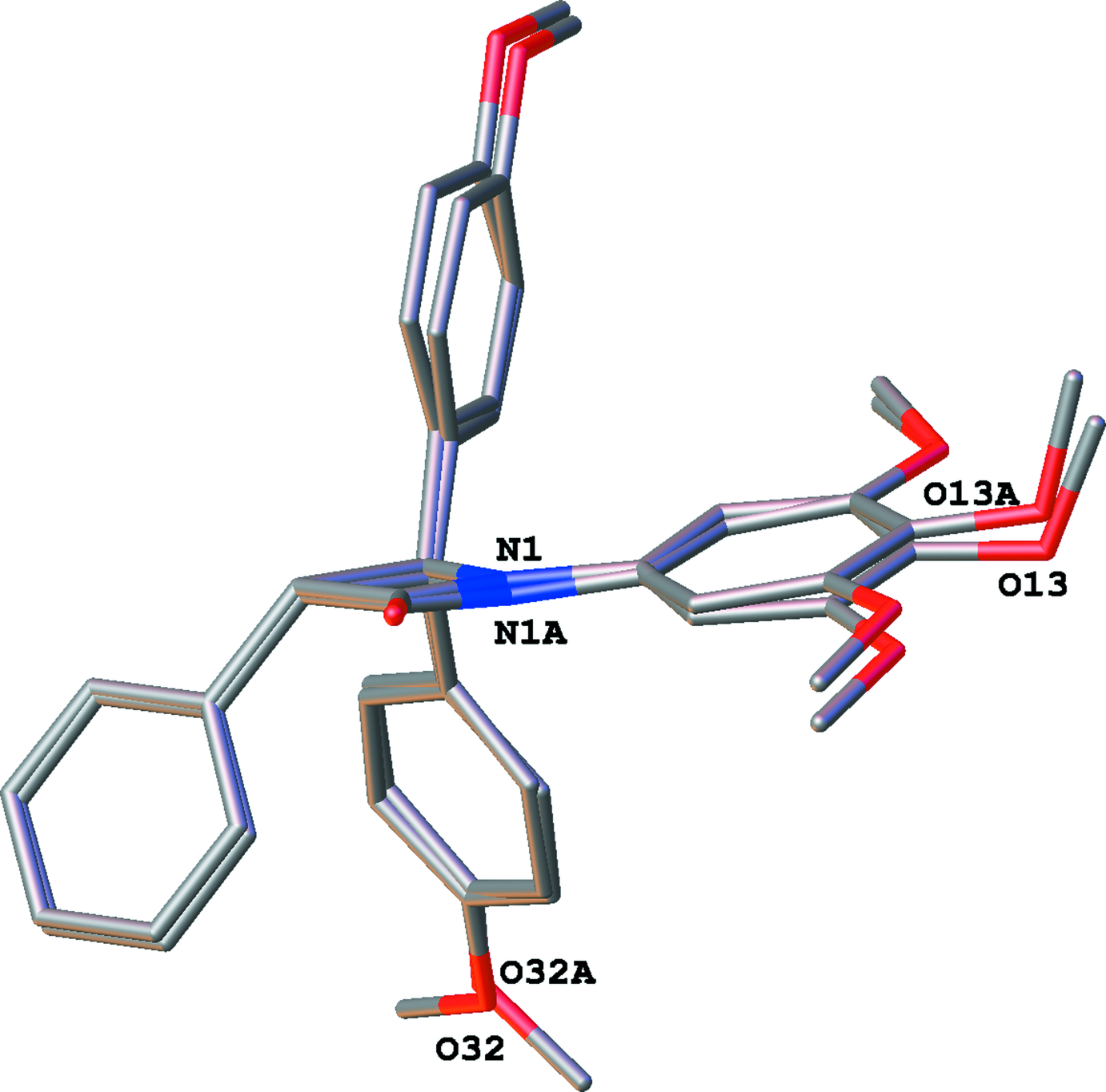

Supplement: Supplementary file 14 [file e-76-01187-sup14.tif]
